# PRAP: Pan Resistome analysis pipeline

**DOI:** 10.1186/s12859-019-3335-y

**Published:** 2020-01-15

**Authors:** Yichen He, Xiujuan Zhou, Ziyan Chen, Xiangyu Deng, Andrew Gehring, Hongyu Ou, Lida Zhang, Xianming Shi

**Affiliations:** 1 Department of Food Science and Technology, MOST-USDA Joint Research Center for Food Safety, School of Agriculture & Biology, and State Key Lab of Microbial Metabolism, Shanghai Jiao Tong University, 800 Dongchuan Road, Shanghai, 200240 China; 2 Center for Food Safety, Department of Food Science and Technology, University of Georgia, Griffin, GA 30223 USA; 3 United States Department of Agriculture, Agricultural Research Service, Eastern Regional Research Center, 600 East Mermaid Lane, Wyndmoor, PA 19038 USA

**Keywords:** Pan-resistome, Identification, Visualization, Machine learning

## Abstract

**Background:**

Antibiotic resistance genes (ARGs) can spread among pathogens via horizontal gene transfer, resulting in imparities in their distribution even within the same species. Therefore, a pan-genome approach to analyzing resistomes is necessary for thoroughly characterizing patterns of ARGs distribution within particular pathogen populations. Software tools are readily available for either ARGs identification or pan-genome analysis, but few exist to combine the two functions.

**Results:**

We developed Pan Resistome Analysis Pipeline (PRAP) for the rapid identification of antibiotic resistance genes from various formats of whole genome sequences based on the CARD or ResFinder databases. Detailed annotations were used to analyze pan-resistome features and characterize distributions of ARGs. The contribution of different alleles to antibiotic resistance was predicted by a random forest classifier. Results of analysis were presented in browsable files along with a variety of visualization options. We demonstrated the performance of PRAP by analyzing the genomes of 26 *Salmonella enterica* isolates from Shanghai, China.

**Conclusions:**

PRAP was effective for identifying ARGs and visualizing pan-resistome features, therefore facilitating pan-genomic investigation of ARGs. This tool has the ability to further excavate potential relationships between antibiotic resistance genes and their phenotypic traits.

## Background

Antibiotics have been used to treat infections, and for prophylaxis as additives in animal feeds for decades. However, the emergence and proliferation of antibiotic resistant bacterial strains has rendered a significant number of antibiotics either ineffective or only marginally effective. A global increase of antibiotic resistance in major pathogens such as *Escherichia coli* and *Salmonella* has been observed [1]. Vertical gene transfer of antibiotic resistance genes (ARGs) goes from parent to offspring, while horizontal gene transfer can occur among different bacterial species or strains via mobile genetic elements that include plasmids, insertion sequences and integrative conjugative elements [2]. Therefore, characterization of ARGs found in a group of pathogens can assist in determining mechanisms of the transmission and distribution of ARGs.

Identification of ARGs contributes to distinguishing and predicting antibiotic resistance phenotypes. However, antibiotic resistance phenotypes do not strictly correspond to a fixed combination of ARGs. For instance, mutations in either of *uphT* or *glpT* gene contribute to fosfomycin resistance in *Staphylococcus aureus* [3]. Alleles of the same acquired ARG may confer resistance to different antibiotics, for example, the *AAC(6′)-Ib* gene has the ability to inactivate aminoglycosides while *AAC(6′)-Ib-cr*, one of its mutated forms, confers fluoroquinolone resistance [4, 5]. Unlike the former, some ARGs may contribute to several types of antibiotic resistance, such as the multidrug efflux genes *oqxAB* that enable olaquindox and ciprofloxacin resistance and *acrAB* genes in *E. coli* that decrease susceptibility to cephalothin and cephaloridine [6, 7]. As a consequence, it would be laborious if only traditional methods, such as polymerase chain reaction, were used for identification of all possible ARGs and their subtypes. In addition, bioinformatics tools are able to rapidly identify ARGs and analyze their characteristics within multiple genomes to reveal potential relationships. Databases like the Antibiotic Resistance Genes Database (ARDB) [8], the Comprehensive Antibiotic Resistance Database (CARD) [9], the Pathosystems Resource Integration Center (PATRIC) [10] and the ResFinder database [11] are used to collect and maintain information on ARGs that can be easily utilized to facilitate bioinformatic analysis. However, substantial diversity in ARGs composition could occur among isolates of the same species due to horizontal gene transfer of mobile genetic elements [12]. This indicates that different ARGs should be analyzed separately to discover their unique features in a given species.

The concept of the “pan-genome” was first proposed in 2005 [13]. Genes within a group of genomes of the same species were categorized into three groups: core, dispensable and strain-specific [13]. Similarly, here we proposed the concept of “pan-resistome”, which referred to the entire ARGs within a group of genomes and is classified into core and accessory resistomes. Pan-resistome analysis may reveal the diversity of acquired ARGs within the group and uncover the prevalence of group-specific ARGs. For instance, an analysis of antimicrobial resistance activities based on orthologous gene clusters indicated that the accessory clusters annotated by CARD exhibited better ability to predict phenotypes than all gene clusters [14]. However, few software tools are currently available to describe characteristics of pan-resistomes. Existing pan-genome analysis tools such as PanOTC [15], ClustAGE [16] and PGAP-X [17] were not specifically developed for ARGs. Other tools such as ARG-ANNOT [18] and KmerResistance [19] focus only on ARGs identification. Therefore, a software tool that combines ARGs identification and pan-genome analysis is needed to facilitate pan-resistome analysis.

In this paper, we presented PRAP (Pan-resistome Analysis Pipeline), an open source pipeline for rapid identification of ARGs, annotation-based characterization of pan-resistomes, and machine learning-guided prediction of ARG contribution to resistance phenotypes. PRAP advances further excavation of potential ARG features and facilitates prediction of antibiotic resistance phenotypes directly from whole genome sequences.

## Implementation

Workflow of PRAP is divided into three parts: preprocessing of input files, identification of ARGs and characterization of the pan-resistome. For input data preprocessing, PRAP accepts numerous formats of sequence files, including raw reads files (fastq), fasta nucleic acid files (fna), fasta amino acid files (faa) and GenBank annotation files (gb). For GenBank annotation files, PRAP extracts protein coding sequences (CDSs) and forms both corresponding fna and faa files.

**Fig. 1:**
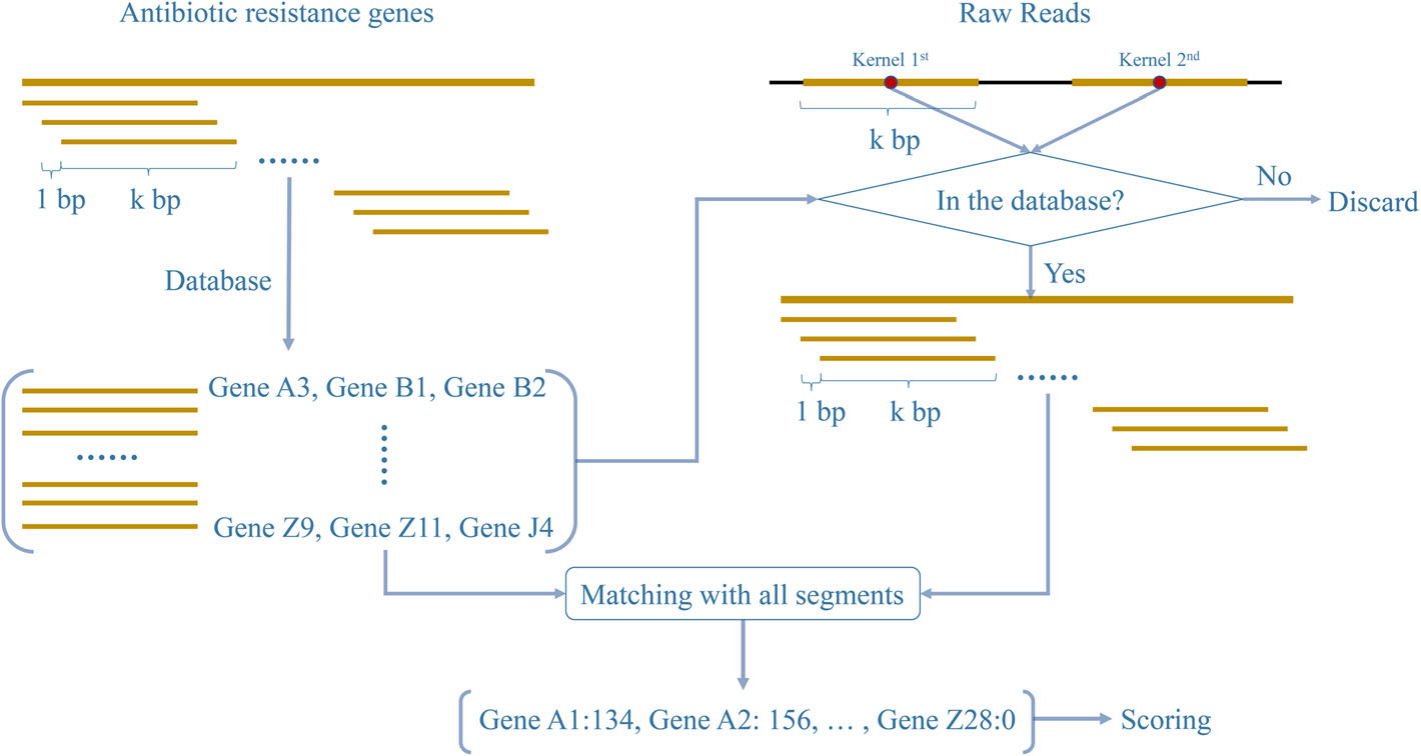
Diagrammatic sketch of k-mer algorithm. Using two kernels as an example

For identification of ARGs, the CARD or ResFinder databases is selected according to user preferences and different methods are used for different formats of input files. For “fastq” files, an assembly-free k-mer method is implemented to locate exact matches between short sequence strings (k-mers) and a pre-defined k-mers library of ARGs [20]. Firstly, ARGs in the original database are segmented into k (user-defined) bp lengths with a step size of 1 bp for both original sequences and reverse complement sequences, and then stored in a temporary database. Secondly, in order to minimize the run time, one, two or three kernels (user-defined) are determined for each read (e.g. one kernel is the middle of a read), and then a kbp length sequence ranging from [kernel-k/2, kernel+k/2] is extracted to determine whether it is in the temporary database. Thirdly, only those filtered reads are segmented into kbp lengths and matched with the temporary database. The diagrammatic sketch of k-mer algorithms is shown in Fig. 1. Scoring for each gene in the database is carried out according to their intersection with all filtered raw reads, and only those higher than the user-defined threshold will be written into results. Lower k values and more kernels (two or three) are recommended when multipoint mutations within individual genes are expected, such as those in *gyrA*, *gyrB*, *parC* and *parE*. Otherwise higher k values and a single kernel are recommended for saving runtime and reducing false positives. For other input data formats, PRAP executes BLAST for query sequences versus the nucleotide or protein sequences as implemented by users. The module parses the results of k-mer or BLAST and forms new output files that contain detailed annotation information.

PRAP’s pan-resistome characterization toolset consists of modules for pan-resistome modeling, ARGs classification, and antibiotics matrices analysis. All these modules use annotation results from the ARGs identification module as input.

The pan-resistome modeling module can be used to characterize the distribution of ARGs among the input genomes. It traverses all possible combinations (${C}_N^k$) (N refers to the total number of genomes and *k* refers to the number of genomes selected in each combination) of genomes to extrapolate the number of ARGs in the pan and core resistomes. Note that grouping orthologous genes according to sequence identity is not carried out, but alleles of the same ARG are regarded as orthologous genes. An orthologous genes cluster is categorized into core resistomes if it presents in all the input genomes, otherwise it is divided into accessory resistomes. The choice of fitting model for pan and core resistomes size extrapolation is user-defined. One of the models provided is a “polynomial model” that accesses fitness within a given interval. However, as a consequence of over-fitting, the trend may be incorrect after exceeding the interval of input genomes. Another “power law regression” model can overcome this shortcoming but may not be appropriate when the number of genomes is small [21]. Thus, PRAP uses a coverage parameter that can be modified in the configuration file to determine the curve-fitting percentage. In addition, the model proposed by the PanGP platform is also provided [22].

The ARGs classification module outputs summary statistics of classified ARGs in both pan and accessory resistomes, because ARGs in core resistomes may lead to indistinguishable differences if only analyzing the pan-resistome. A stacked bar graph together with a cluster map shows the quantity and relationships of the associated genes for each type of antibiotic. A comparison matrix graph with *n*^*2*^ (*n* is the number of genomes) subgraphs is drawn and each subgraph represents comparison of ARGs from two genomes.

**Fig. 2:**
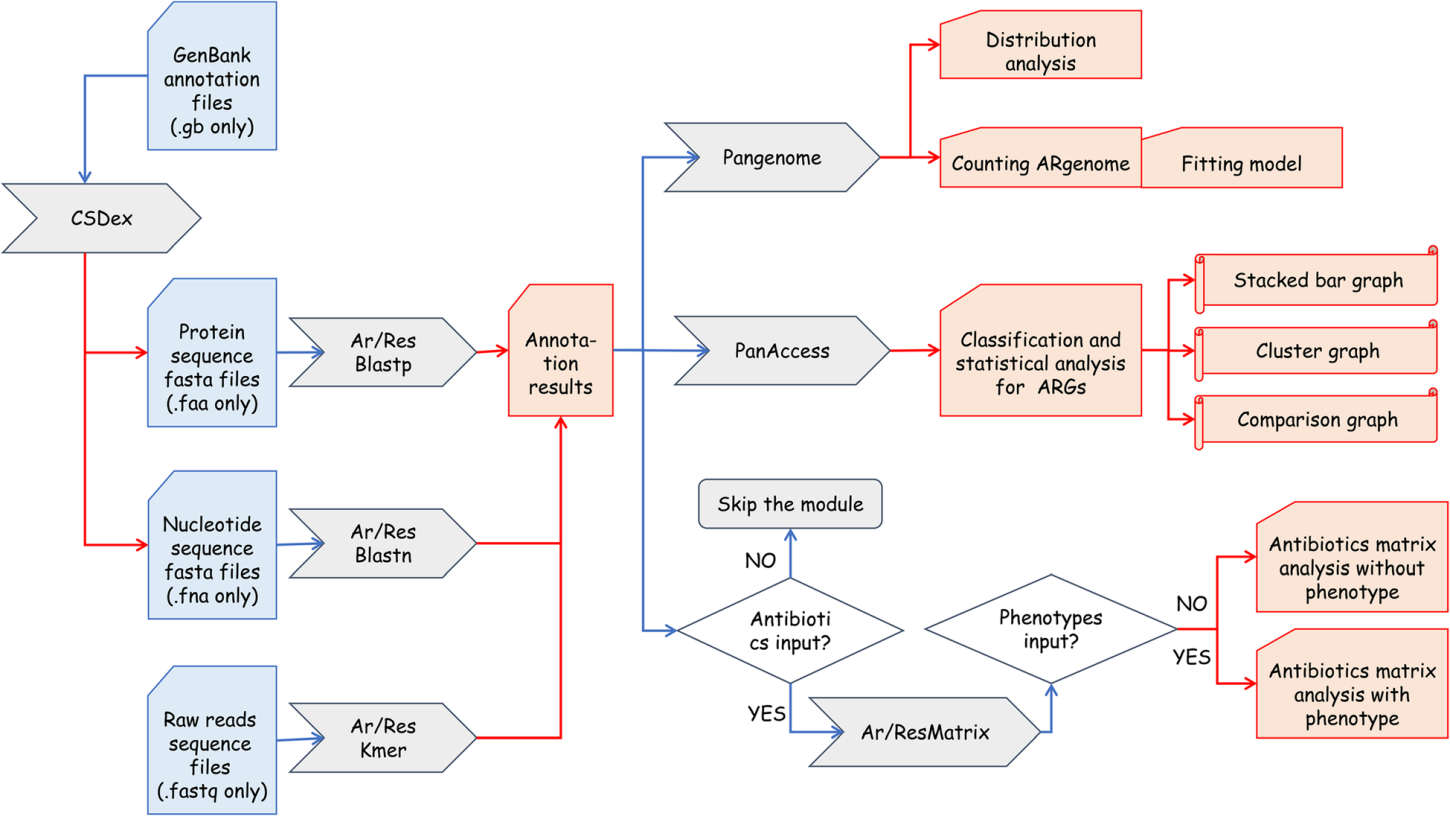
PRAP workflow. The input files and steps are shown in blue and output files and steps are shown in red. The cells in gray represent the PRAP modules

The antibiotics matrices analysis module presents associated ARGs for each type of antibiotic as individual cluster maps. If resistance phenotypes are provided, the contribution of each gene to the resistance of given antibiotics will be calculated via a machine learning classifier that uses the random forest algorithm. An overview of PRAP workflow is shown in Fig. 2. A detailed user manual is available in the GitHub repository of PRAP (https://github.com/syyrjx-hyc/PRAP).

## Results

### Data sets for performance evaluation

To test the performance of PRAP, we used genome sequences and antimicrobial susceptibility testing results of 26 *Salmonella enterica* isolates of three different serotypes (*S. Indiana*, *S.* Typhimurium and *S.* Enteritidis). The isolates were obtained from food and clinical sources in Shanghai, China. The genomes of the isolates were sequenced using an Illumina Hiseq platform and sequencing reads were assembled using SOAPdenovo and GapCloser. Assembled genomes were submitted via the submission Portal to NCBI and annotated by the Prokaryotic Genome Annotation Pipeline where the GenBank annotation files were downloaded as part of the input files. Minimum inhibitory concentrations (MIC) of antibiotics were determined by the agar dilution method as recommended by the Clinical and Laboratory Standard Institute. Detailed information about the isolates is available in Additional file 1.

### Comparison of different gene identification methods

In order to compare different ARGs identification methods, we used the input files containing raw sequencing reads, draft genome assemblies, CDSs and protein sequences extracted from GenBank files. The k-mer and BLAST methods based on different databases were implemented simultaneously to handle various input files. Metrics for performance evaluation included the simple matching coefficient (SMC) = (TP + FP)/N_alleles_, Matthews’ correlation coefficient (MCC) = (TP × TN-FP × FN)/ $\sqrt{\left(\mathrm{TP}+\mathrm{FP}\right)\left(\mathrm{TN}+\mathrm{FN}\right)\left(\mathrm{TP}+\mathrm{FN}\right)\left(\mathrm{TN}+\mathrm{FP}\right)}$ and runtime (Table 1). Metrics were calculated based on acquired ARGs for the ResFinder database and all ARGs for the CARD. The k-mer method worked best when using the CARD database with the average turnaround time of 1 min per genome, and BLAST worked best on the ResFinder database by averaging 3 s per genome. Files generated by the k-mer method are available in Additional file 2, and various annotation results based on different methods and databases are available in Additional file 3.

**Table 1:** Performance of different methods for ARGs identification

Input Format	Database	SMC	MCC	Runtime (min)
Raw Reads	CARD	0.9638	0.9809	25
Scaffolds	CARD	0.8954	0.9440	< 1
CDSs	CARD	0.9600	0.9789	< 1
Proteins	CARD	0.9532	0.9347	30
Raw Reads	ResFinder	0.9345	0.9649	25
Scaffolds	ResFinder	0.9924	0.9960	< 1
CDSs	ResFinder	0.9899	0.9946	< 1
Proteins	ResFinder	0.9647	0.9812	30

Parameters for the k-mer method included a k value of 25, two searching kernels, a depth of 20 and at least 100 area score and 90% coverage by length. Parameters for BLASTn and BLASTp included 95% identity for BLASTn and 98% identity for BLASTp and at least 90% coverage by length for both. Runtime is the time consumed for analyzing 26 genomes

### Pan-resistome modeling

**Fig. 3:**
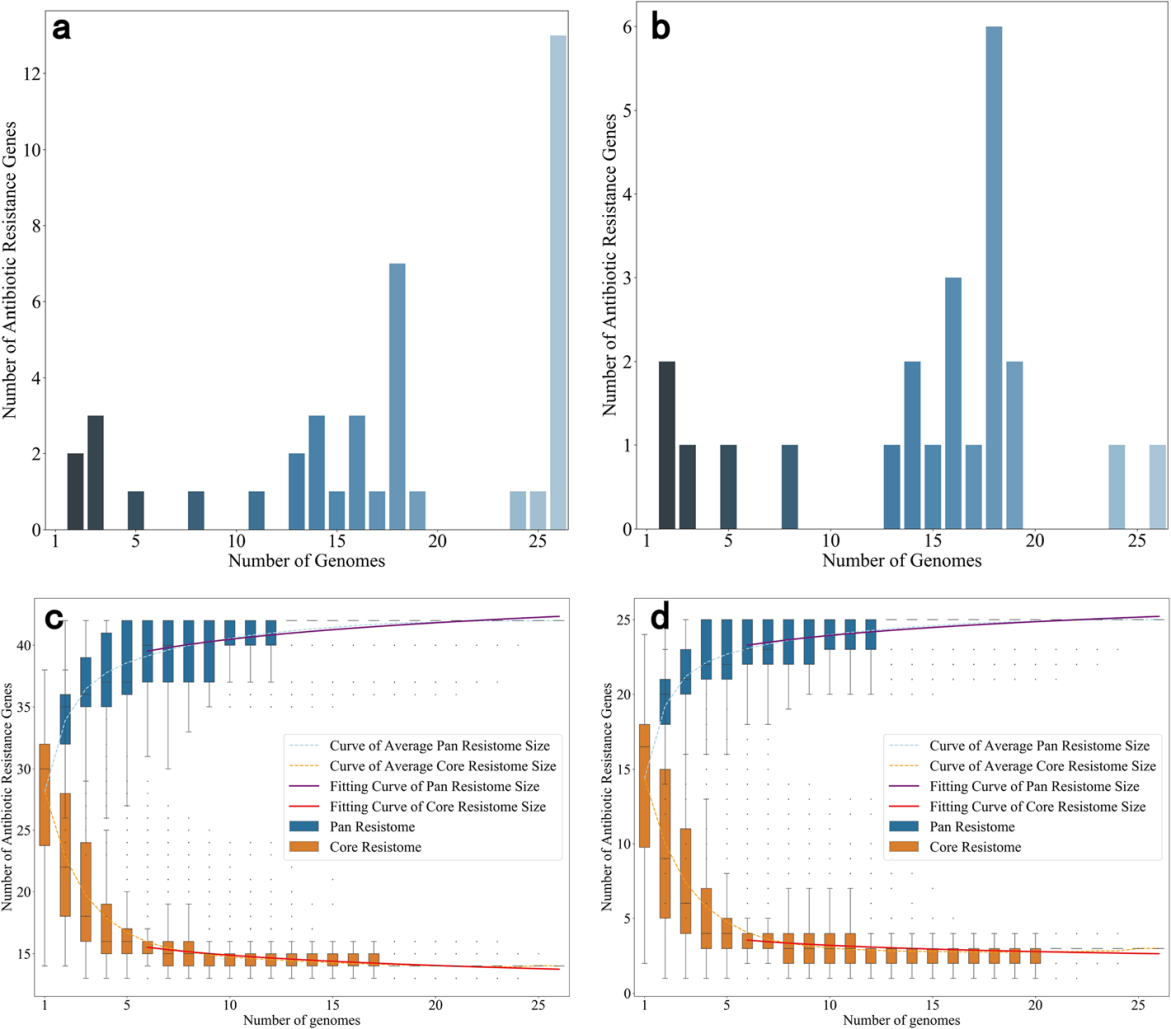
Features of the pan-resistome. **a** ARGs distribution based on the CARD. **b** ARGs distribution based on the ResFinder database **c** Models of pan and core resistomes based on the CARD. **d** Models of pan and core resistomes based on the ResFinder database

Pan-resistome modeling was based on the annotation results from the previous step for both CARD and ResFinder databases. The resistomes identified with CARD contained 13 core ARGs (Fig. 3a), greater than the single core ARG identified with ResFinder (Fig. 3b). This difference was likely caused by the fact that ResFinder database only included acquired ARGs instead of all resistance conferring genes and mutations in the core resistomes. The only core gene from acquired ARGs belonged to the *AAC(6′)* family. The power law model with a fitting coverage of 80% was used for modeling the pan-resistome size curve. The models of pan-resistome size were *P* = 36.3310 × ^0.04699^ (*R*^2^ = 0.9534) for CARD (Fig. 3c) and *P* = 21.1194 × ^0.0544^ (*R*^2^ = 0.9637) for ResFinder (Fig. 3d). The results suggested that these *S. enterica* isolates had an open pan-resistome, revealing the high likelihood of *S. enterica* to acquire foreign ARGs.

### ARGs classification

**Fig. 4:**
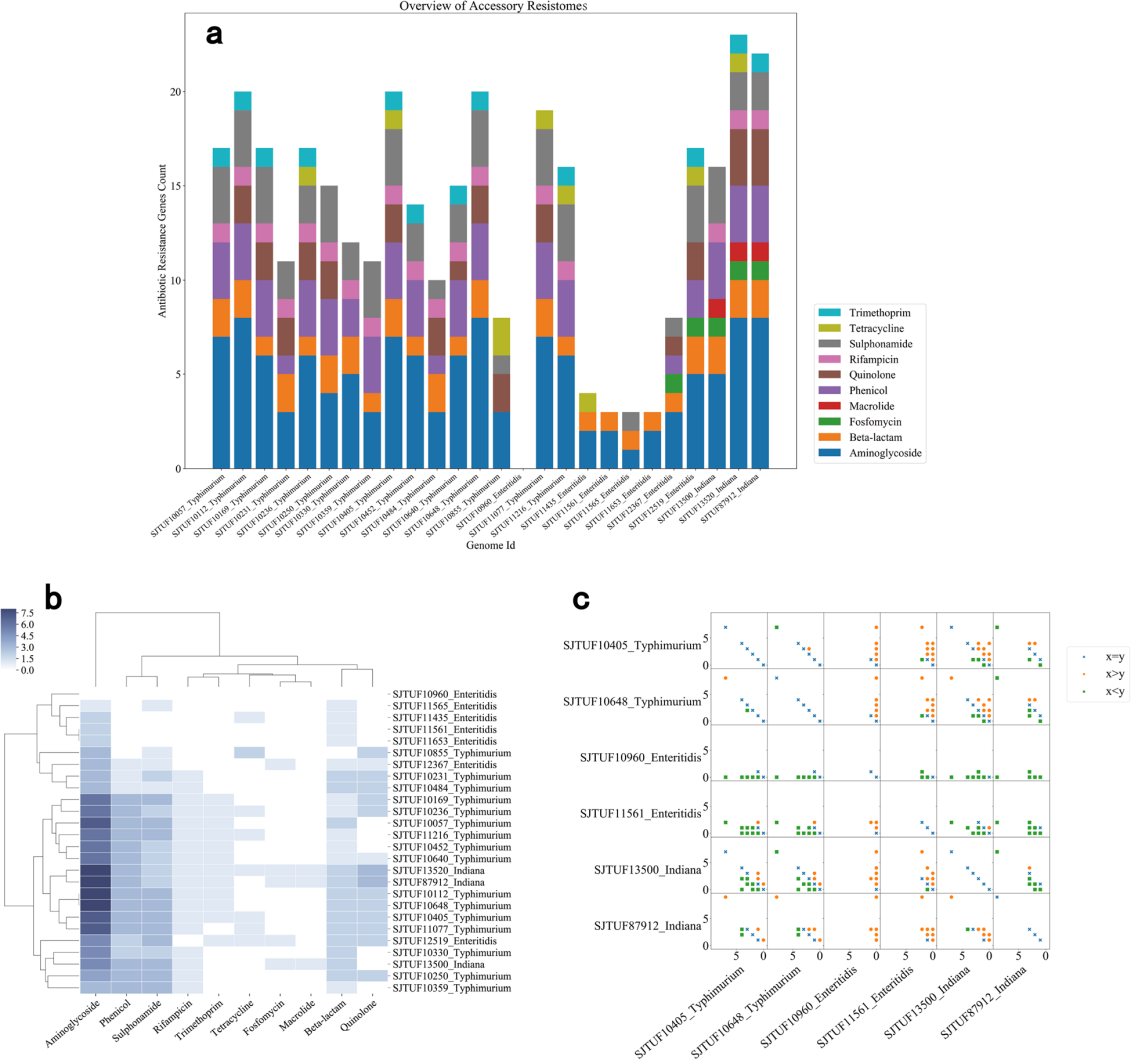
Characteristics of the accessory resistomes based on the ResFinder database. **a** Total counts of antibiotic resistance genes for individual strains of *S. enterica* serotypes. The different colors correspond to different antibiotics shown in the legend. **b** Clustering results of the accessory resistomes. The darker the color, the greater the number of related genes. **c** Comparison matrix of accessory ARGs within each genome. Each symbol represents the number of genes related to a specific antibiotic. The blue symbols indicate that the genomes on the x-axis and the y-axis have equal numbers of genes (n_x_ = n_y_), while green for n_x_ < n_y_ and orange for n_x_ > n_y_. If the number of the two genomes is equal, all the symbols will be arranged on the diagonal, otherwise significant shifts will deviate substantially from the diagonal

To compare the compositions of acquired ARGs of the three different serotypes of *S. enterica*, we identified accessory resistomes using the ResFinder database. The total counts (Fig. 4a) and clustering (Fig. 4b) of the accessory resistomes illustrated the discrepancy of the resistance of different serotypes or strains to individual antibiotics. *S.* Typhimurium and *S. Indiana* possessed more ARGs than that of *S.* Enteritidis. A pairwise comparison of accessory ARGs for each genome further confirmed this (Fig. 4c, partially shown). With respect to the different antibiotics, these 26 *S. enterica* isolates possessed more genes that conferred aminoglycoside resistance compared with other types of resistance phenotypes.

### Antibiotic matrices analysis

**Fig. 5:**
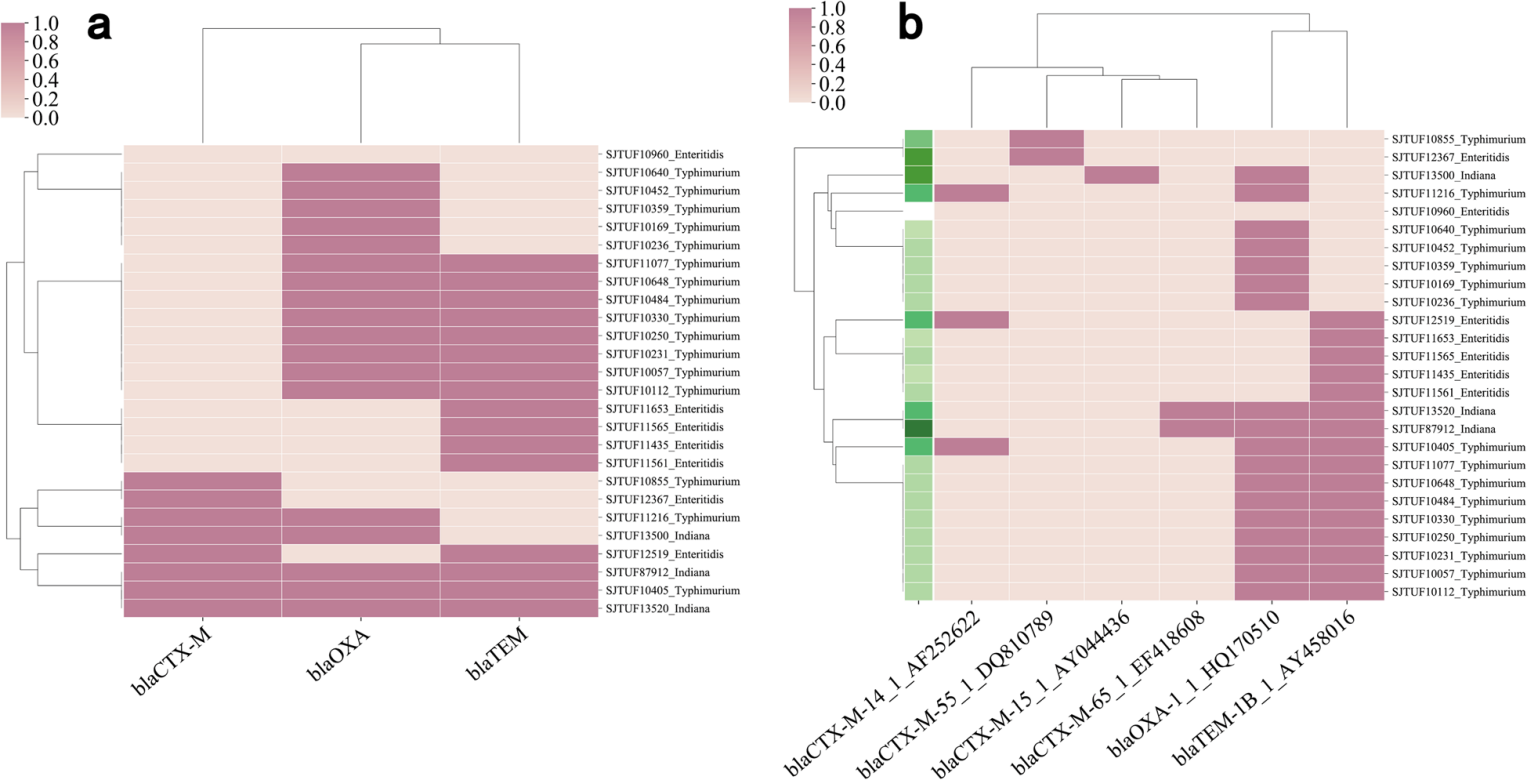
Matrix analysis of β-lactam antibiotics based on the ResFinder database. **a** Clustering results of ARGs that were associated with β-lactam resistance with the “allele” parameter. **b** Clustering results of ARGs that were associated with β-lactam resistance with the “detailed” parameter, together with user-provided phenotypes of β-lactam antibiotic resistance results. The deeper the color, the greater number of antibiotics to which the isolate is resistant

The accessory resistomes identified by the ResFinder database were then analyzed for their correlated resistance phenotypes. For example, the “β-lactam” results included the presence of all genes related to resistance of β-lactam antibiotics in each genome and a cluster map was drawn according to the matrix (Fig. 5a and b). For 26 *S. enterica* isolates, ARGs that confer β-lactam resistance contained the alleles of CTX-M, OXA and TEM (Fig. 5a) and this included subtypes for the multiple CTX-M genes (Fig.5b). The resistance phenotypes could be shown in front of the matrix if raw phenotype data were provided (Fig.5b). In the example, the β-lactam resistance phenotypes were positively correlated with the genotype in most circumstances although there were exceptions for SJTUF10855 and SJTUF12367. Prediction of the highest contribution value of alleles to aminoglycoside, β-lactam, phenicol, sulfonamide and tetracycline resistance were *aph(3′)* (14.71%), *blaCTX-M* (21.58%), *floR* (24.54%), *catB* (14.18%) and *tet* (22.35%), respectively. Detailed output results are available in Additional file 4.

## Discussion

For the ARGs identification module of PRAP, the k-mer method was used only for the selection of the most likely allele with the highest score and coverage from each type of ARG, resulting in a relatively lower recall rate when more than one orthologous ARG existed in a genome. For BLAST methods, the use of protein sequences might lead to poor discrimination among alleles for each type of ARG because different alleles may have identical amino acid products. For example, *blaTEM-1* has four genotypes that include *blaTEM-1A, B, C* and *D* in the ResFinder database, which have identical amino acid sequences but different nucleotide sequences. The use of nucleotide sequences could avoid this problem and yield a lower false positive rate at the subtype level.

With respect to the prediction of contribution of ARGs, results showed that most of the predicted ARGs conferred resistance to related antibiotics. However, *catB* was not related to sulfonamide antibiotic resistance but conferred phenicol antibiotic resistance [9]. The primary reason for this deviation was that the sulfonamide antibiotic resistance phenotypes in the data sets did not differ significantly among different isolates. Therefore, users should provide highly differentiated phenotype data to minimize the Gini impurity in the random forest algorithm, so as to avoid spurious correlation in the final prediction of the contribution value.

The output of PRAP is of high significance in understanding the antibiotic resistance abilities among different stains and for surveillance of antibiotic resistance conditions in foodborne pathogens. It could be further utilized to mine relationships between genomic features and antibiotic resistance phenotypes and build corresponding prediction models, since numerous genomes together with their antimicrobial susceptibility testing results were available in the PARTIC database. These prediction models could also be included as a functional module in a future version of PRAP, which would contribute to the real-time prediction of antibiotic resistance phenotypes.

## Conclusions

We have proposed the concept of “pan-resistome” and developed an effective, easy to install and convenient to use tool (PRAP) that characterizes the bacterial pan-resistome. PRAP works with multiple genome file formats and identifies ARGs from them based on the CARD and ResFinder databases according to user preferences. Further analysis implemented by PRAP can excavate antibiotic resistance features within the total studied population and distinguish differences among individual isolates, rendering the results through intuitive visualization. In brief, PRAP facilitates rapid identification of ARGs from multiple genome files and discovery of potential ‘laws’ of ARGs transmission and distribution within the population.

### Availability and requirements

**Project name:** PRAP.

**Project home page:** https://github.com/syyrjx-hyc/PRAP

**Operating system(s):** Platform independent.

**Programming language:** Python3.

**Other requirements:** Python v3.5 or higher, BLAST+ v2.7.1 or higher.

**License:** GNU GPL v3.

**Any restrictions to use by non-academics:** None.

## Supplementary information

**Additional file 1. **Information for 26 *S. enterica* genomes.

**Additional file 2.** Archive containing files for evaluation k-mer performance and scoring generated by the k-mer method.

**Additional file 3. **Archive containing results of annotation of different formats of genome files for 26 *S. enterica* genomes based on both the CARD and ResFinder databases.

**Additional file 4. **Archive containing results of analysis for nucleotide sequences of 26 *S. enterica* genomes annotated by the ResFinder database.

## Data Availability

The software is available on GitHub (https://github.com/syyrjx-hyc/PRAP) and the test data sets are available in the NCBI genome repositories (https://www.ncbi.nlm.nih.gov/genome). The GenBank accession numbers of 26 *S. enterica* genomes are listed below, which are also available in Addition file 1: GCA_004324145.1, GCA_004324315.1, GCA_004324275.1, GCA_004324135.1, GCA_004324125.1, GCA_004324115.1, GCA_004324095.1, GCA_004324045.1, GCA_004337745.1, GCA_004324035.1, GCA_004324025.1, GCA_004324015.1, GCA_004324245.1, GCA_004324235.1, GCA_004337755.1, GCA_004323995.1, GCA_004337735.1, GCA_004323935.1, GCA_004323945.1, GCA_004324225.1, GCA_004323925.1, GCA_004323915.1, GCA_004323815.1, GCA_004324215.1, GCA_004323855.1 and GCA_004324195.1.

## Abbreviations

ARGs: Antibiotic resistance genes
CARD: Comprehensive Antibiotic Resistance Database
MCC: Matthews’ correlation coefficient
SMC: Simple matching coefficient
