# Supplementary figures and images for: PRAP: Pan Resistome analysis pipeline (part 2 of 3)
Source: BMC Bioinformatics. 2020 Jan 15;21:20. doi: 10.1186/s12859-019-3335-y (PMC6964052; doi:10.1186/s12859-019-3335-y)

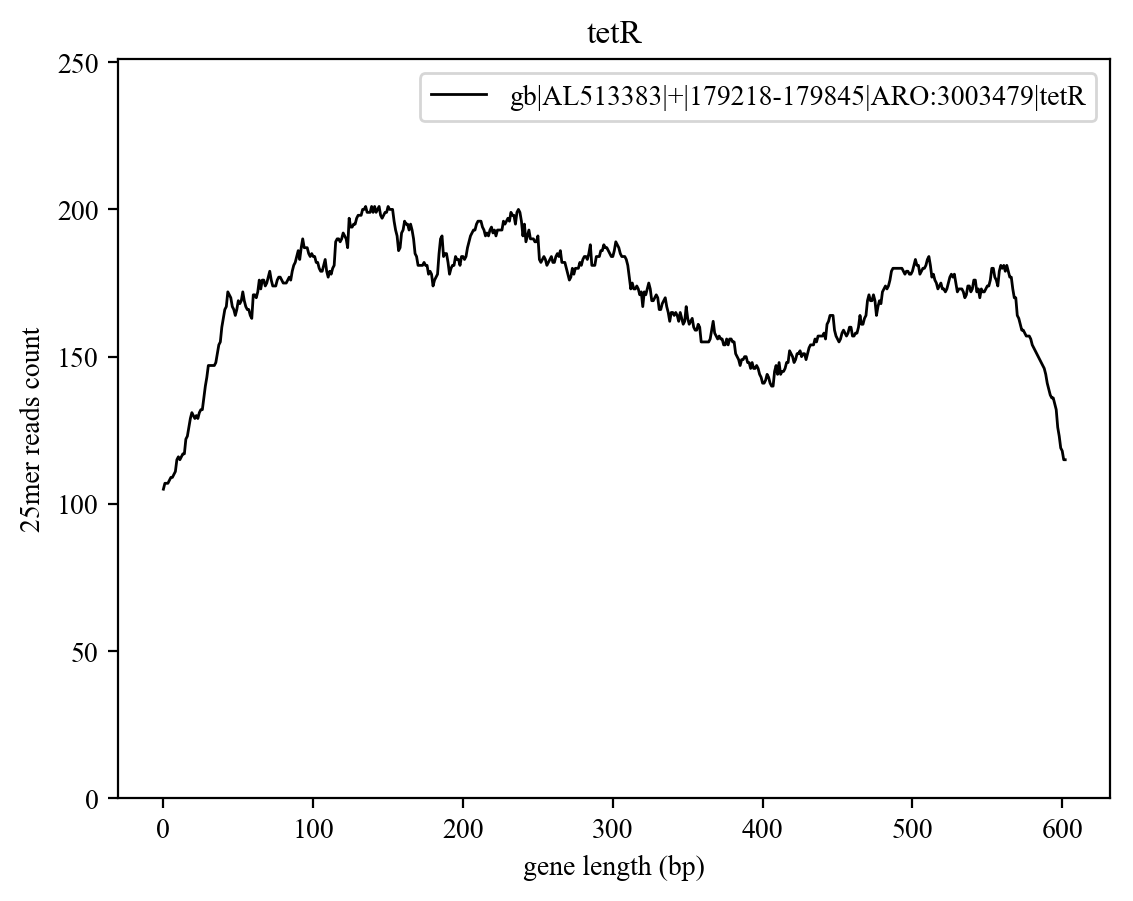

Supplement: Supplementary file 2 — Additional file 2. Archive containing files for evaluation k-mer performance and scoring generated by the k-mer method. [file 12859_2019_3335_MOESM2_ESM.zip › kmer/SJTUF10169_Typhimurium/ar_nucl_25/tetR_25mer.png]

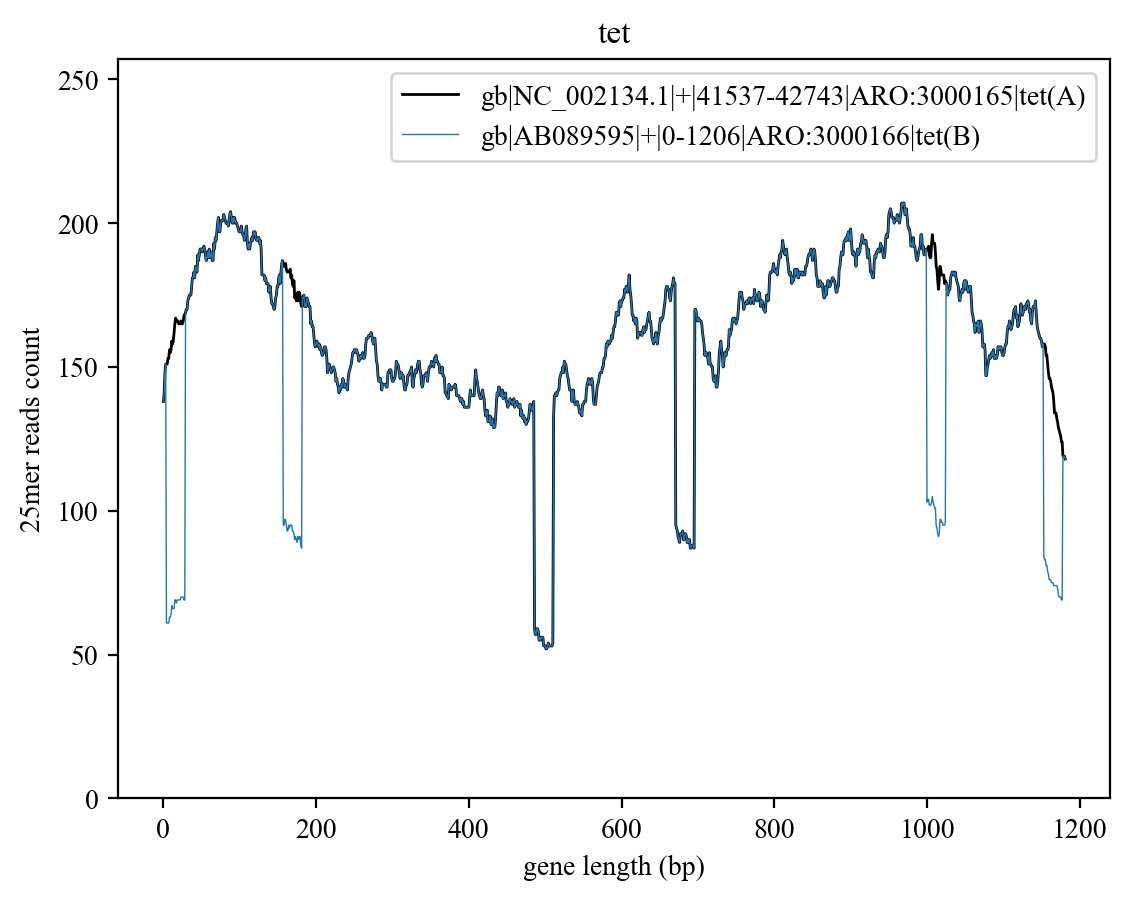

Supplement: Supplementary file 2 — Additional file 2. Archive containing files for evaluation k-mer performance and scoring generated by the k-mer method. [file 12859_2019_3335_MOESM2_ESM.zip › kmer/SJTUF10169_Typhimurium/ar_nucl_25/tet_25mer.png]

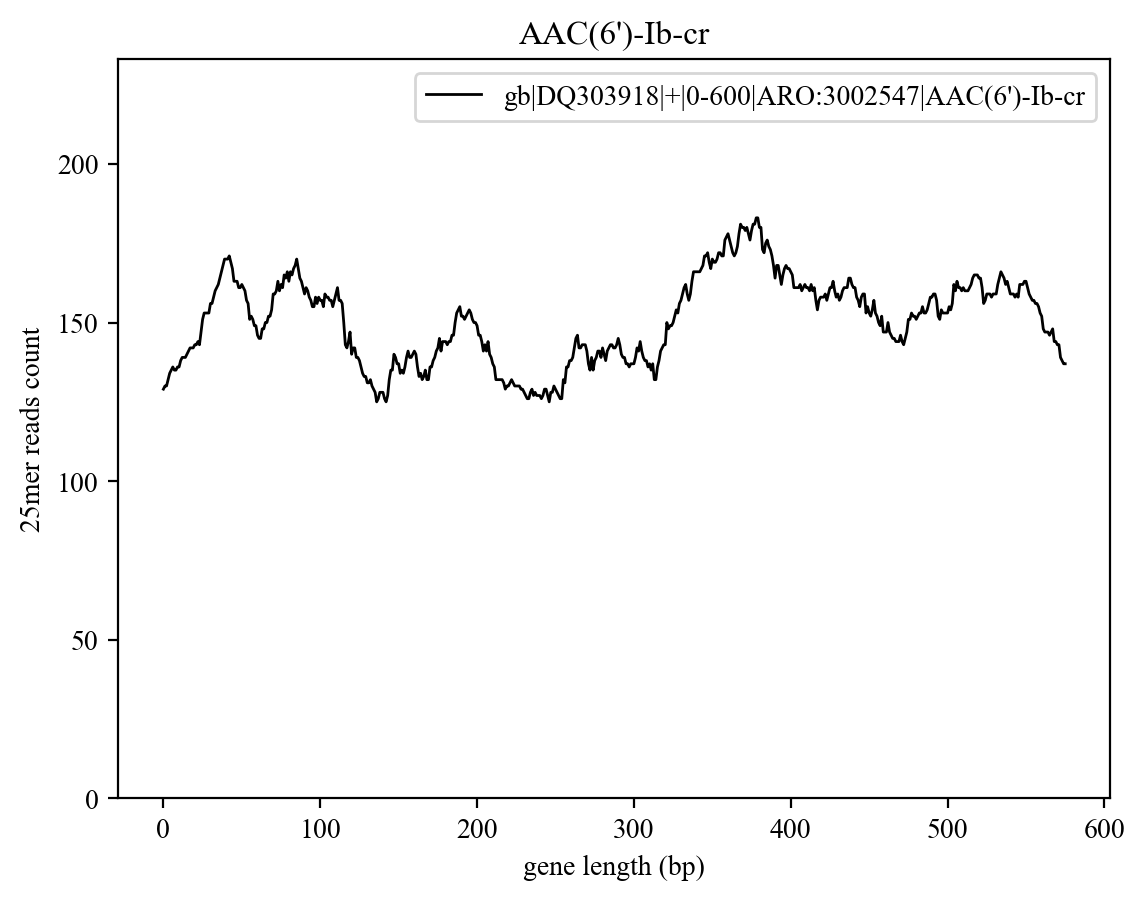

Supplement: Supplementary file 2 — Additional file 2. Archive containing files for evaluation k-mer performance and scoring generated by the k-mer method. [file 12859_2019_3335_MOESM2_ESM.zip › kmer/SJTUF10231_Typhimurium/ar_nucl_25/AAC(6')-Ib-cr_25mer.png]

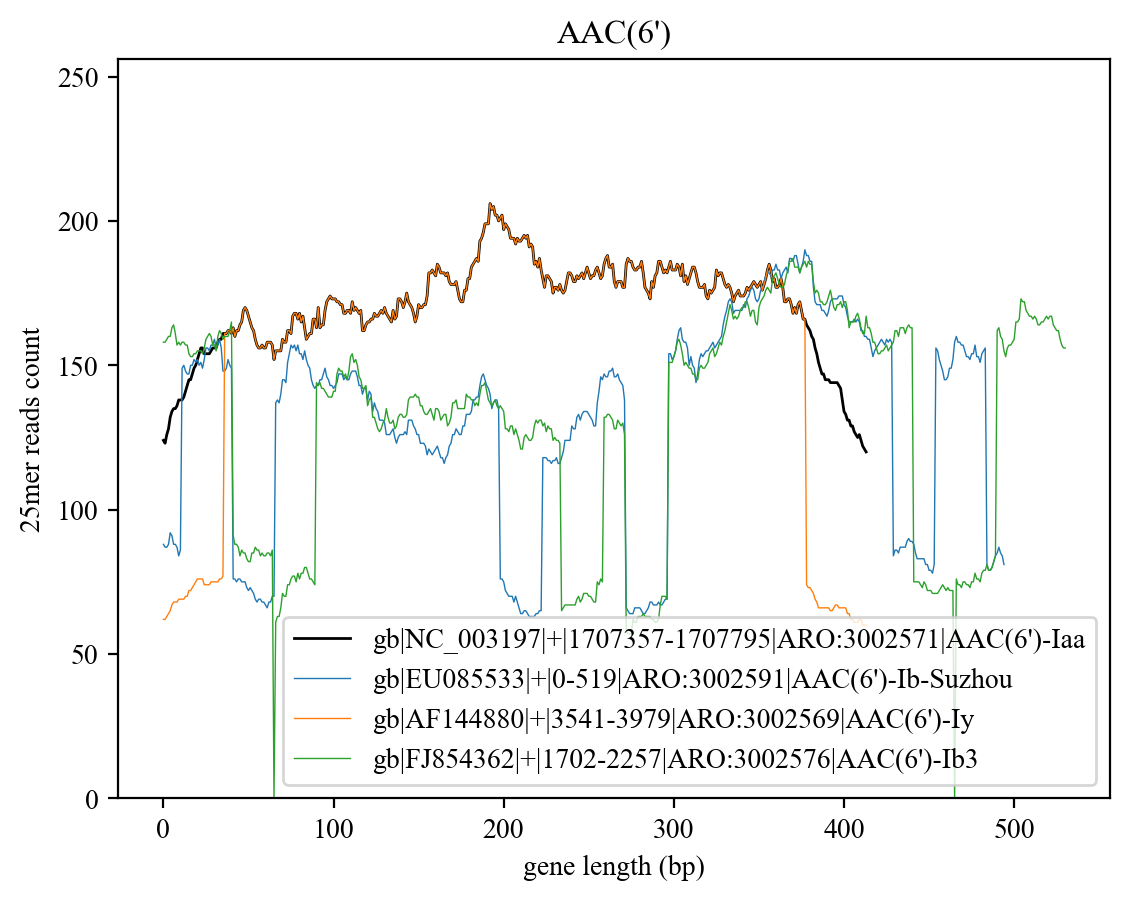

Supplement: Supplementary file 2 — Additional file 2. Archive containing files for evaluation k-mer performance and scoring generated by the k-mer method. [file 12859_2019_3335_MOESM2_ESM.zip › kmer/SJTUF10231_Typhimurium/ar_nucl_25/AAC(6')_25mer.png]

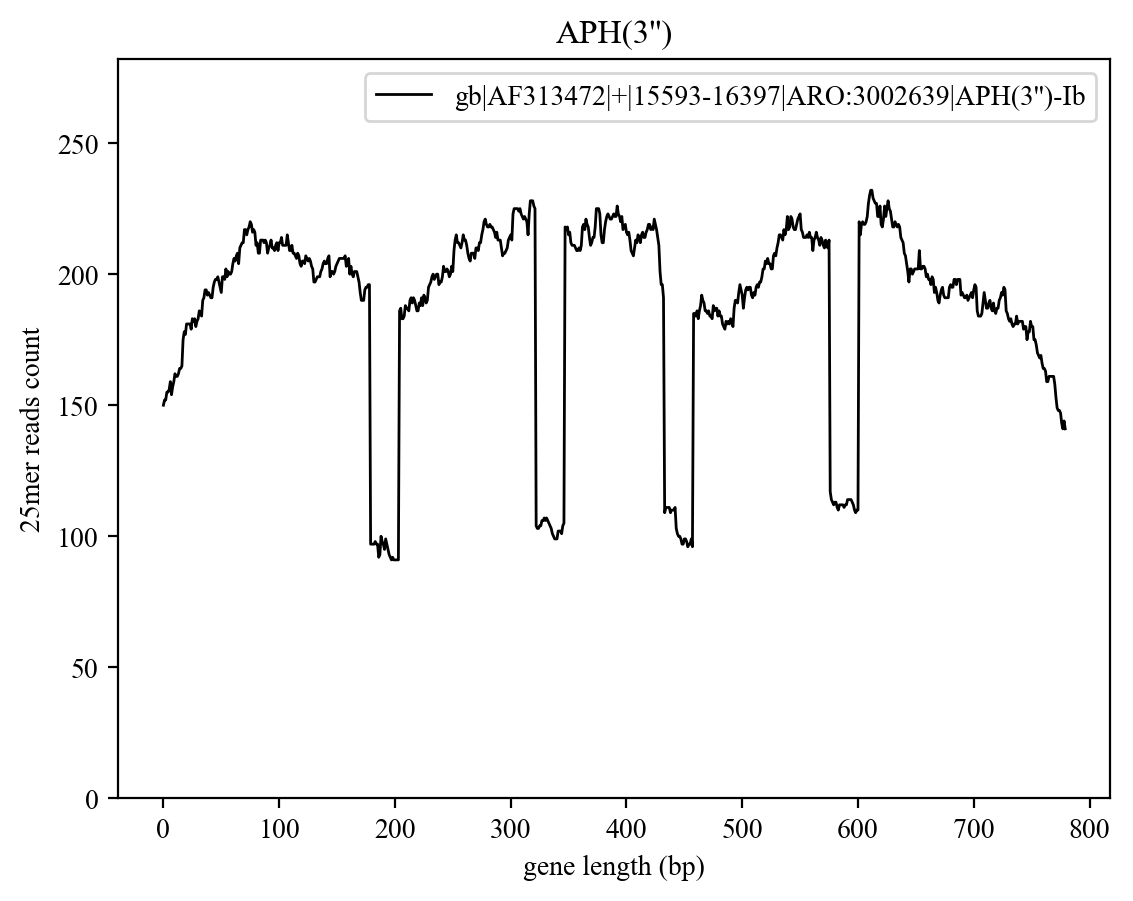

Supplement: Supplementary file 2 — Additional file 2. Archive containing files for evaluation k-mer performance and scoring generated by the k-mer method. [file 12859_2019_3335_MOESM2_ESM.zip › kmer/SJTUF10231_Typhimurium/ar_nucl_25/APH(3'')_25mer.png]

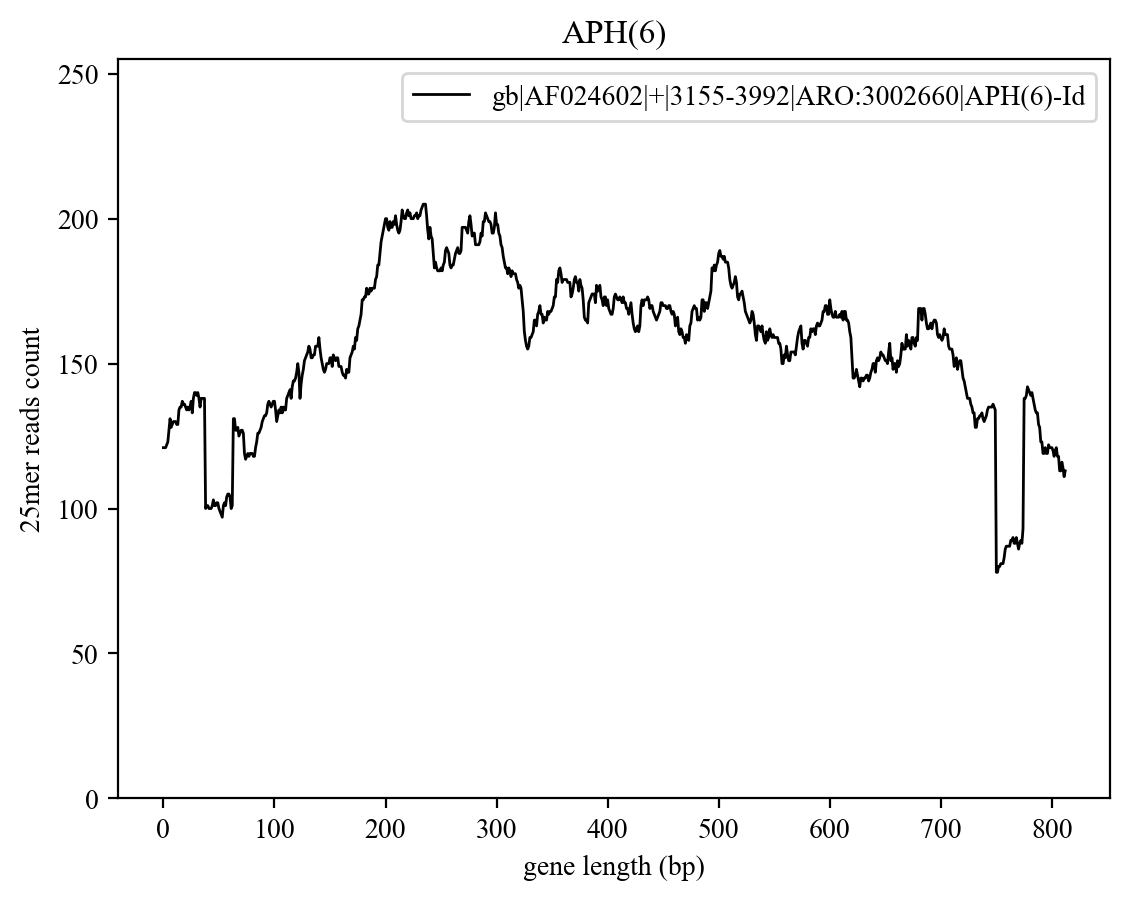

Supplement: Supplementary file 2 — Additional file 2. Archive containing files for evaluation k-mer performance and scoring generated by the k-mer method. [file 12859_2019_3335_MOESM2_ESM.zip › kmer/SJTUF10231_Typhimurium/ar_nucl_25/APH(6)_25mer.png]

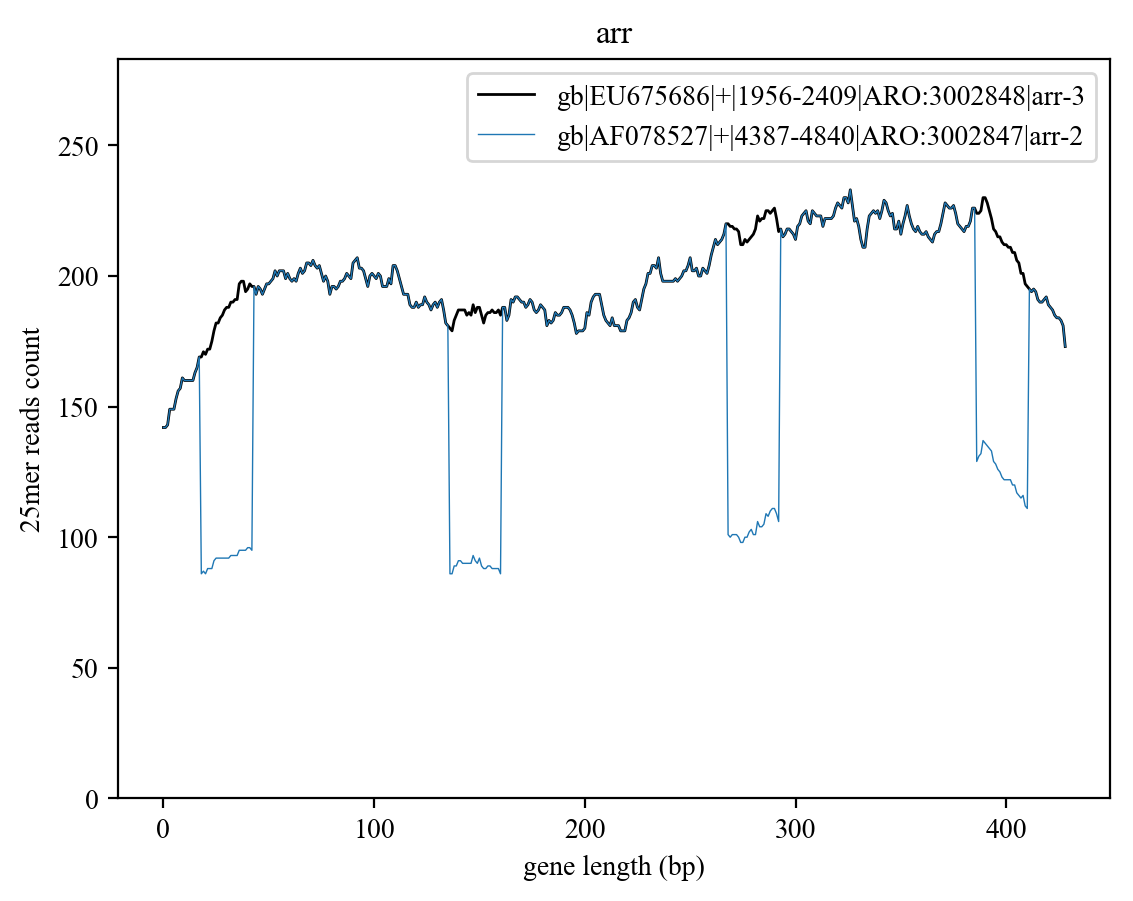

Supplement: Supplementary file 2 — Additional file 2. Archive containing files for evaluation k-mer performance and scoring generated by the k-mer method. [file 12859_2019_3335_MOESM2_ESM.zip › kmer/SJTUF10231_Typhimurium/ar_nucl_25/arr_25mer.png]

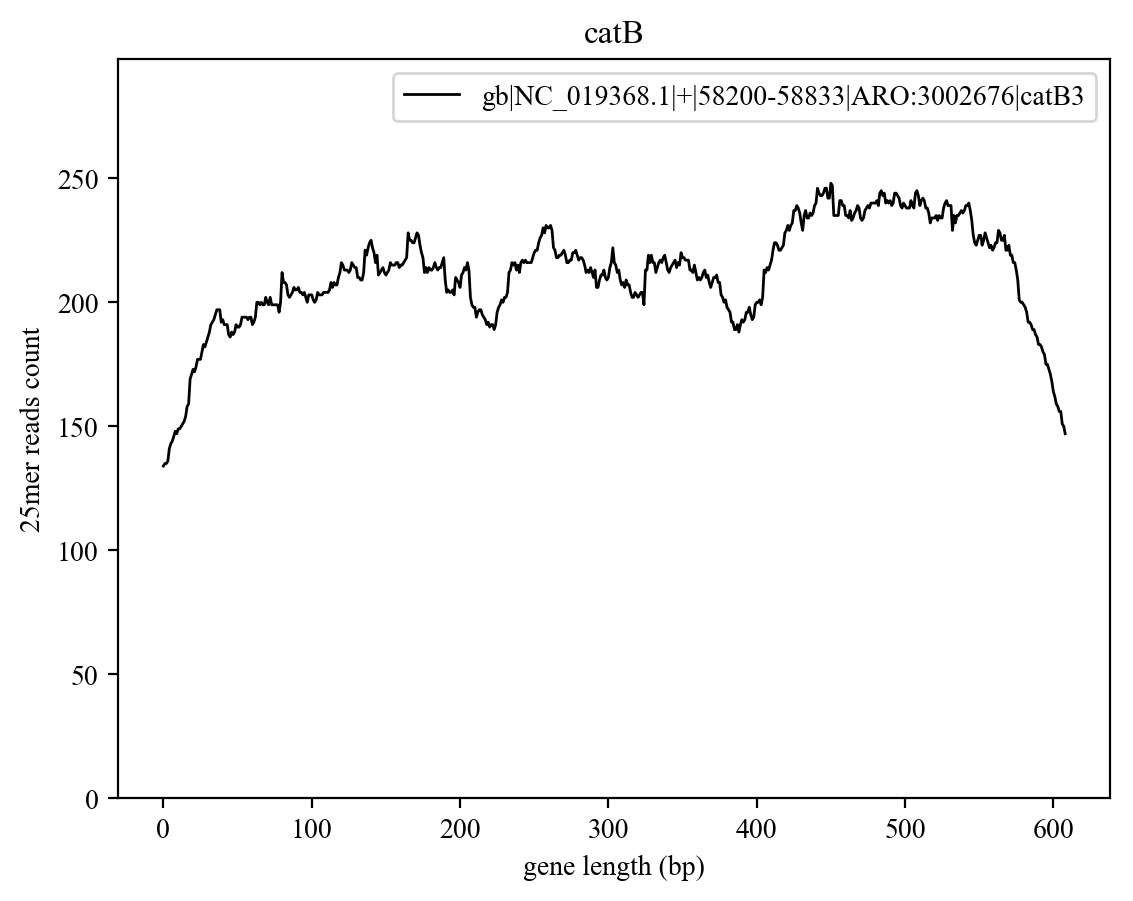

Supplement: Supplementary file 2 — Additional file 2. Archive containing files for evaluation k-mer performance and scoring generated by the k-mer method. [file 12859_2019_3335_MOESM2_ESM.zip › kmer/SJTUF10231_Typhimurium/ar_nucl_25/catB_25mer.png]

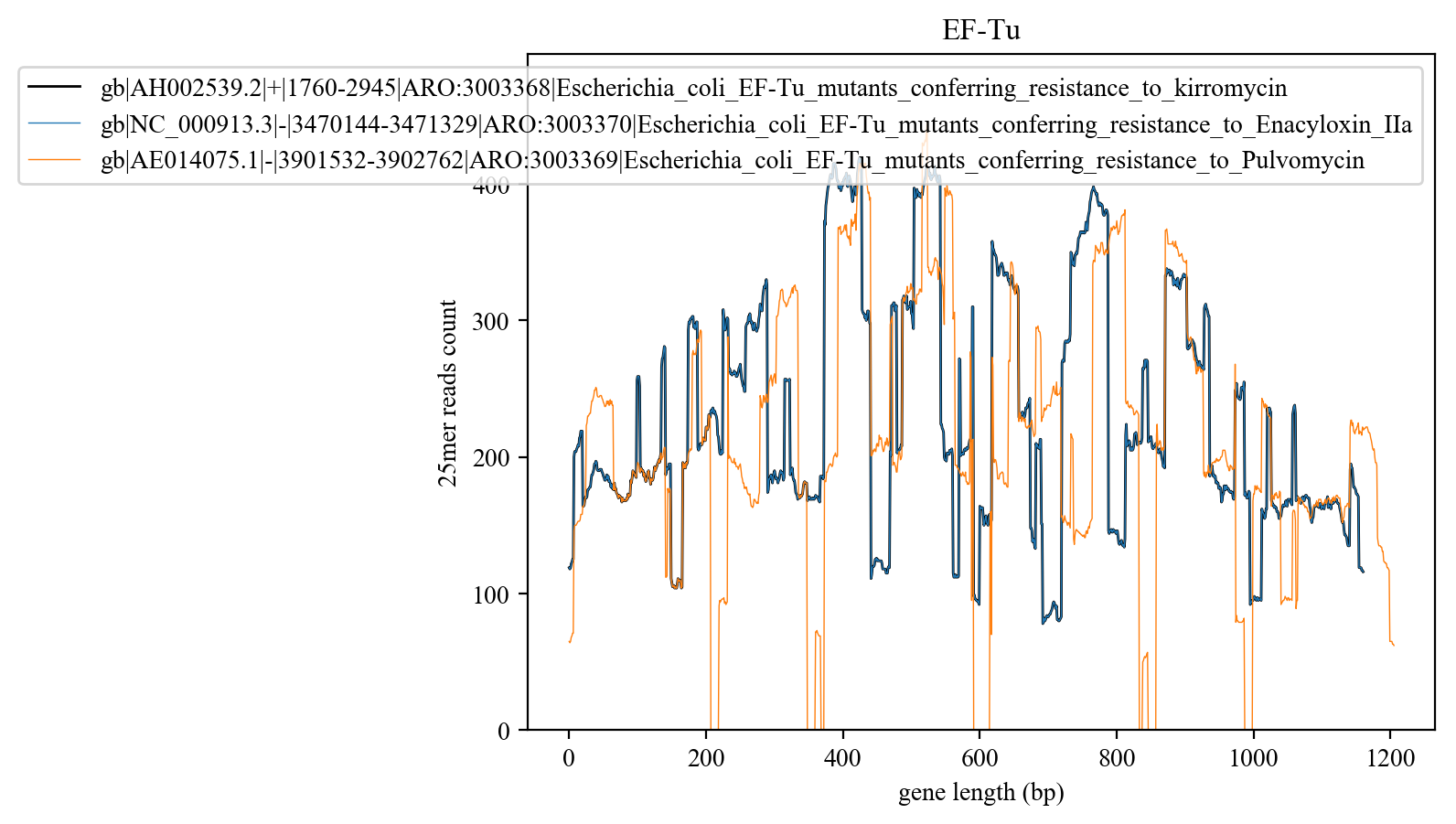

Supplement: Supplementary file 2 — Additional file 2. Archive containing files for evaluation k-mer performance and scoring generated by the k-mer method. [file 12859_2019_3335_MOESM2_ESM.zip › kmer/SJTUF10231_Typhimurium/ar_nucl_25/EF-Tu_25mer.png]

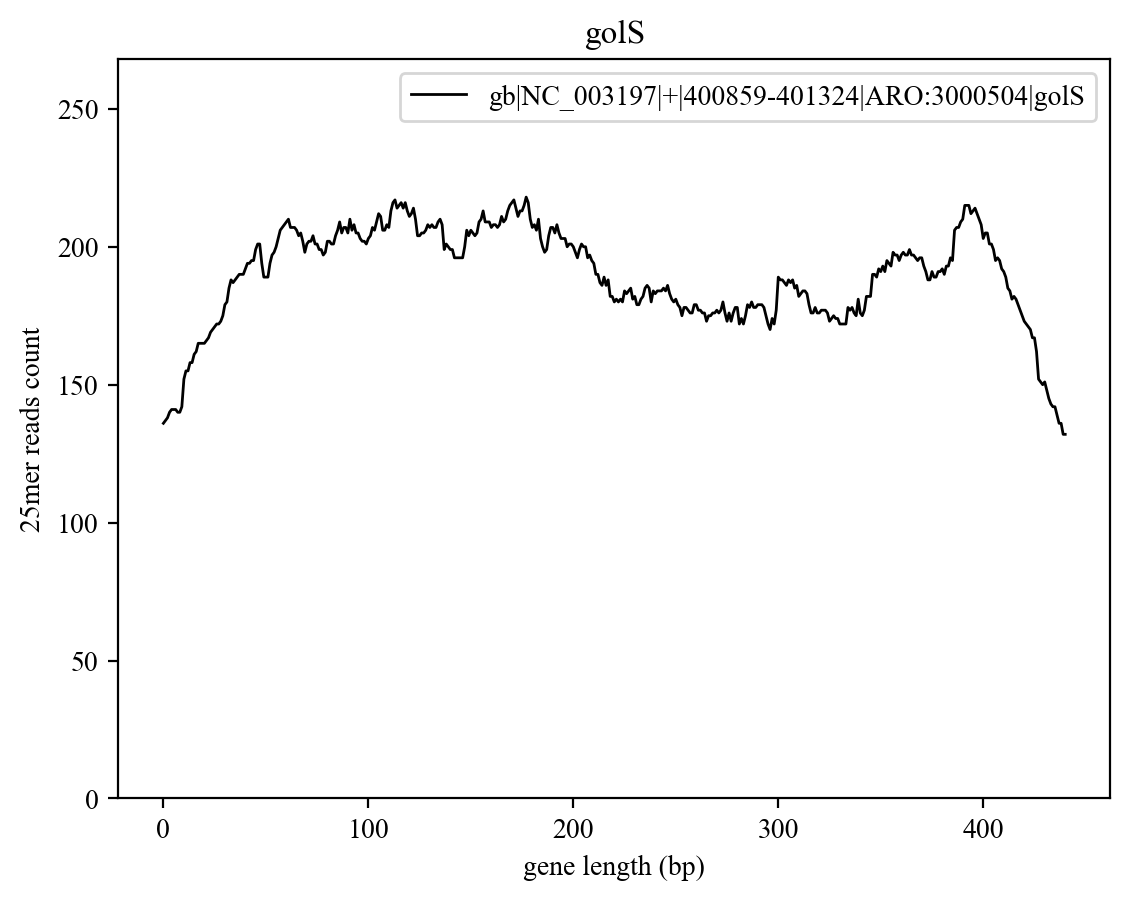

Supplement: Supplementary file 2 — Additional file 2. Archive containing files for evaluation k-mer performance and scoring generated by the k-mer method. [file 12859_2019_3335_MOESM2_ESM.zip › kmer/SJTUF10231_Typhimurium/ar_nucl_25/golS_25mer.png]

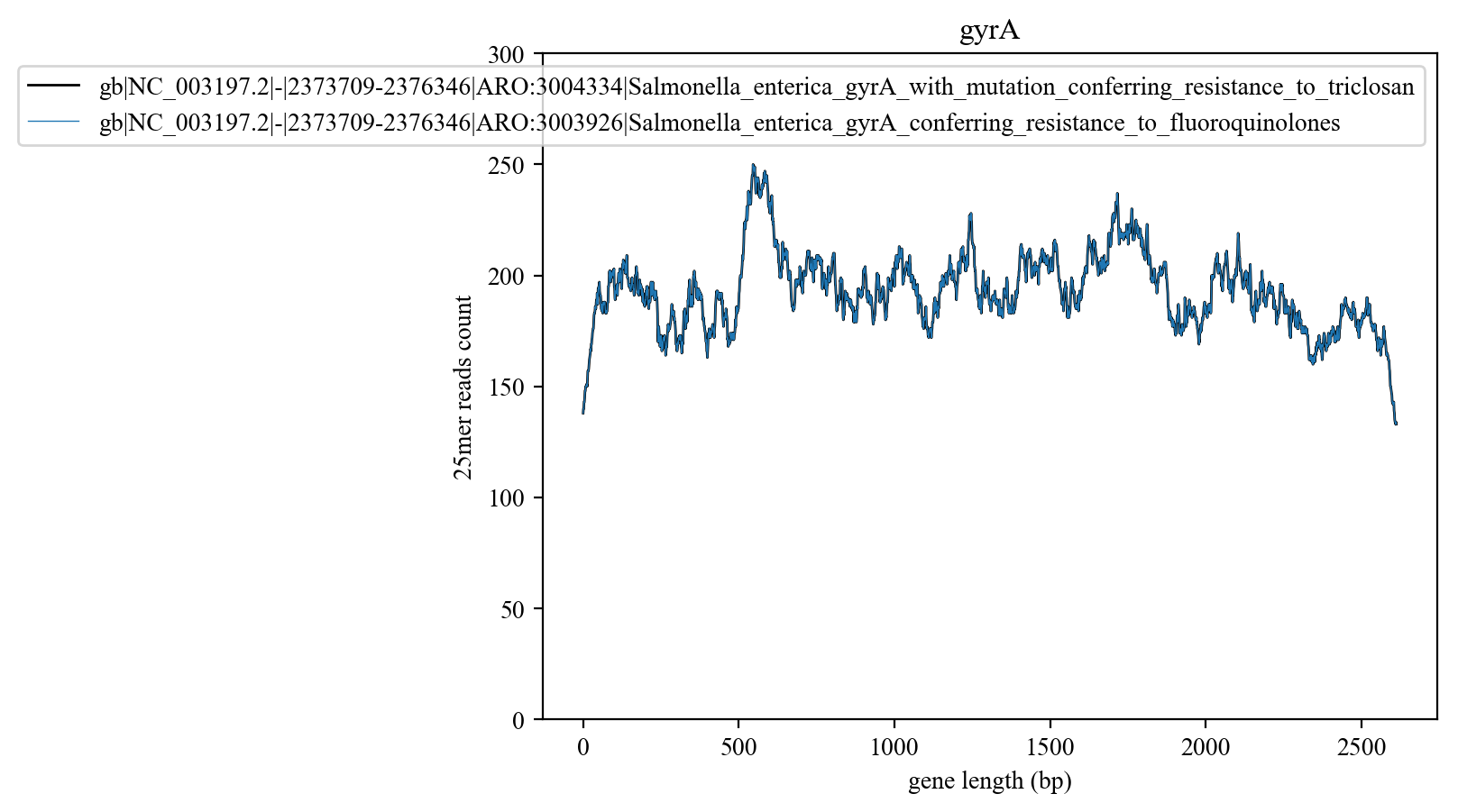

Supplement: Supplementary file 2 — Additional file 2. Archive containing files for evaluation k-mer performance and scoring generated by the k-mer method. [file 12859_2019_3335_MOESM2_ESM.zip › kmer/SJTUF10231_Typhimurium/ar_nucl_25/gyrA_25mer.png]

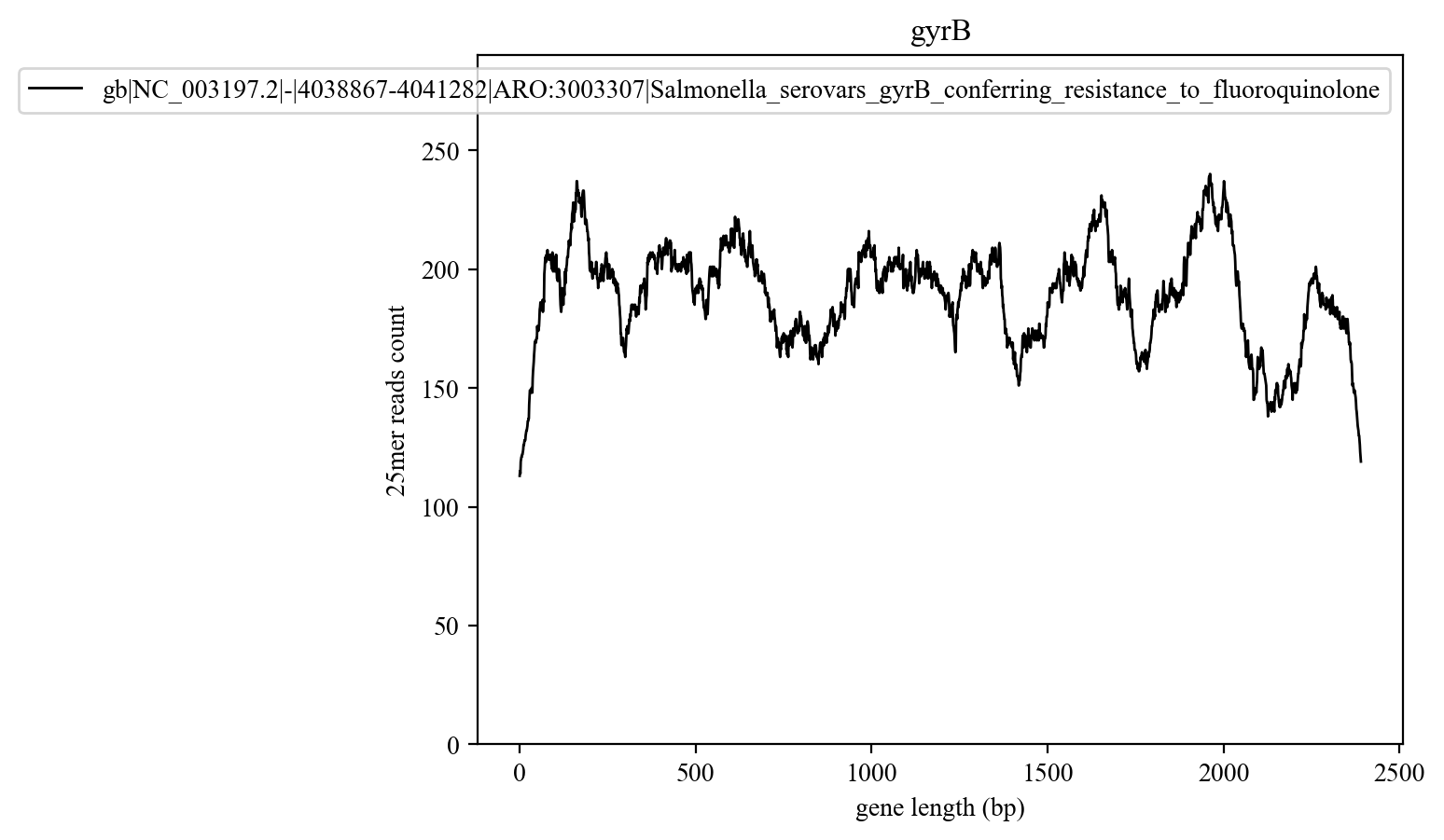

Supplement: Supplementary file 2 — Additional file 2. Archive containing files for evaluation k-mer performance and scoring generated by the k-mer method. [file 12859_2019_3335_MOESM2_ESM.zip › kmer/SJTUF10231_Typhimurium/ar_nucl_25/gyrB_25mer.png]

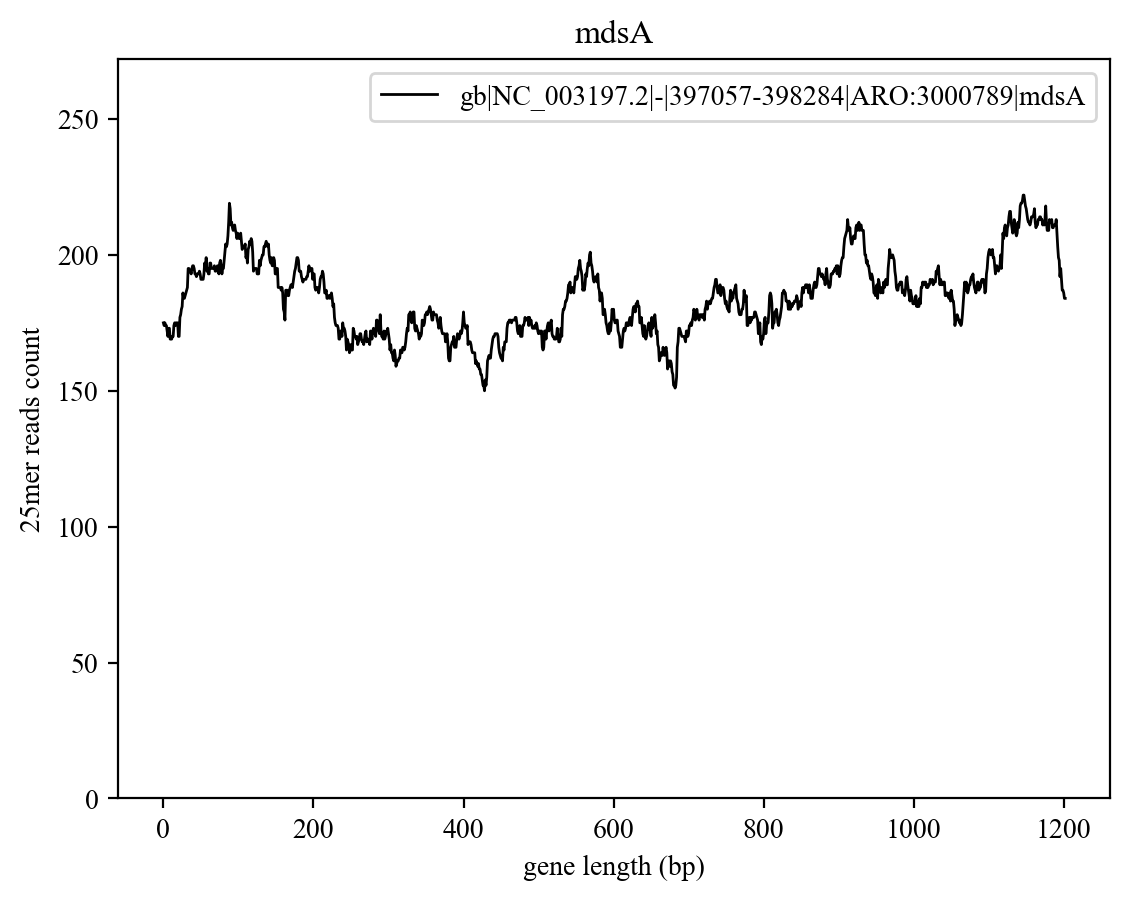

Supplement: Supplementary file 2 — Additional file 2. Archive containing files for evaluation k-mer performance and scoring generated by the k-mer method. [file 12859_2019_3335_MOESM2_ESM.zip › kmer/SJTUF10231_Typhimurium/ar_nucl_25/mdsA_25mer.png]

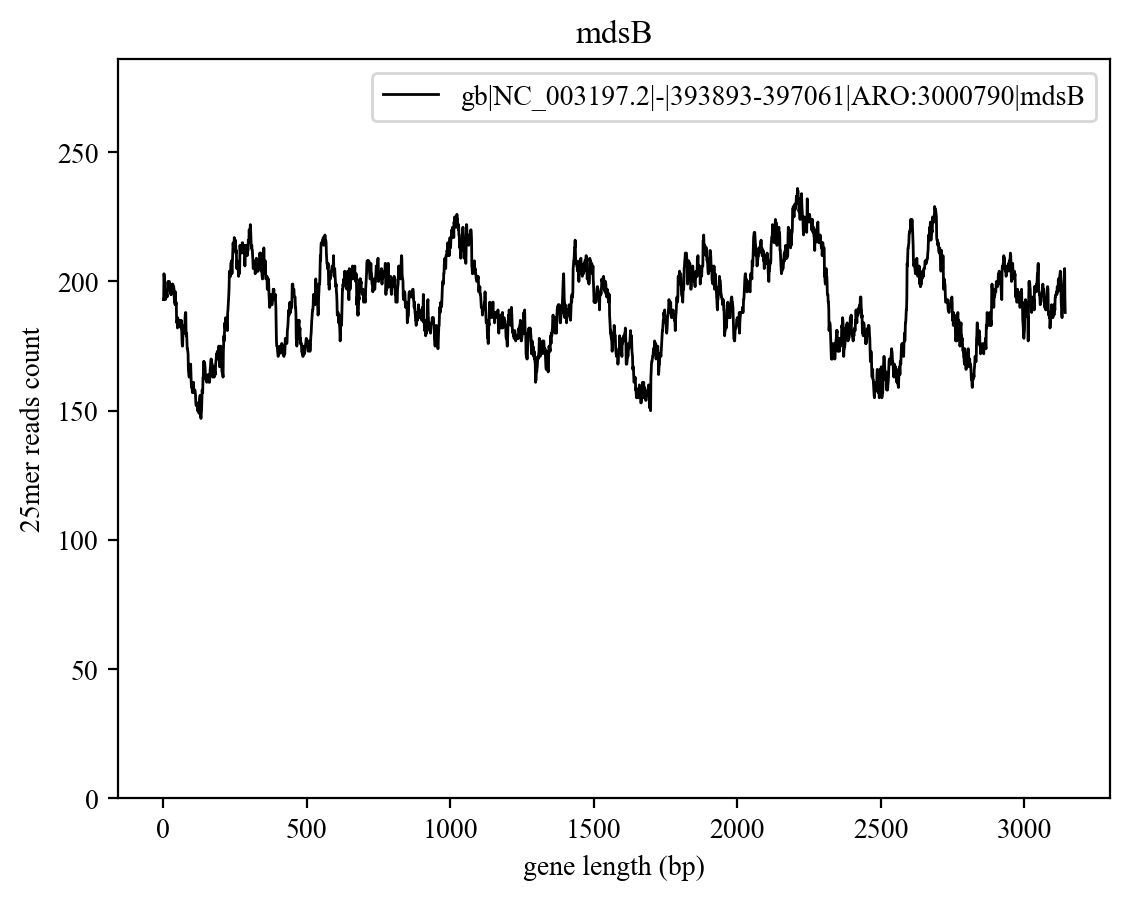

Supplement: Supplementary file 2 — Additional file 2. Archive containing files for evaluation k-mer performance and scoring generated by the k-mer method. [file 12859_2019_3335_MOESM2_ESM.zip › kmer/SJTUF10231_Typhimurium/ar_nucl_25/mdsB_25mer.png]

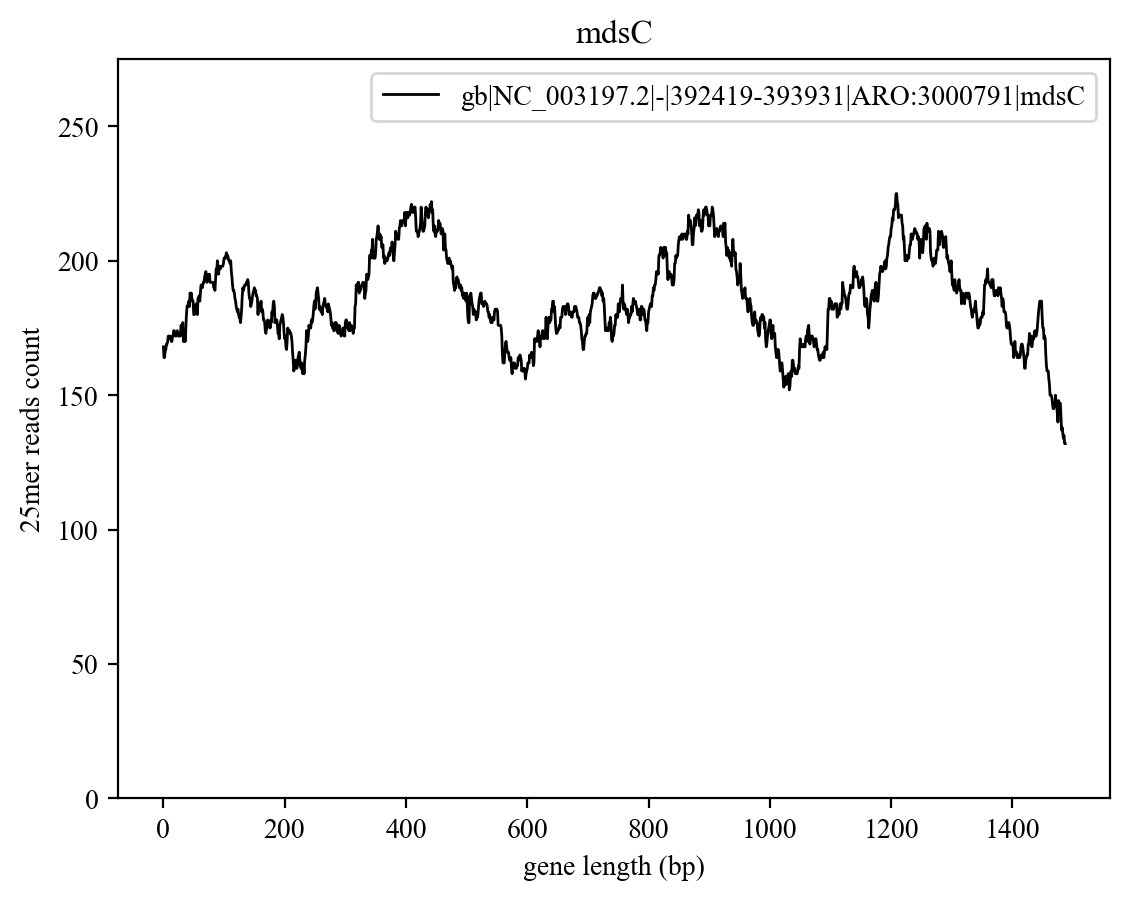

Supplement: Supplementary file 2 — Additional file 2. Archive containing files for evaluation k-mer performance and scoring generated by the k-mer method. [file 12859_2019_3335_MOESM2_ESM.zip › kmer/SJTUF10231_Typhimurium/ar_nucl_25/mdsC_25mer.png]

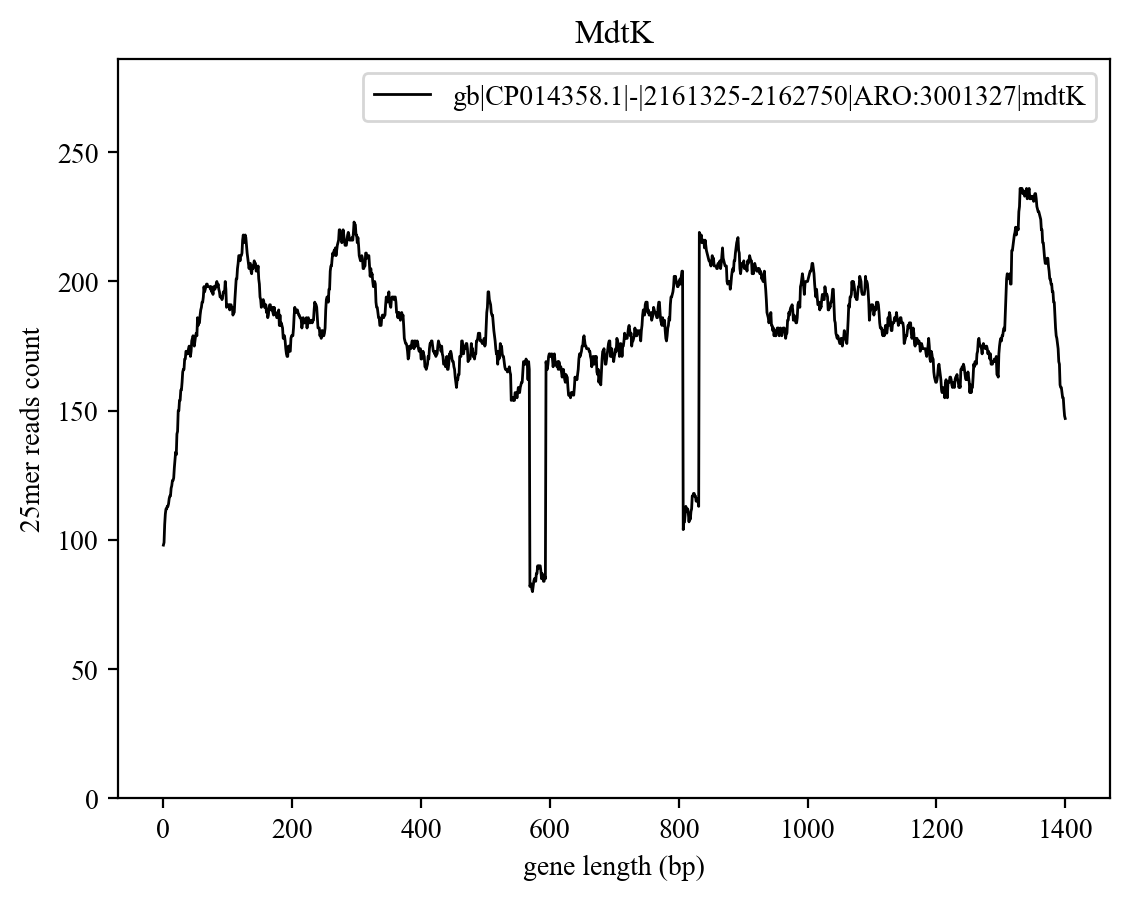

Supplement: Supplementary file 2 — Additional file 2. Archive containing files for evaluation k-mer performance and scoring generated by the k-mer method. [file 12859_2019_3335_MOESM2_ESM.zip › kmer/SJTUF10231_Typhimurium/ar_nucl_25/MdtK_25mer.png]

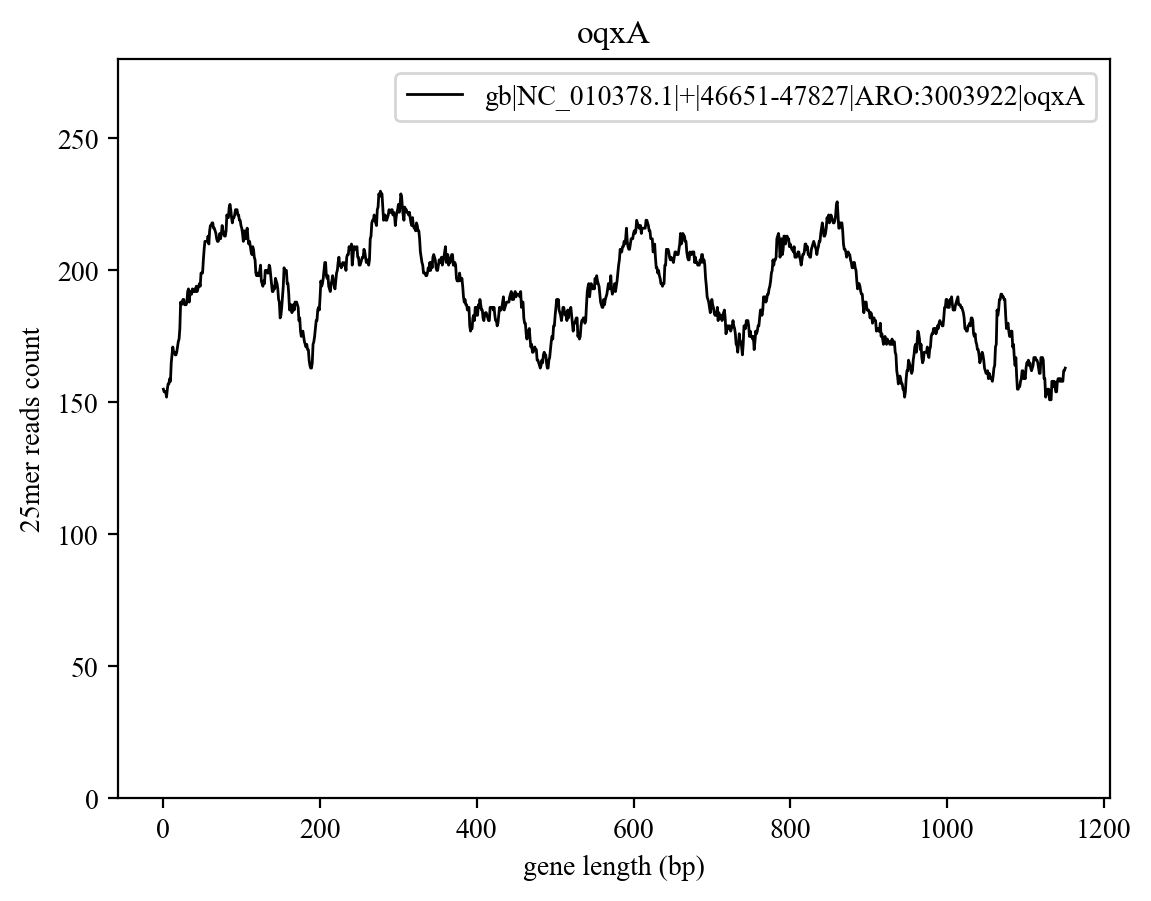

Supplement: Supplementary file 2 — Additional file 2. Archive containing files for evaluation k-mer performance and scoring generated by the k-mer method. [file 12859_2019_3335_MOESM2_ESM.zip › kmer/SJTUF10231_Typhimurium/ar_nucl_25/oqxA_25mer.png]

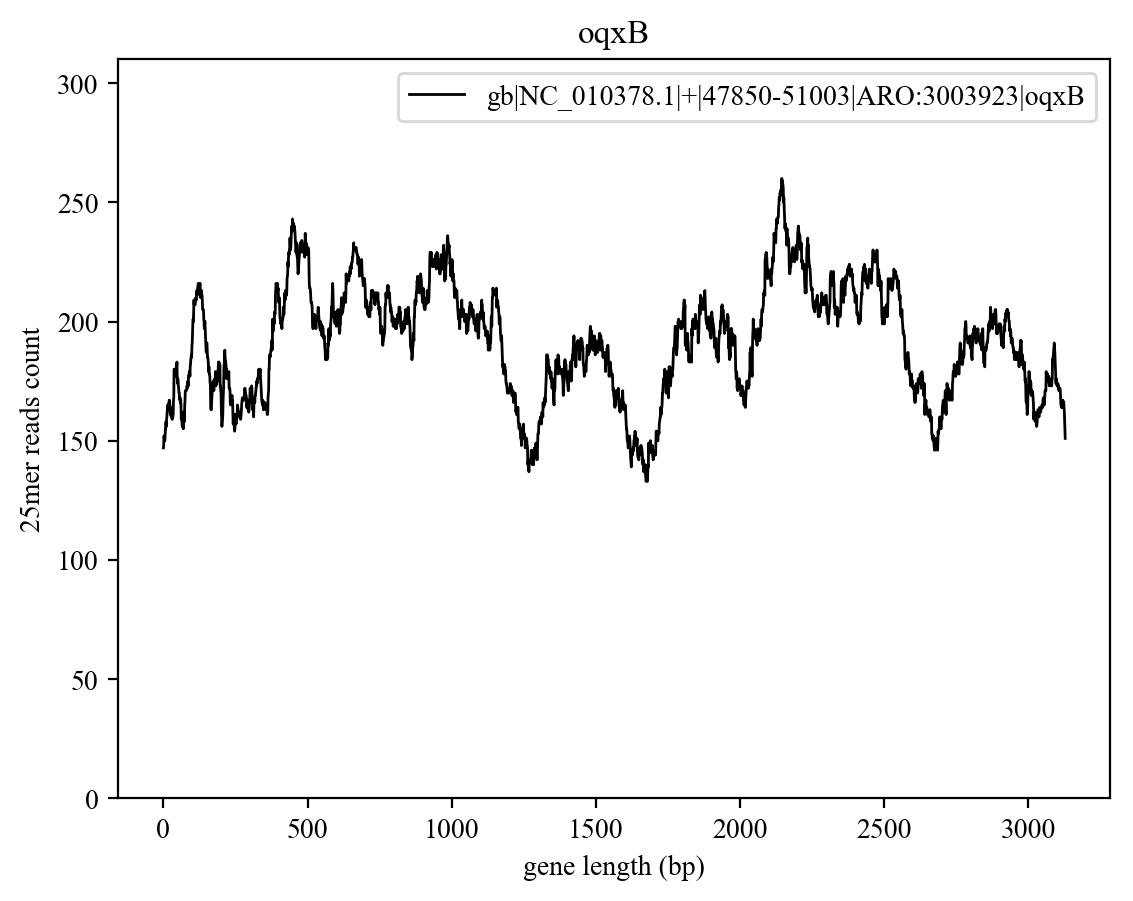

Supplement: Supplementary file 2 — Additional file 2. Archive containing files for evaluation k-mer performance and scoring generated by the k-mer method. [file 12859_2019_3335_MOESM2_ESM.zip › kmer/SJTUF10231_Typhimurium/ar_nucl_25/oqxB_25mer.png]

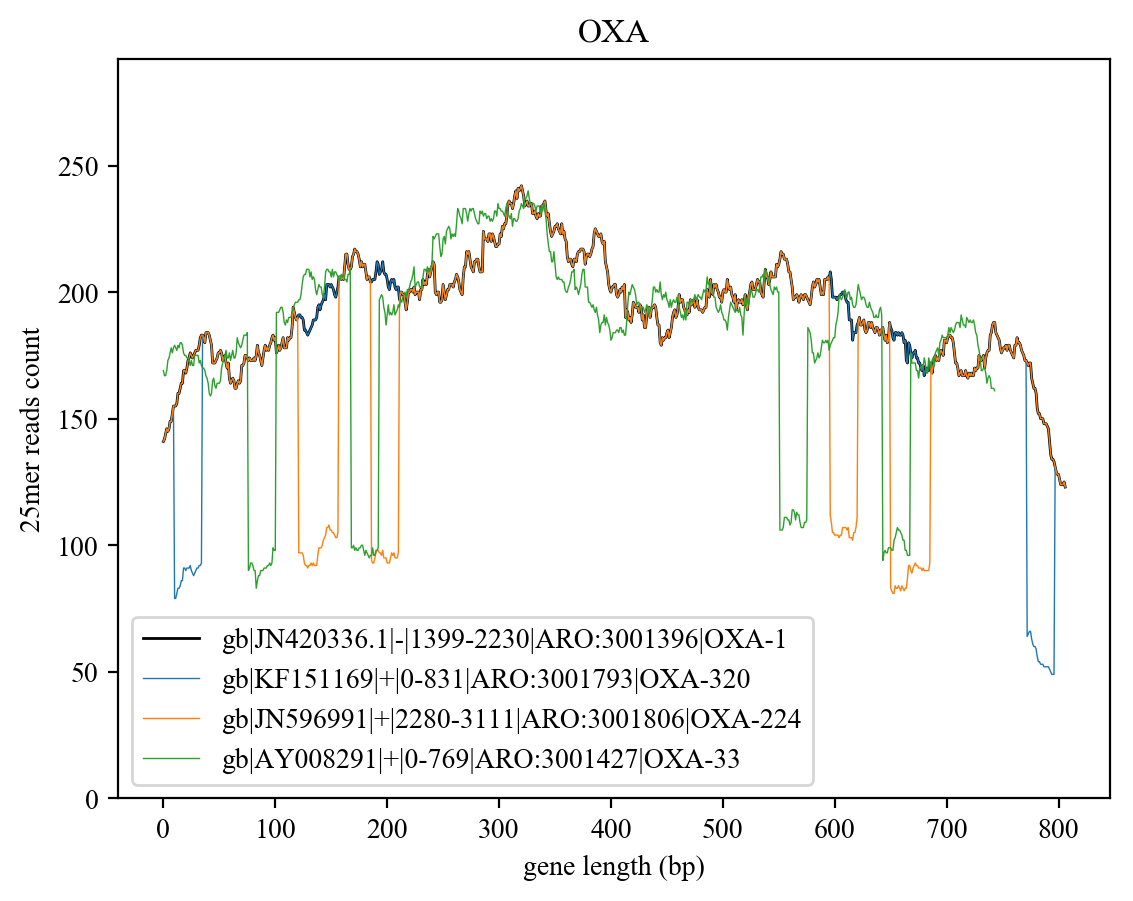

Supplement: Supplementary file 2 — Additional file 2. Archive containing files for evaluation k-mer performance and scoring generated by the k-mer method. [file 12859_2019_3335_MOESM2_ESM.zip › kmer/SJTUF10231_Typhimurium/ar_nucl_25/OXA_25mer.png]

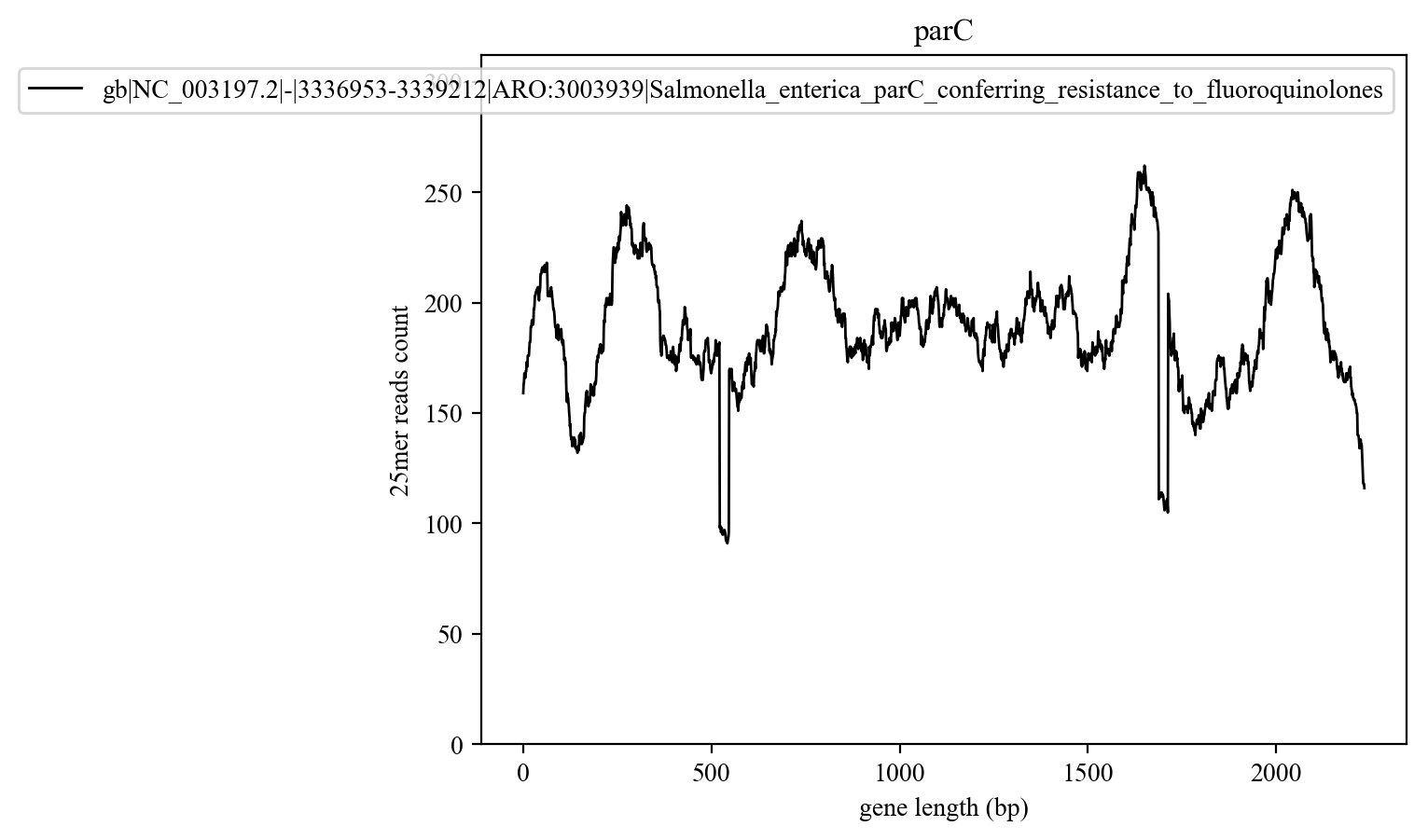

Supplement: Supplementary file 2 — Additional file 2. Archive containing files for evaluation k-mer performance and scoring generated by the k-mer method. [file 12859_2019_3335_MOESM2_ESM.zip › kmer/SJTUF10231_Typhimurium/ar_nucl_25/parC_25mer.png]

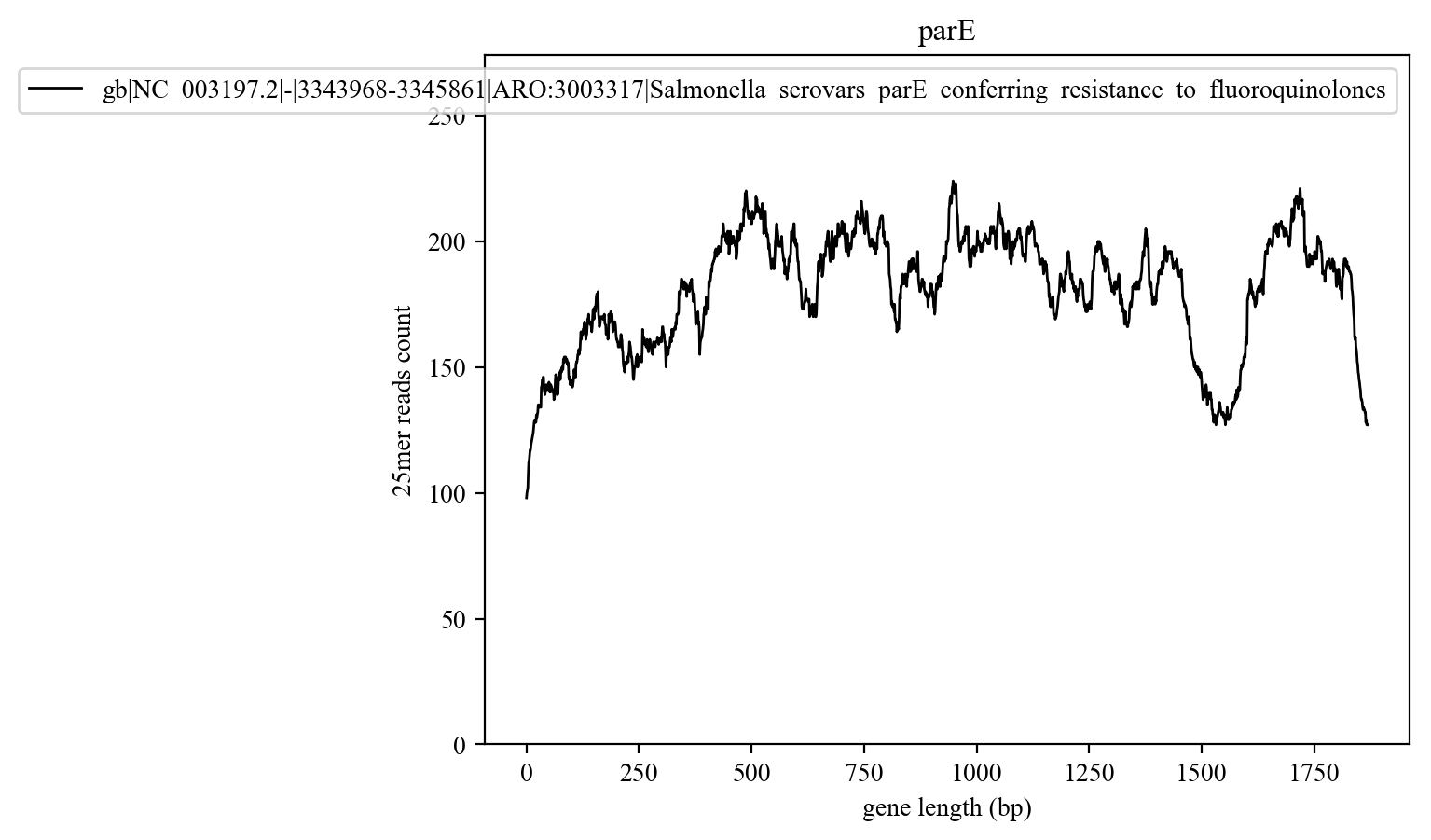

Supplement: Supplementary file 2 — Additional file 2. Archive containing files for evaluation k-mer performance and scoring generated by the k-mer method. [file 12859_2019_3335_MOESM2_ESM.zip › kmer/SJTUF10231_Typhimurium/ar_nucl_25/parE_25mer.png]

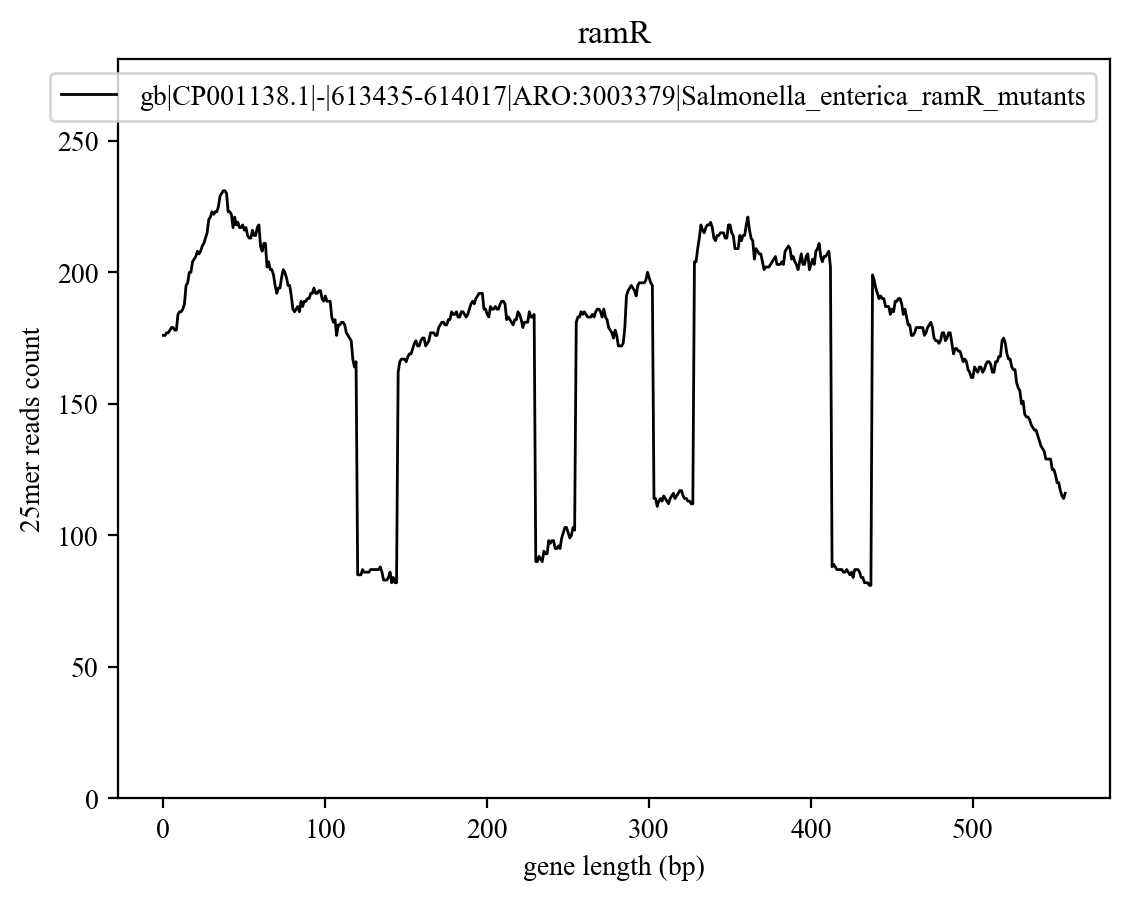

Supplement: Supplementary file 2 — Additional file 2. Archive containing files for evaluation k-mer performance and scoring generated by the k-mer method. [file 12859_2019_3335_MOESM2_ESM.zip › kmer/SJTUF10231_Typhimurium/ar_nucl_25/ramR_25mer.png]

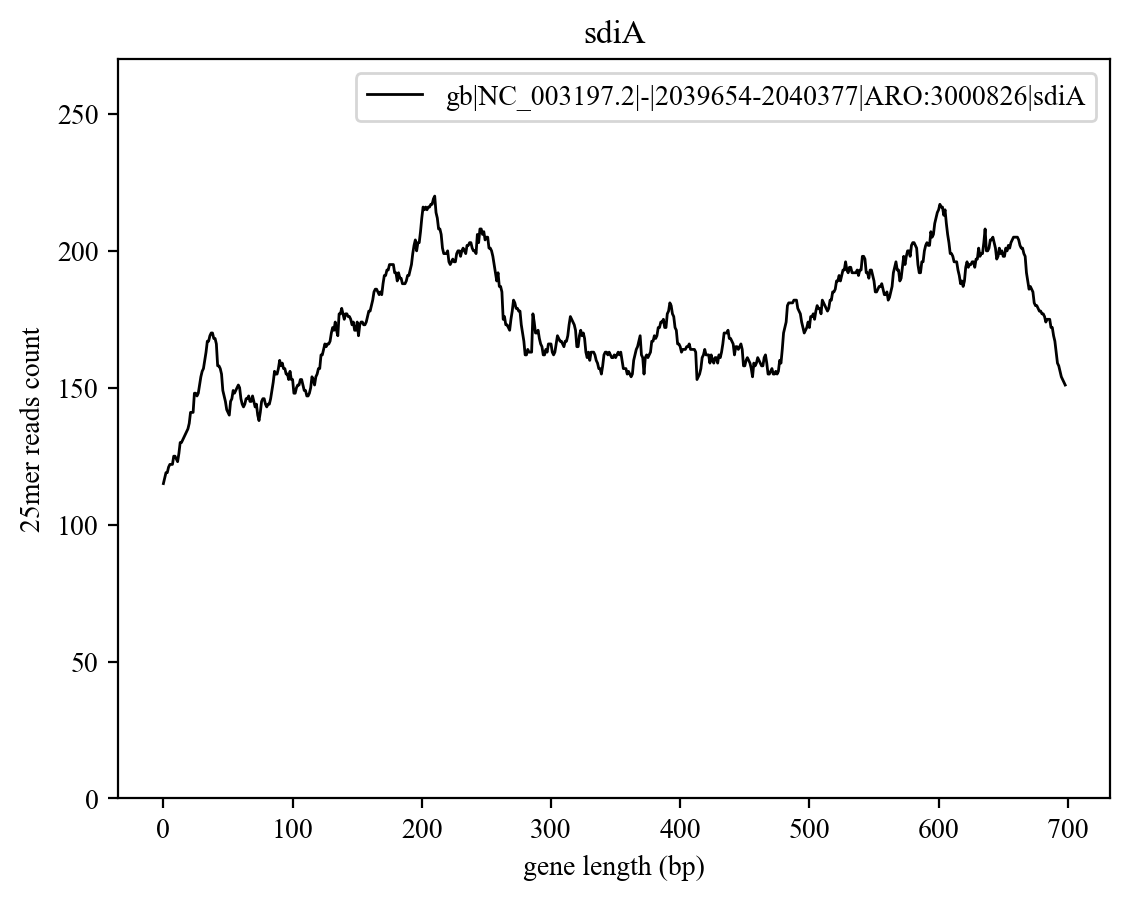

Supplement: Supplementary file 2 — Additional file 2. Archive containing files for evaluation k-mer performance and scoring generated by the k-mer method. [file 12859_2019_3335_MOESM2_ESM.zip › kmer/SJTUF10231_Typhimurium/ar_nucl_25/sdiA_25mer.png]

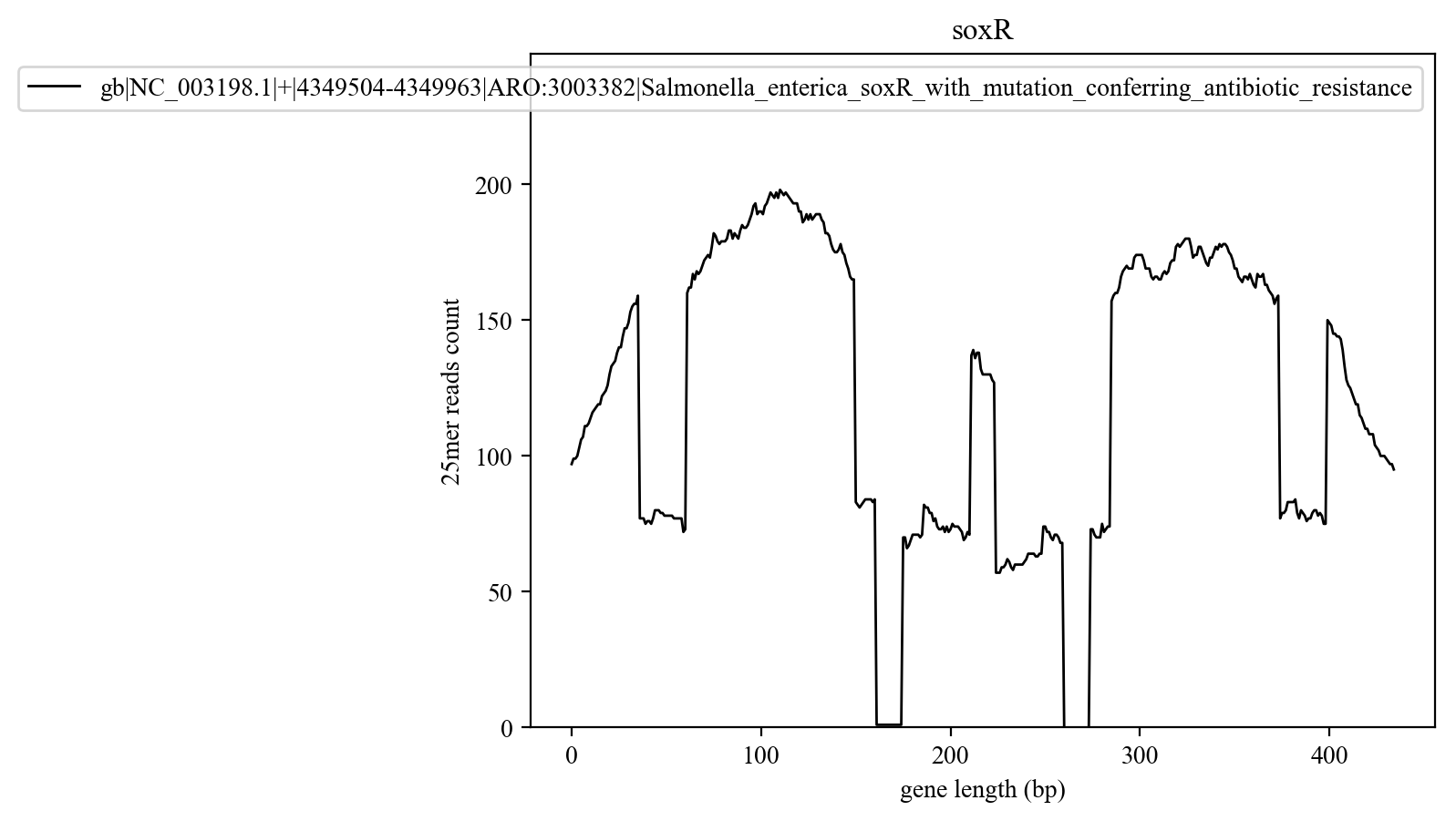

Supplement: Supplementary file 2 — Additional file 2. Archive containing files for evaluation k-mer performance and scoring generated by the k-mer method. [file 12859_2019_3335_MOESM2_ESM.zip › kmer/SJTUF10231_Typhimurium/ar_nucl_25/soxR_25mer.png]

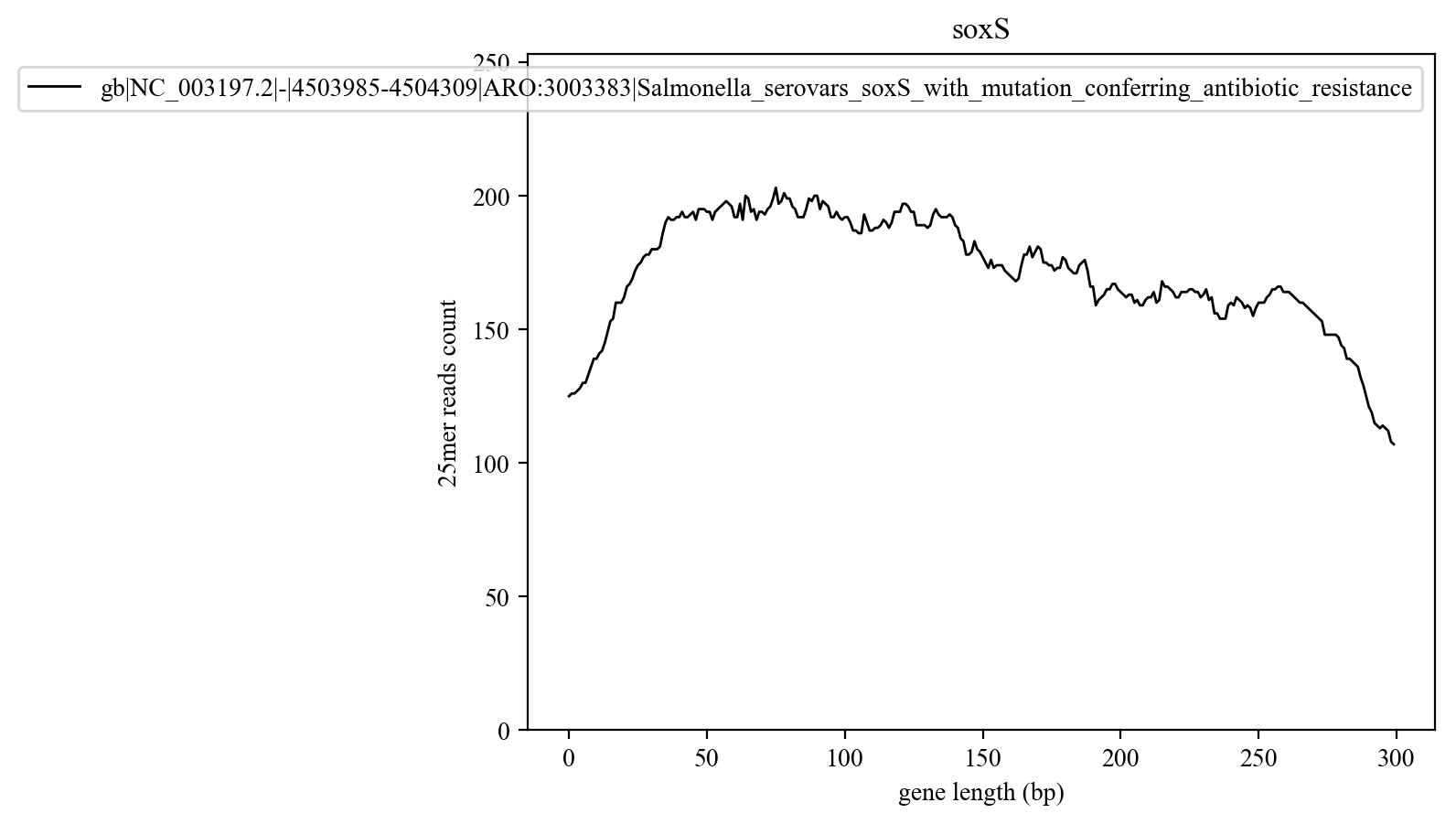

Supplement: Supplementary file 2 — Additional file 2. Archive containing files for evaluation k-mer performance and scoring generated by the k-mer method. [file 12859_2019_3335_MOESM2_ESM.zip › kmer/SJTUF10231_Typhimurium/ar_nucl_25/soxS_25mer.png]

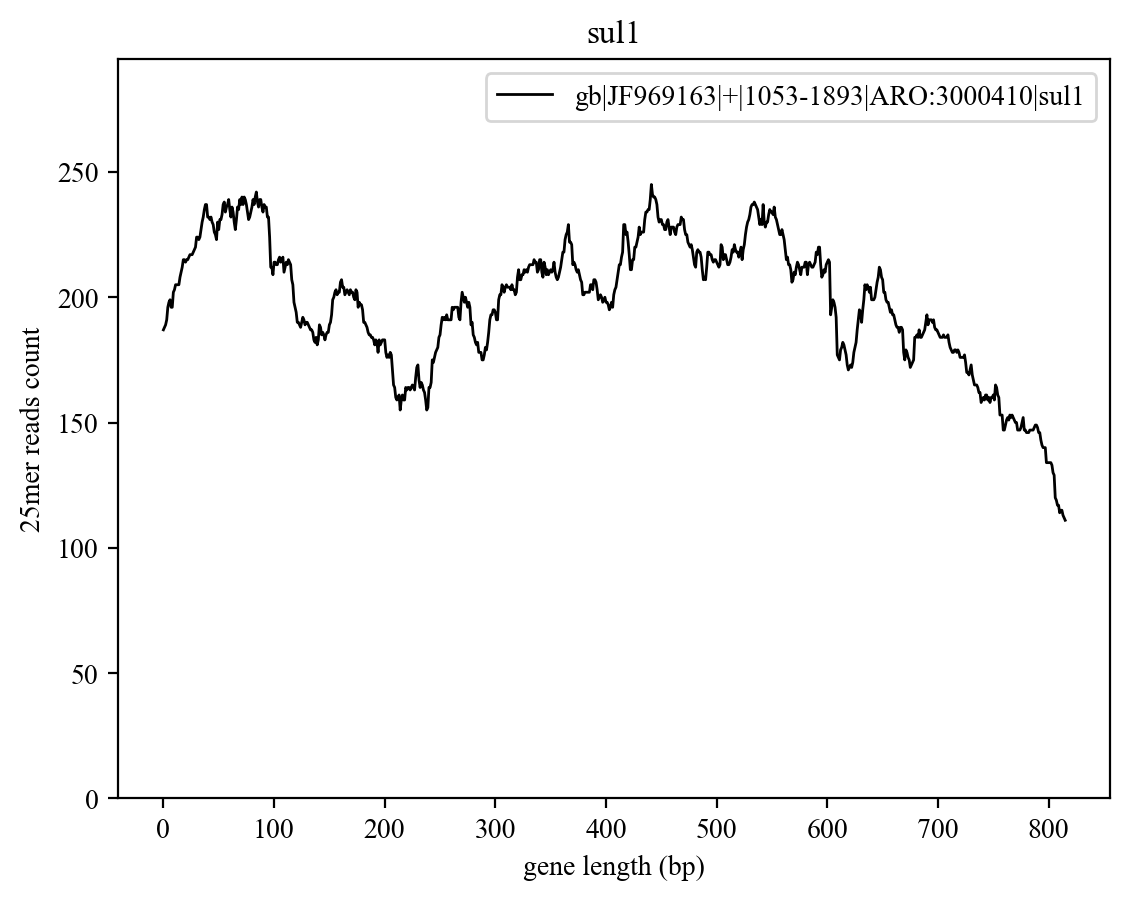

Supplement: Supplementary file 2 — Additional file 2. Archive containing files for evaluation k-mer performance and scoring generated by the k-mer method. [file 12859_2019_3335_MOESM2_ESM.zip › kmer/SJTUF10231_Typhimurium/ar_nucl_25/sul1_25mer.png]

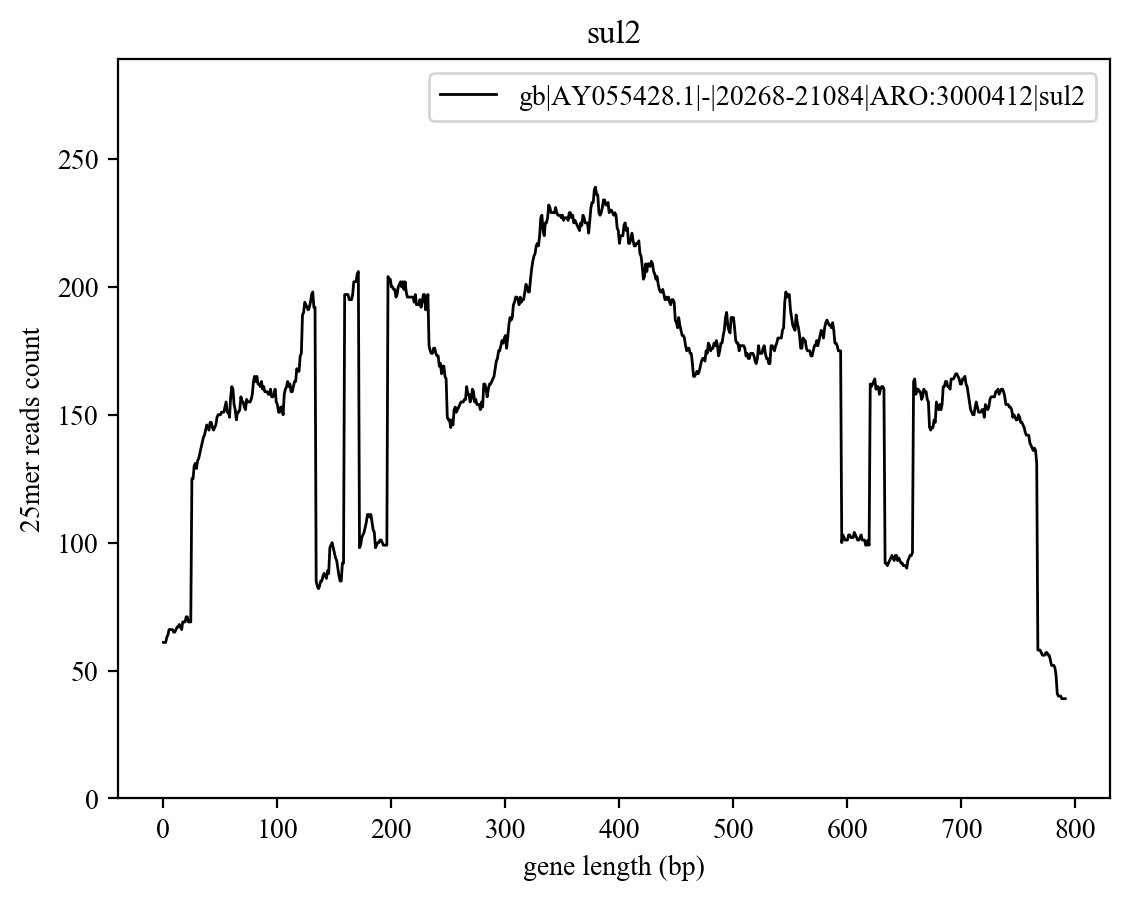

Supplement: Supplementary file 2 — Additional file 2. Archive containing files for evaluation k-mer performance and scoring generated by the k-mer method. [file 12859_2019_3335_MOESM2_ESM.zip › kmer/SJTUF10231_Typhimurium/ar_nucl_25/sul2_25mer.png]

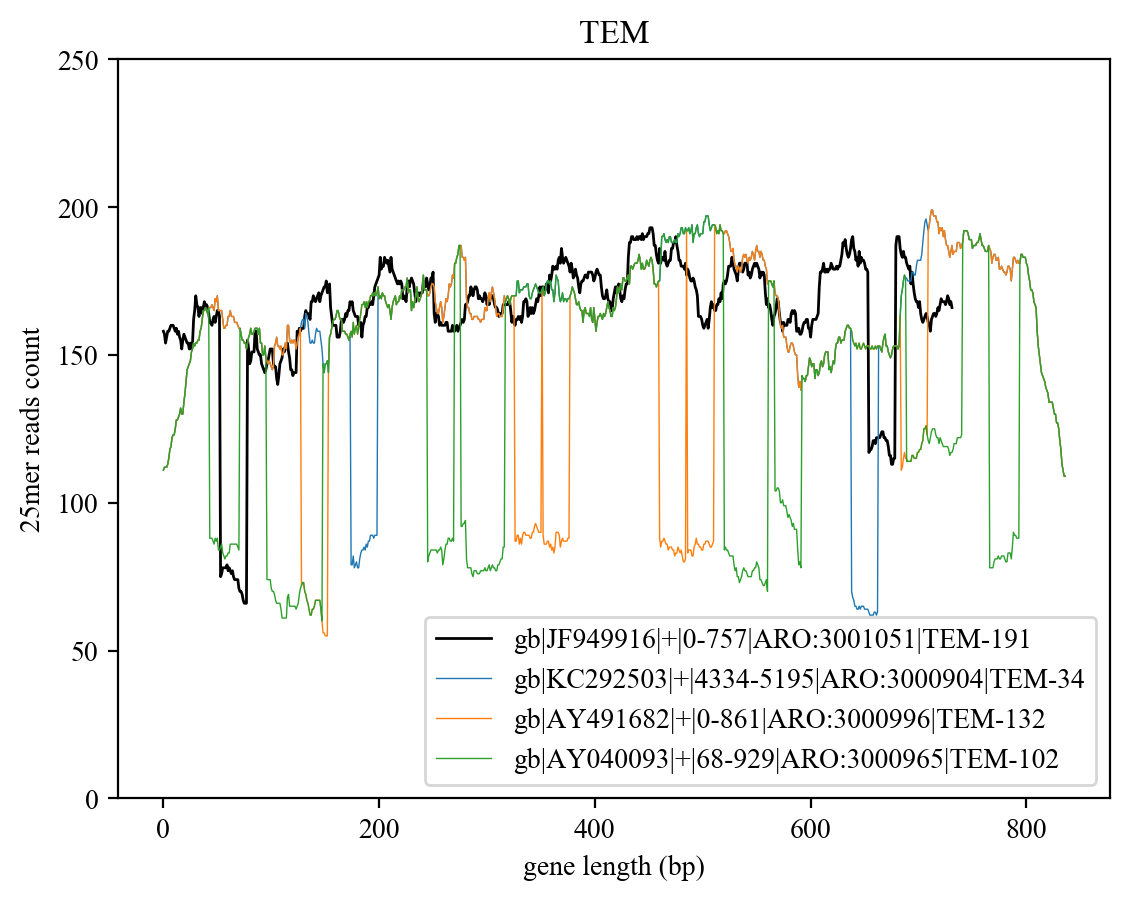

Supplement: Supplementary file 2 — Additional file 2. Archive containing files for evaluation k-mer performance and scoring generated by the k-mer method. [file 12859_2019_3335_MOESM2_ESM.zip › kmer/SJTUF10231_Typhimurium/ar_nucl_25/TEM_25mer.png]

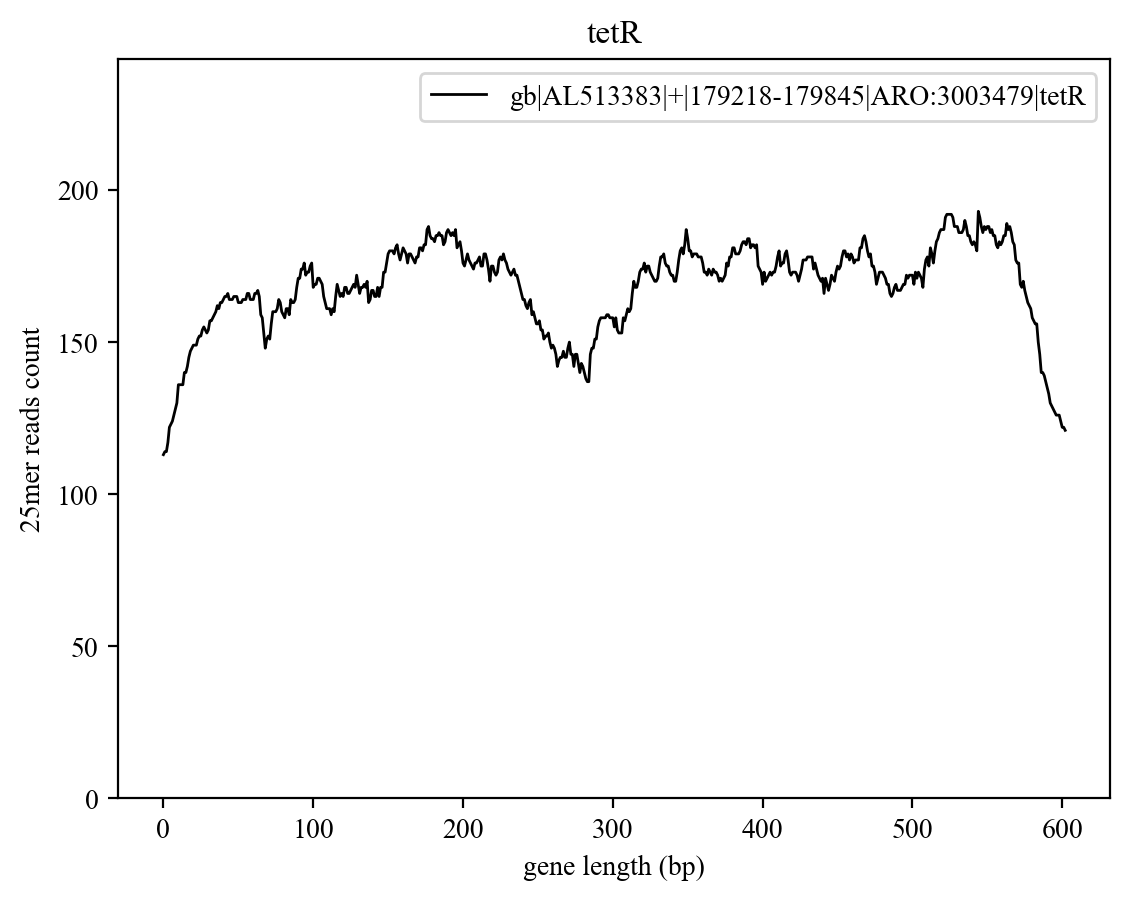

Supplement: Supplementary file 2 — Additional file 2. Archive containing files for evaluation k-mer performance and scoring generated by the k-mer method. [file 12859_2019_3335_MOESM2_ESM.zip › kmer/SJTUF10231_Typhimurium/ar_nucl_25/tetR_25mer.png]

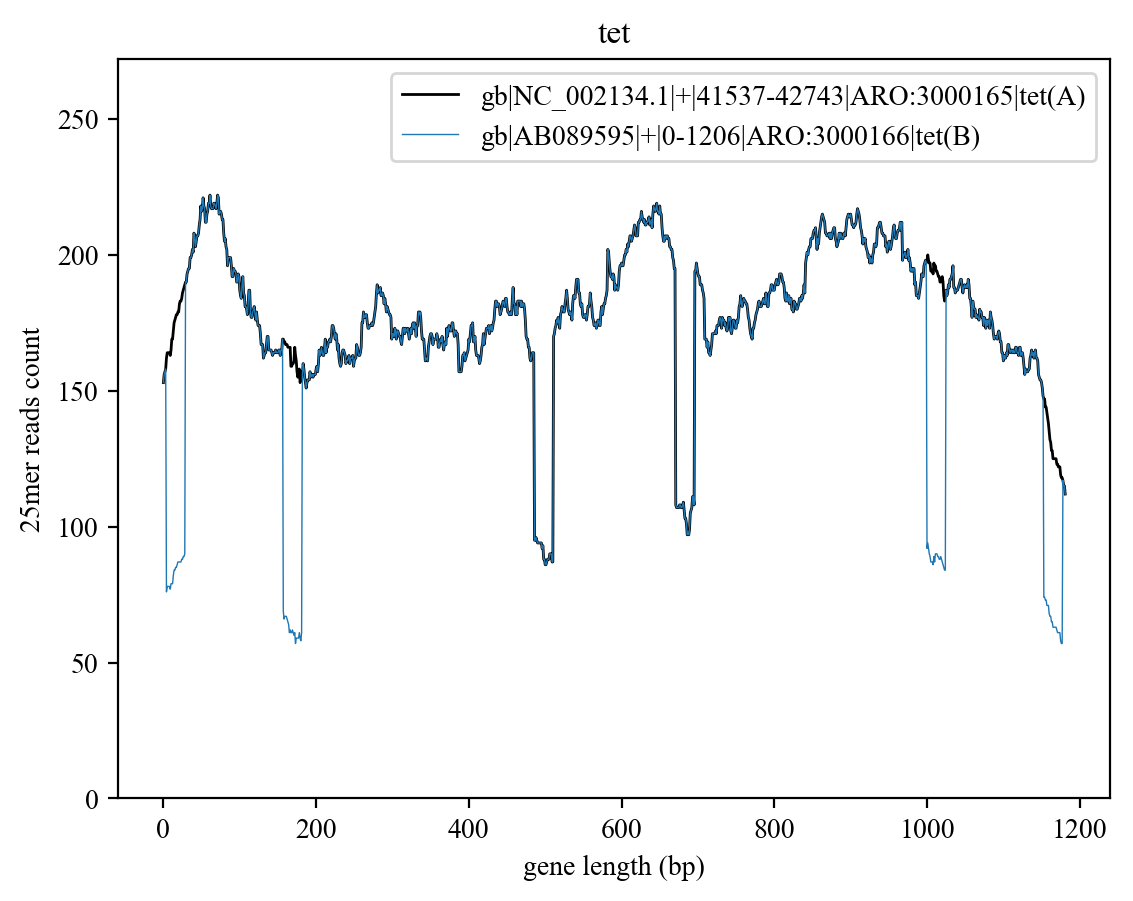

Supplement: Supplementary file 2 — Additional file 2. Archive containing files for evaluation k-mer performance and scoring generated by the k-mer method. [file 12859_2019_3335_MOESM2_ESM.zip › kmer/SJTUF10231_Typhimurium/ar_nucl_25/tet_25mer.png]

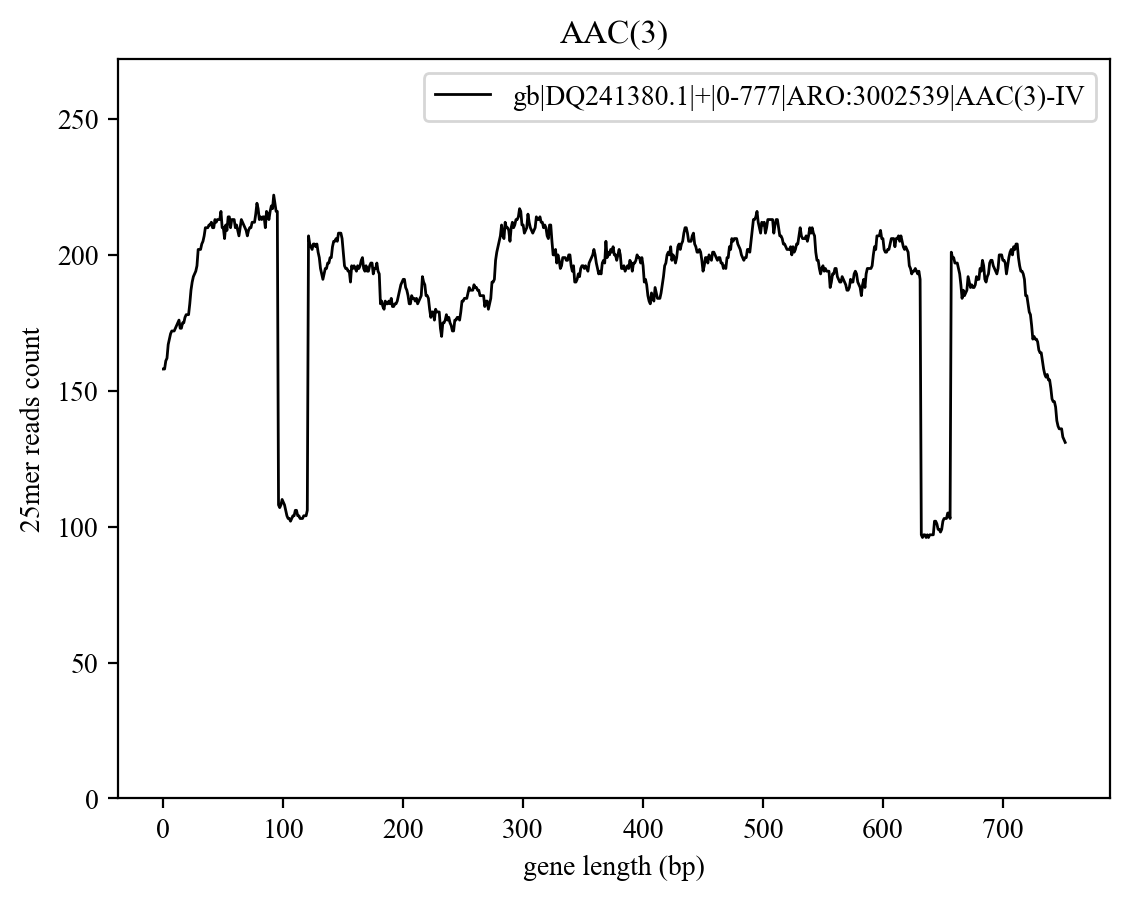

Supplement: Supplementary file 2 — Additional file 2. Archive containing files for evaluation k-mer performance and scoring generated by the k-mer method. [file 12859_2019_3335_MOESM2_ESM.zip › kmer/SJTUF10236_Typhimurium/ar_nucl_25/AAC(3)_25mer.png]

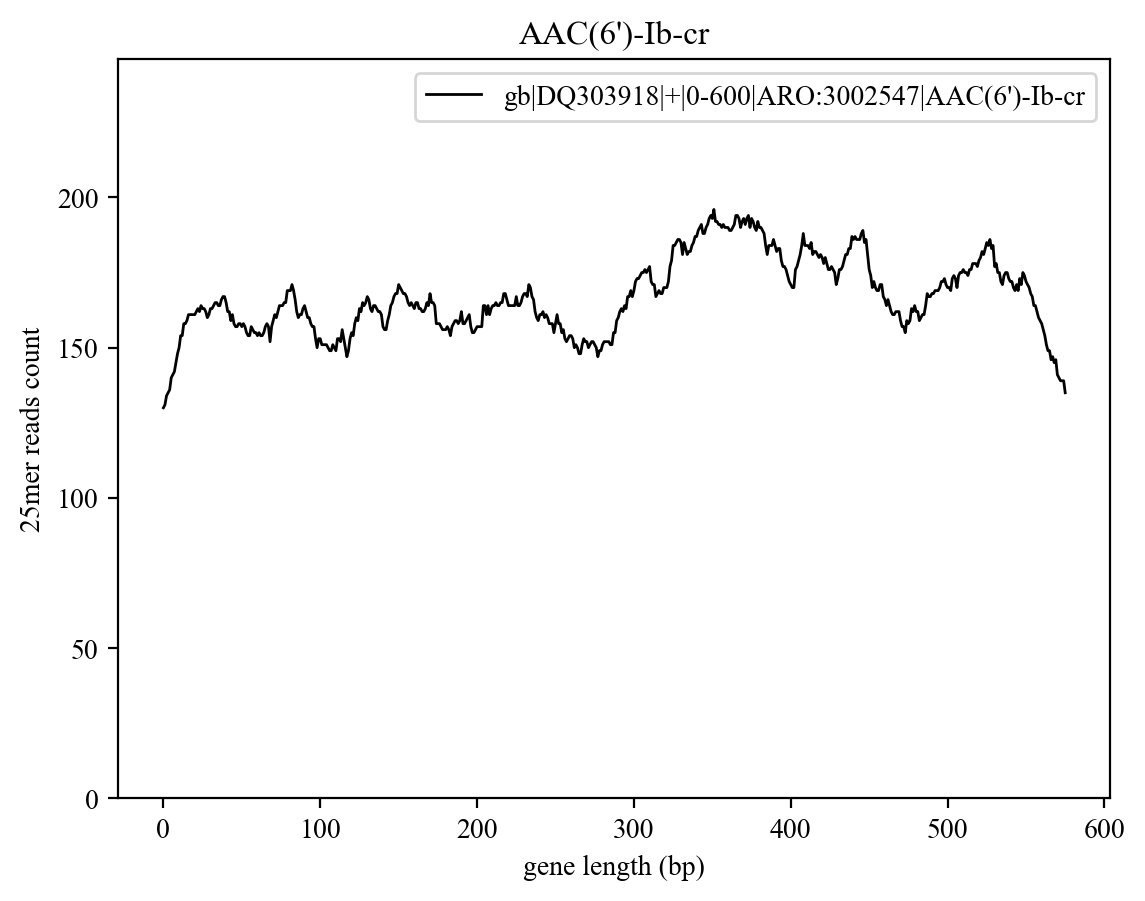

Supplement: Supplementary file 2 — Additional file 2. Archive containing files for evaluation k-mer performance and scoring generated by the k-mer method. [file 12859_2019_3335_MOESM2_ESM.zip › kmer/SJTUF10236_Typhimurium/ar_nucl_25/AAC(6')-Ib-cr_25mer.png]

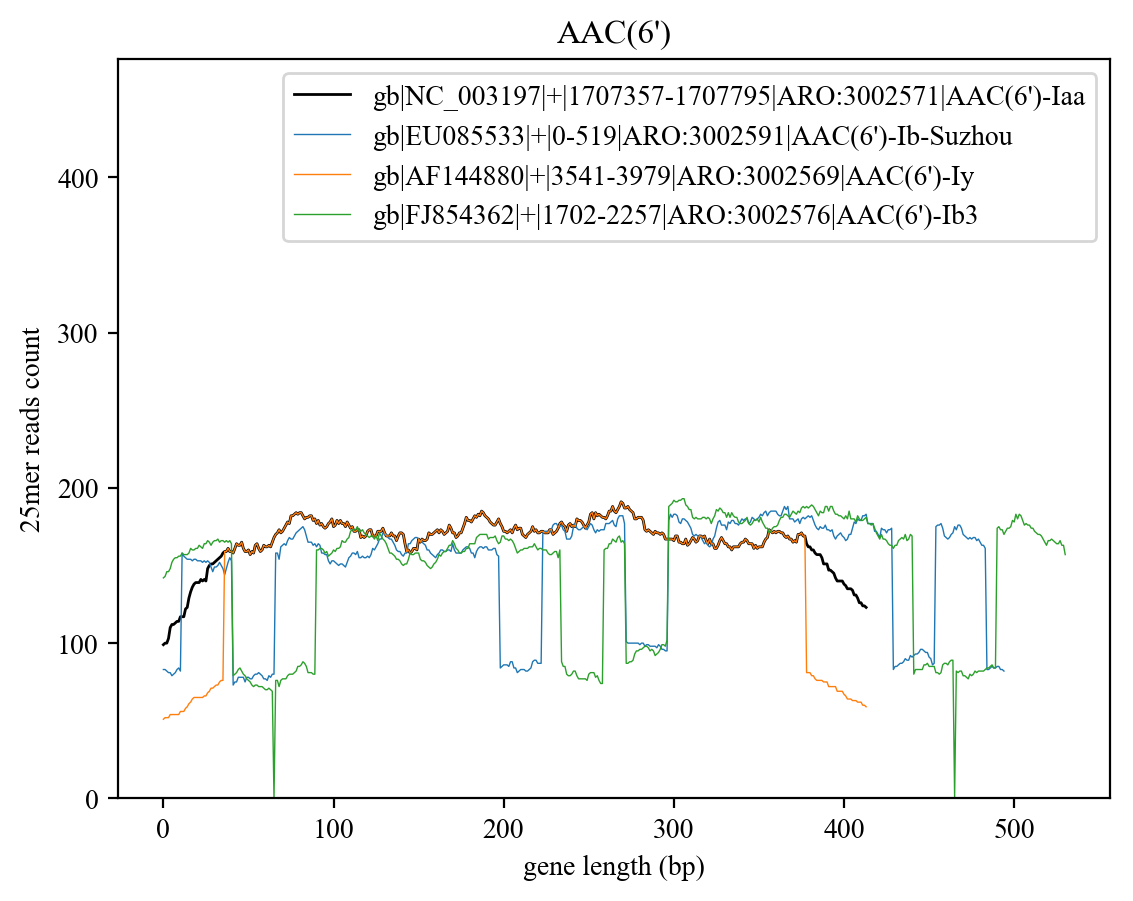

Supplement: Supplementary file 2 — Additional file 2. Archive containing files for evaluation k-mer performance and scoring generated by the k-mer method. [file 12859_2019_3335_MOESM2_ESM.zip › kmer/SJTUF10236_Typhimurium/ar_nucl_25/AAC(6')_25mer.png]

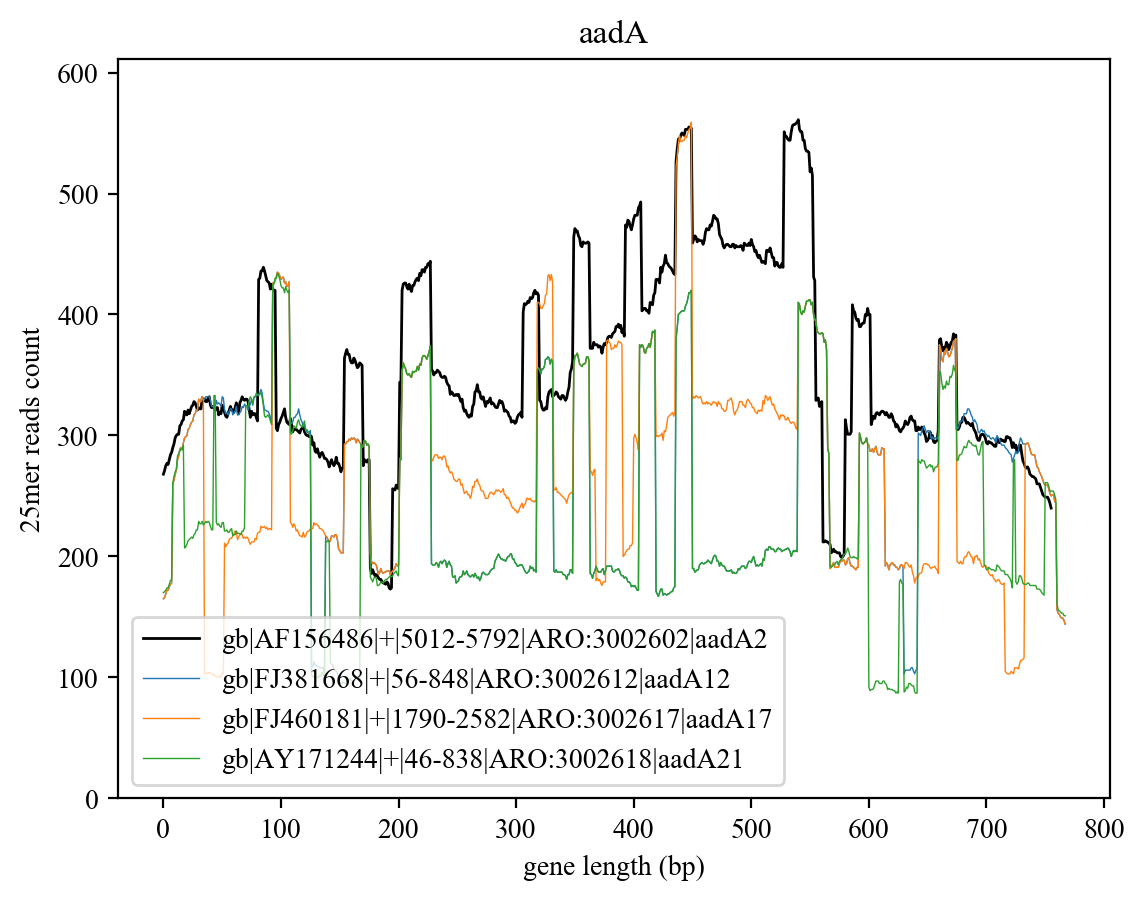

Supplement: Supplementary file 2 — Additional file 2. Archive containing files for evaluation k-mer performance and scoring generated by the k-mer method. [file 12859_2019_3335_MOESM2_ESM.zip › kmer/SJTUF10236_Typhimurium/ar_nucl_25/aadA_25mer.png]

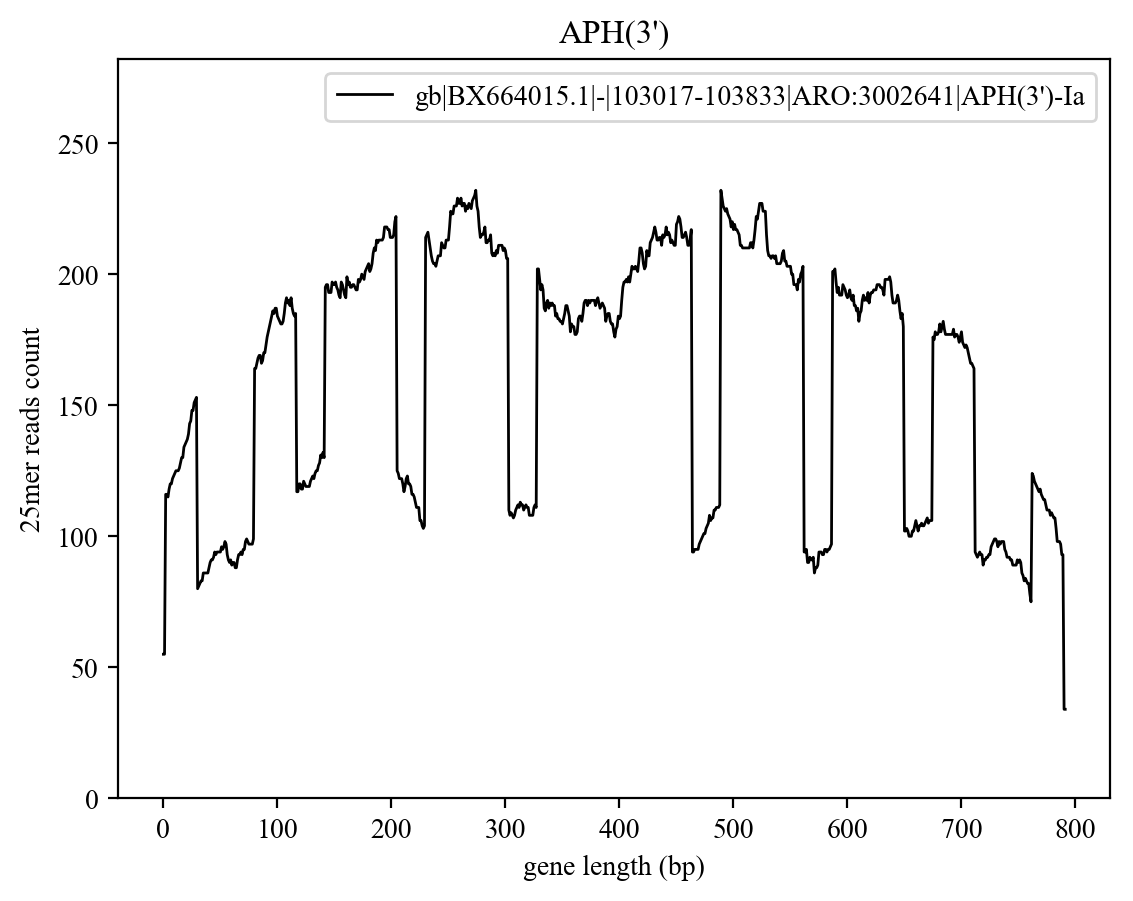

Supplement: Supplementary file 2 — Additional file 2. Archive containing files for evaluation k-mer performance and scoring generated by the k-mer method. [file 12859_2019_3335_MOESM2_ESM.zip › kmer/SJTUF10236_Typhimurium/ar_nucl_25/APH(3')_25mer.png]

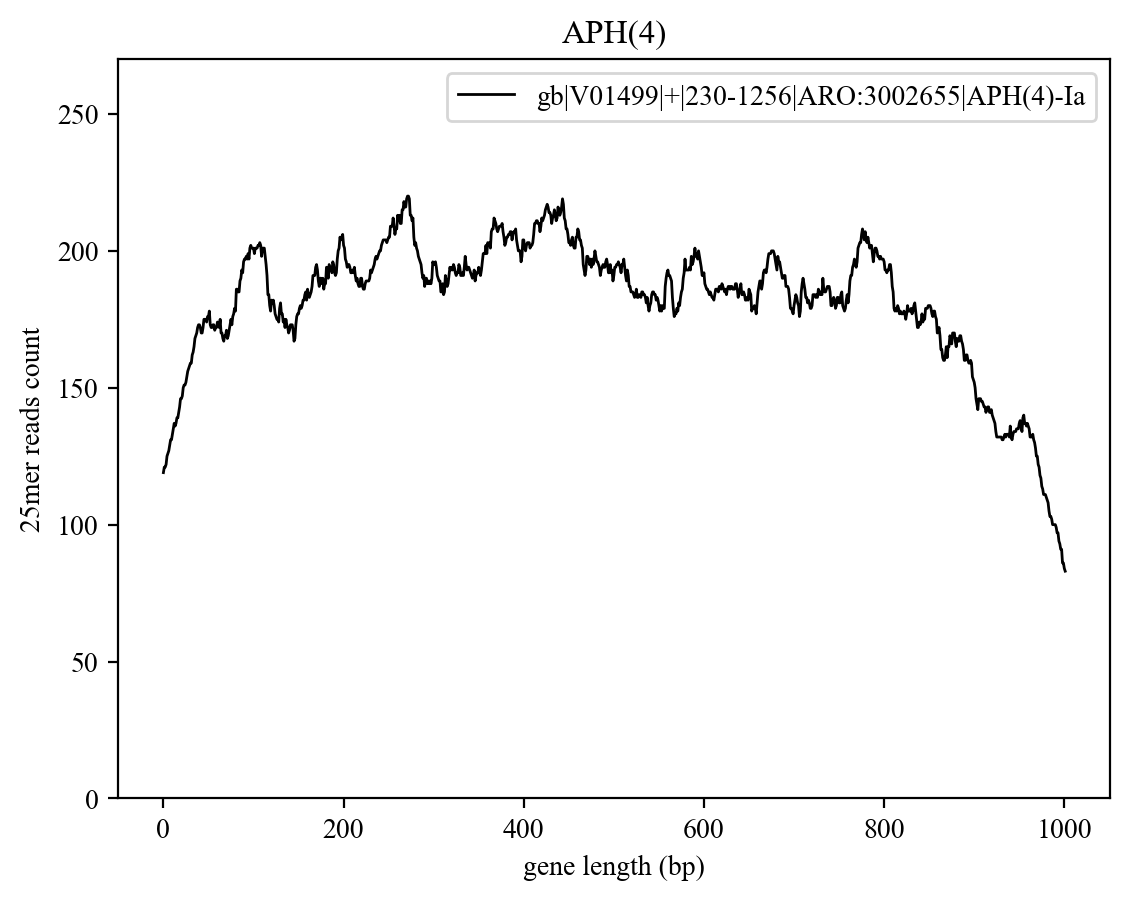

Supplement: Supplementary file 2 — Additional file 2. Archive containing files for evaluation k-mer performance and scoring generated by the k-mer method. [file 12859_2019_3335_MOESM2_ESM.zip › kmer/SJTUF10236_Typhimurium/ar_nucl_25/APH(4)_25mer.png]

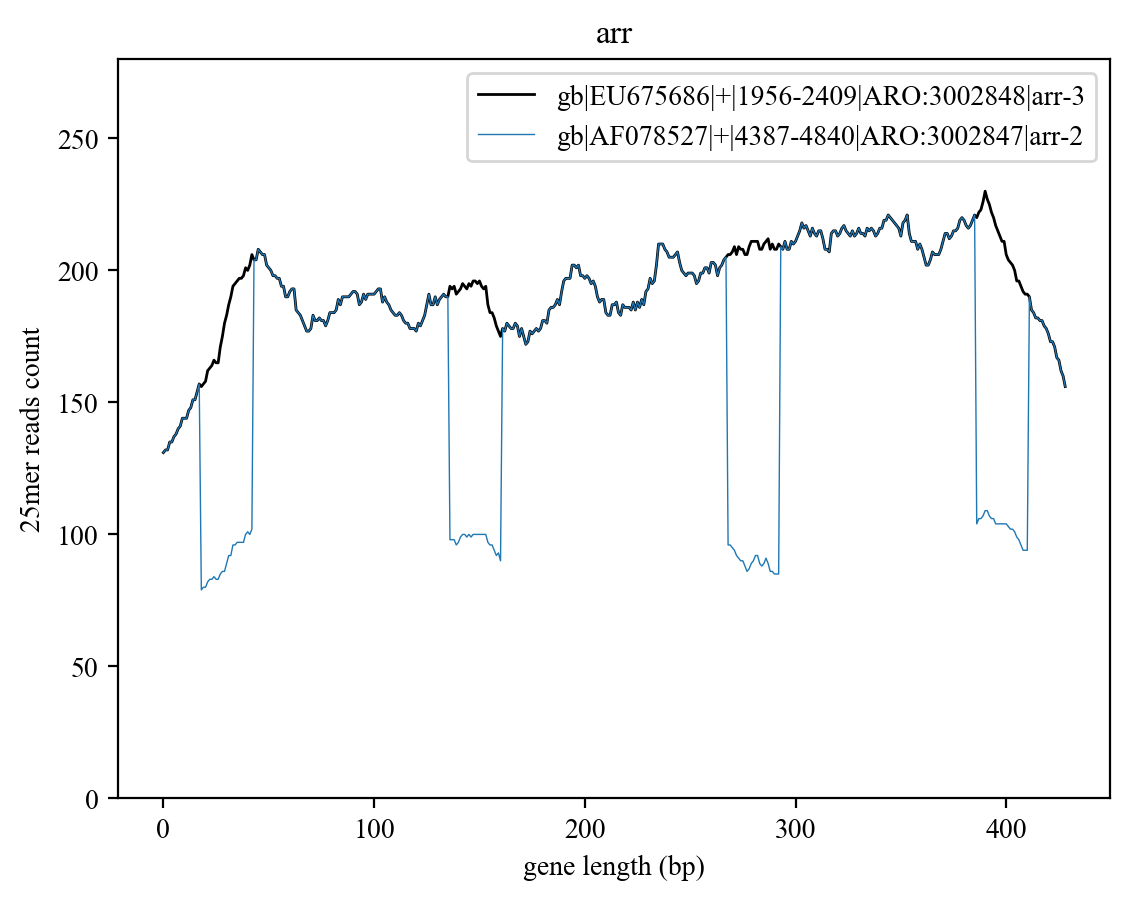

Supplement: Supplementary file 2 — Additional file 2. Archive containing files for evaluation k-mer performance and scoring generated by the k-mer method. [file 12859_2019_3335_MOESM2_ESM.zip › kmer/SJTUF10236_Typhimurium/ar_nucl_25/arr_25mer.png]

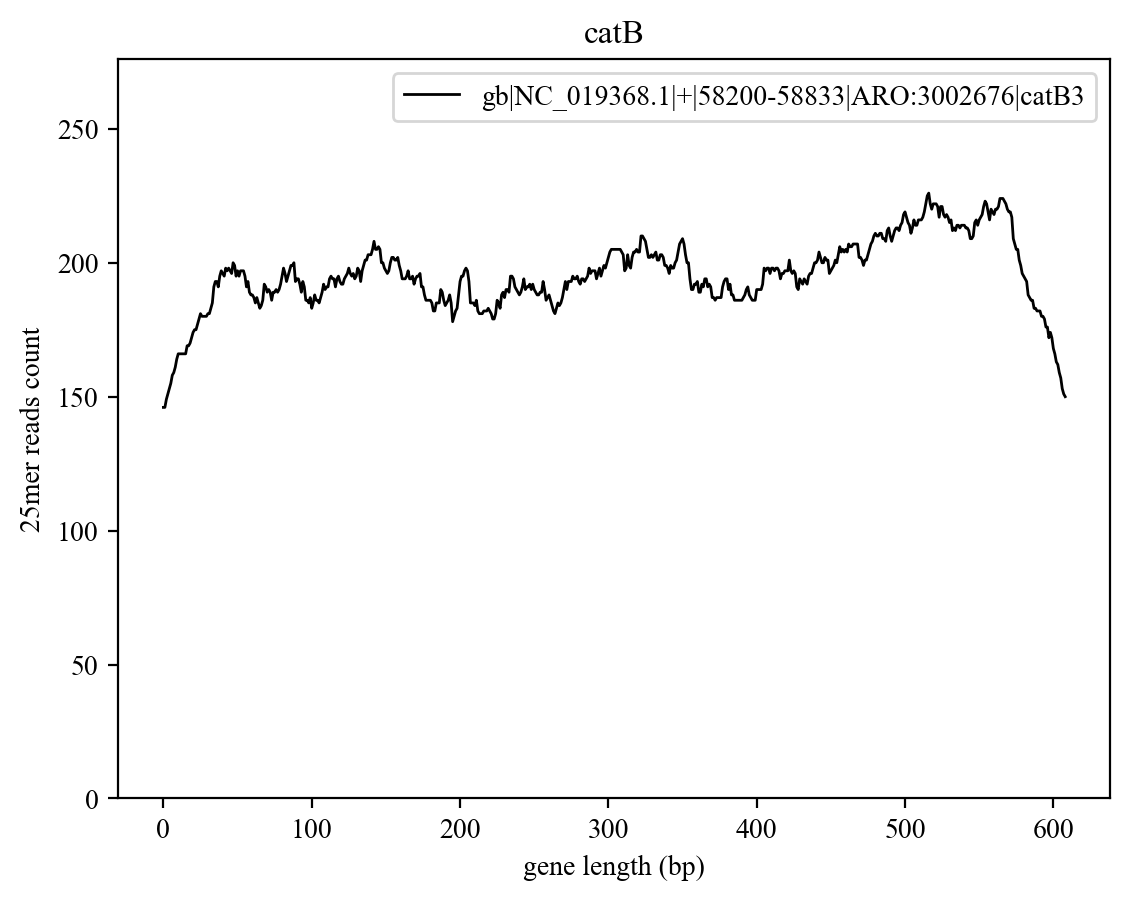

Supplement: Supplementary file 2 — Additional file 2. Archive containing files for evaluation k-mer performance and scoring generated by the k-mer method. [file 12859_2019_3335_MOESM2_ESM.zip › kmer/SJTUF10236_Typhimurium/ar_nucl_25/catB_25mer.png]

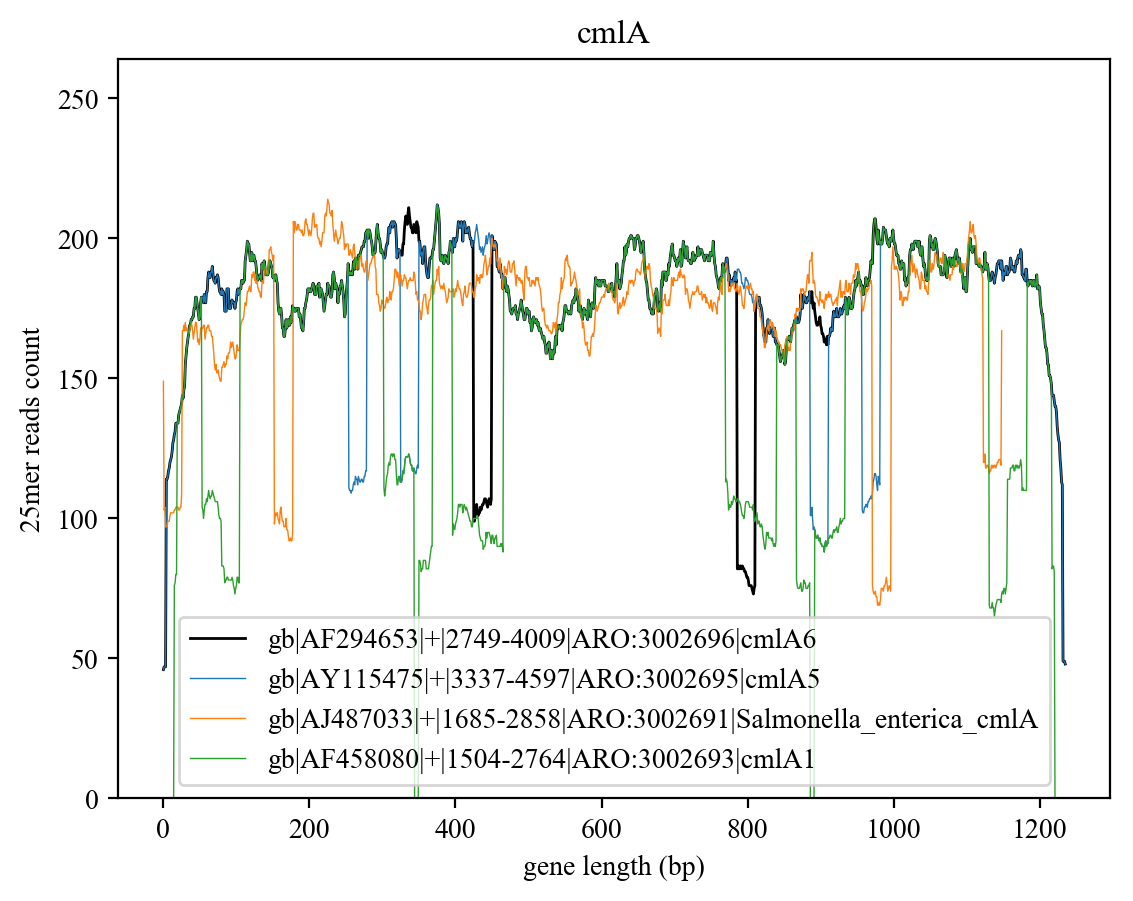

Supplement: Supplementary file 2 — Additional file 2. Archive containing files for evaluation k-mer performance and scoring generated by the k-mer method. [file 12859_2019_3335_MOESM2_ESM.zip › kmer/SJTUF10236_Typhimurium/ar_nucl_25/cmlA_25mer.png]

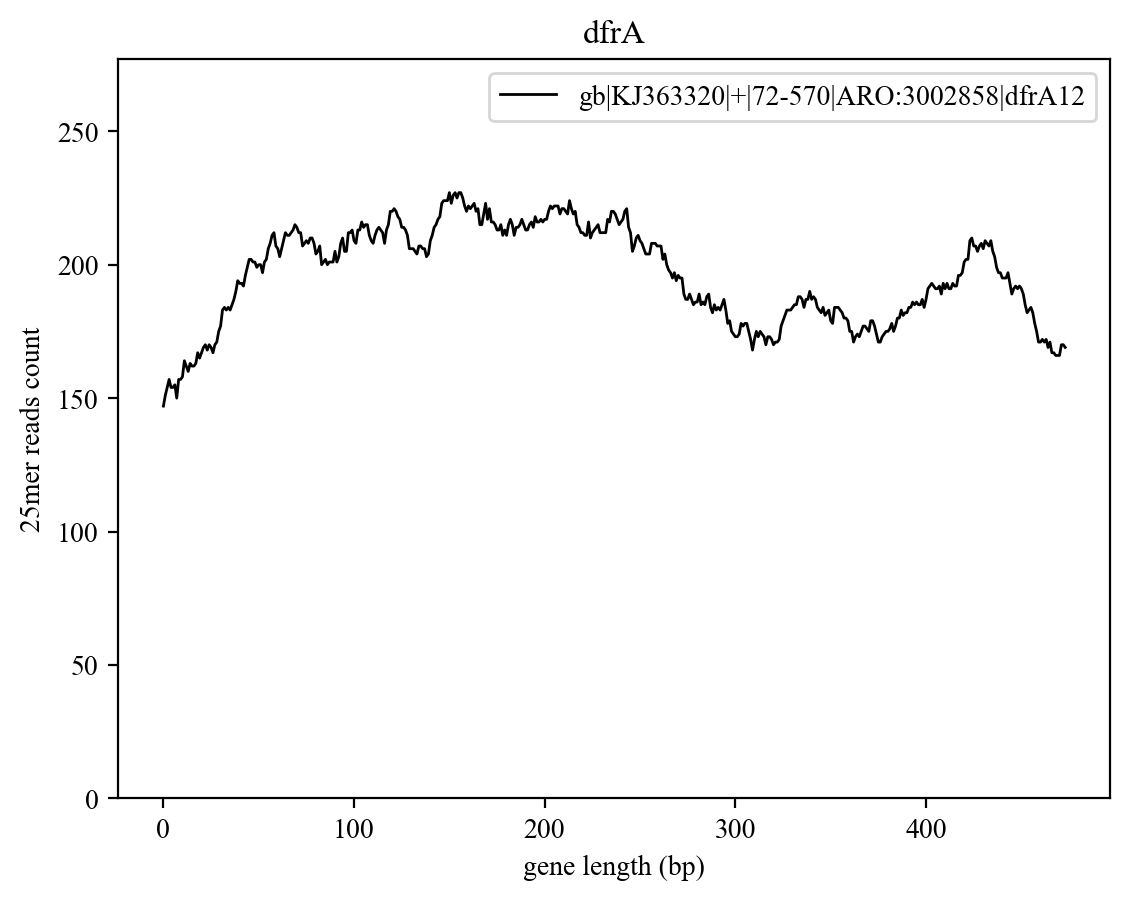

Supplement: Supplementary file 2 — Additional file 2. Archive containing files for evaluation k-mer performance and scoring generated by the k-mer method. [file 12859_2019_3335_MOESM2_ESM.zip › kmer/SJTUF10236_Typhimurium/ar_nucl_25/dfrA_25mer.png]

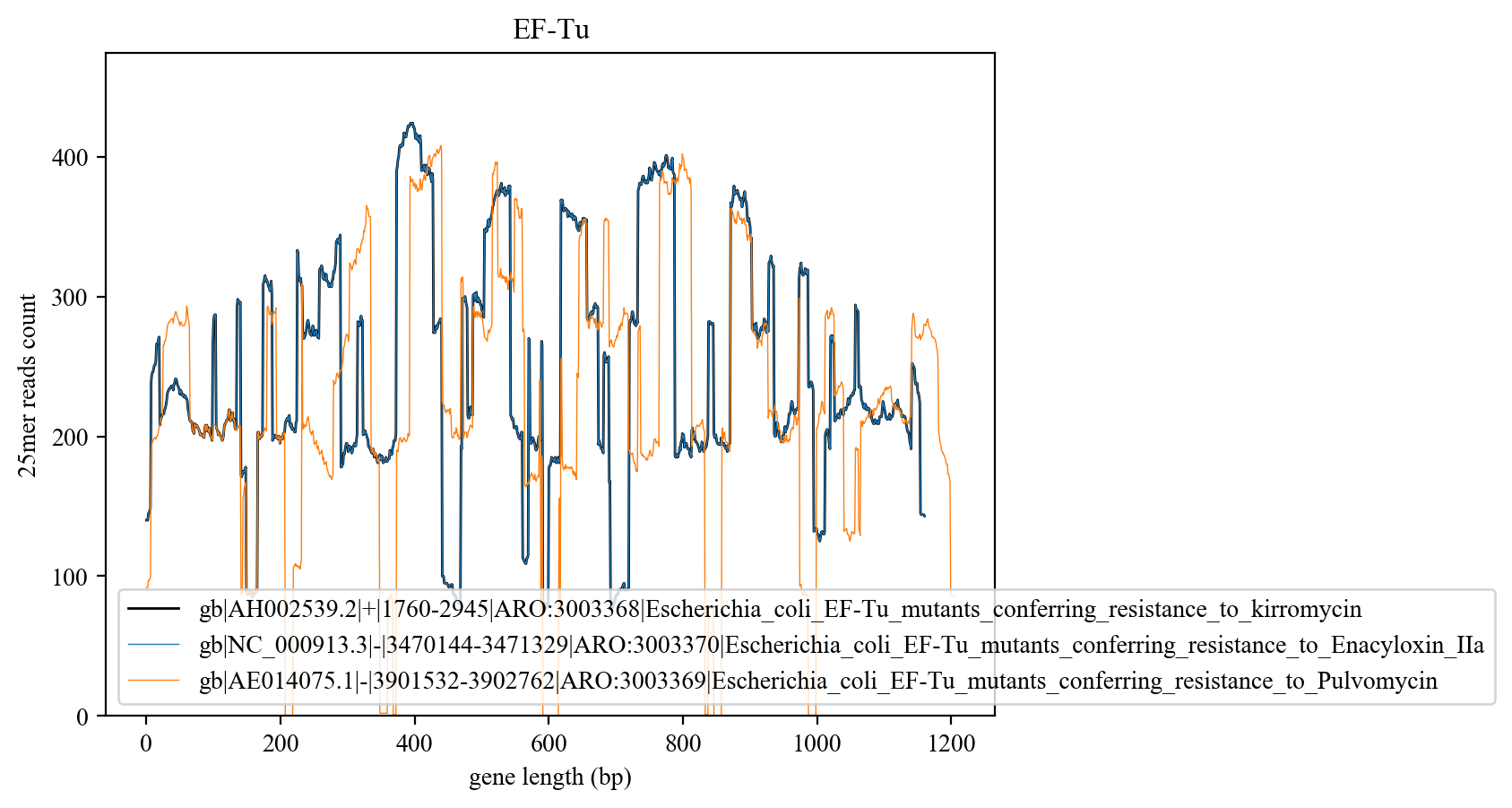

Supplement: Supplementary file 2 — Additional file 2. Archive containing files for evaluation k-mer performance and scoring generated by the k-mer method. [file 12859_2019_3335_MOESM2_ESM.zip › kmer/SJTUF10236_Typhimurium/ar_nucl_25/EF-Tu_25mer.png]

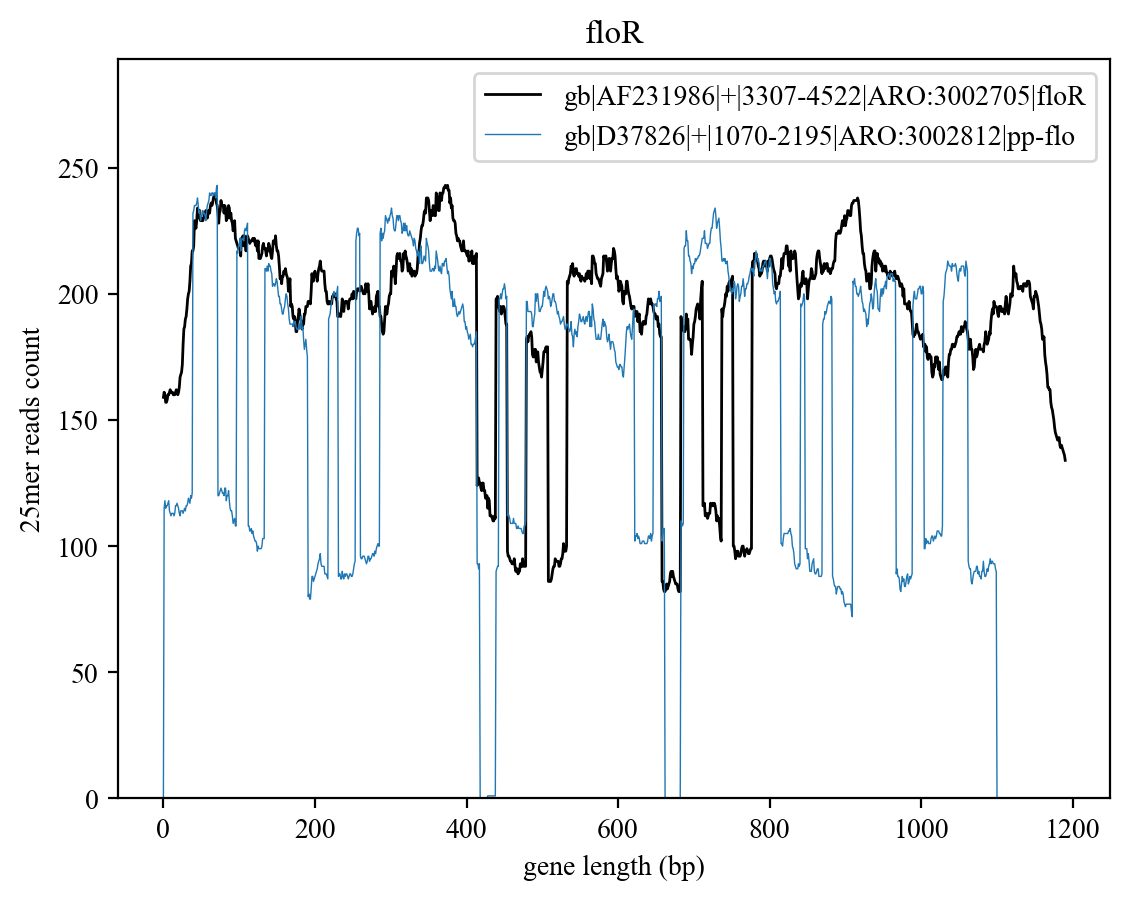

Supplement: Supplementary file 2 — Additional file 2. Archive containing files for evaluation k-mer performance and scoring generated by the k-mer method. [file 12859_2019_3335_MOESM2_ESM.zip › kmer/SJTUF10236_Typhimurium/ar_nucl_25/floR_25mer.png]

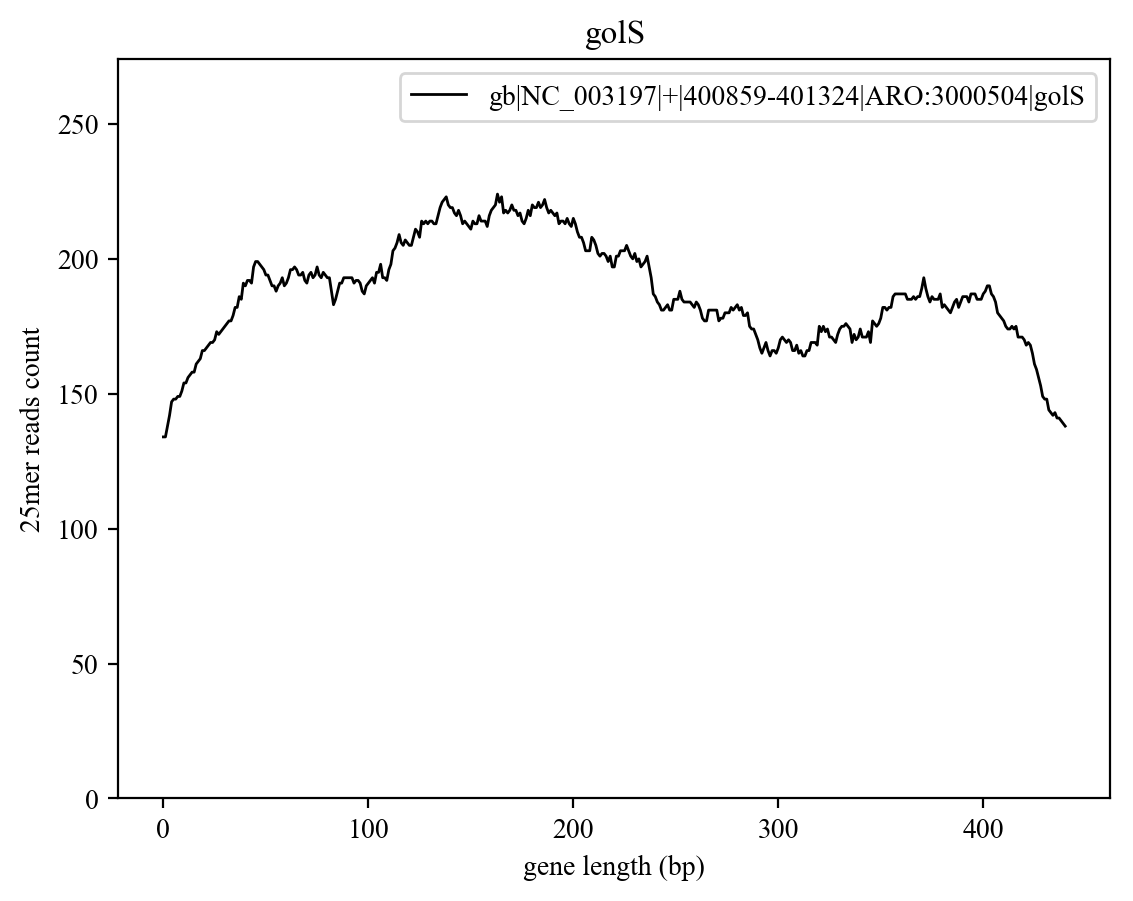

Supplement: Supplementary file 2 — Additional file 2. Archive containing files for evaluation k-mer performance and scoring generated by the k-mer method. [file 12859_2019_3335_MOESM2_ESM.zip › kmer/SJTUF10236_Typhimurium/ar_nucl_25/golS_25mer.png]

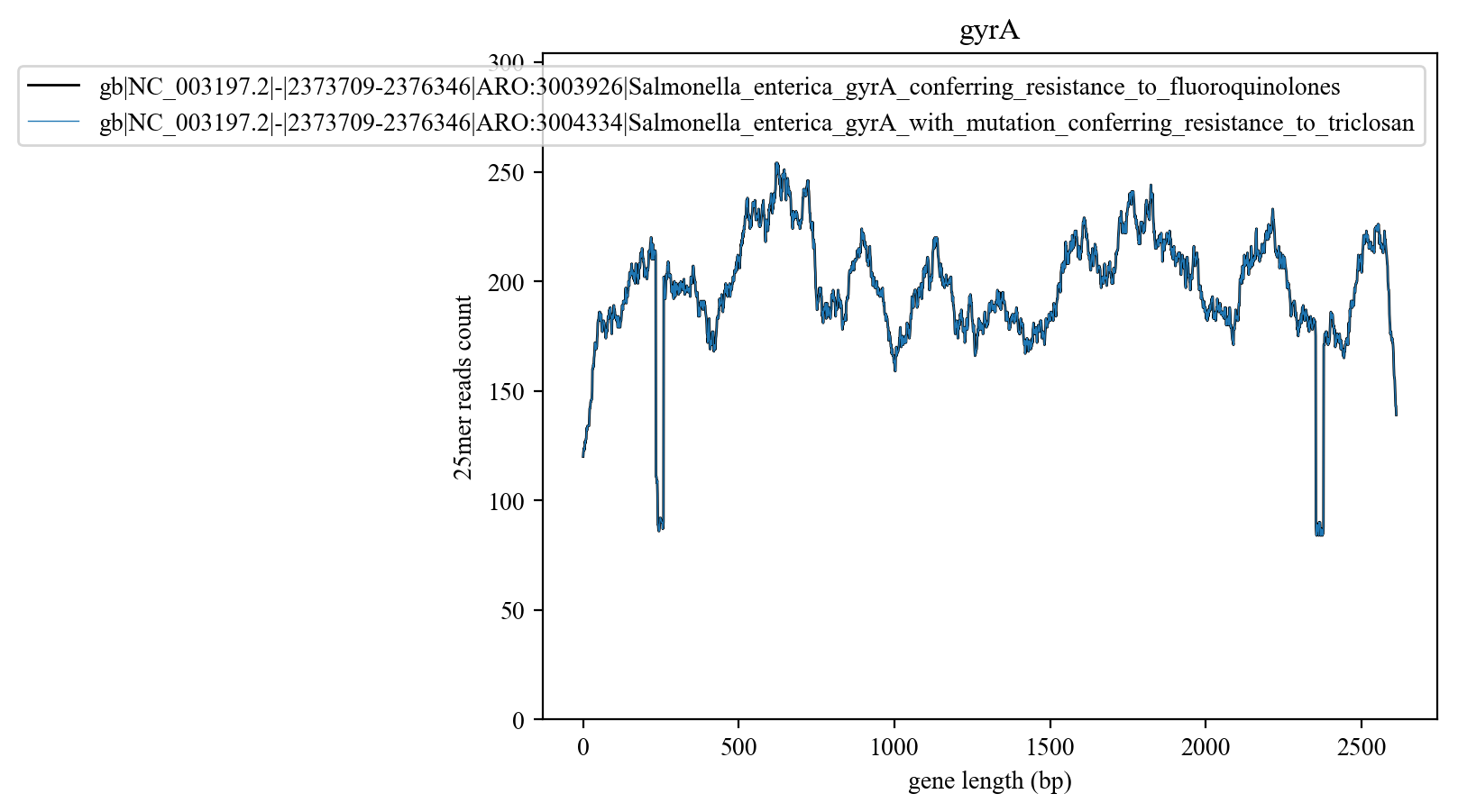

Supplement: Supplementary file 2 — Additional file 2. Archive containing files for evaluation k-mer performance and scoring generated by the k-mer method. [file 12859_2019_3335_MOESM2_ESM.zip › kmer/SJTUF10236_Typhimurium/ar_nucl_25/gyrA_25mer.png]

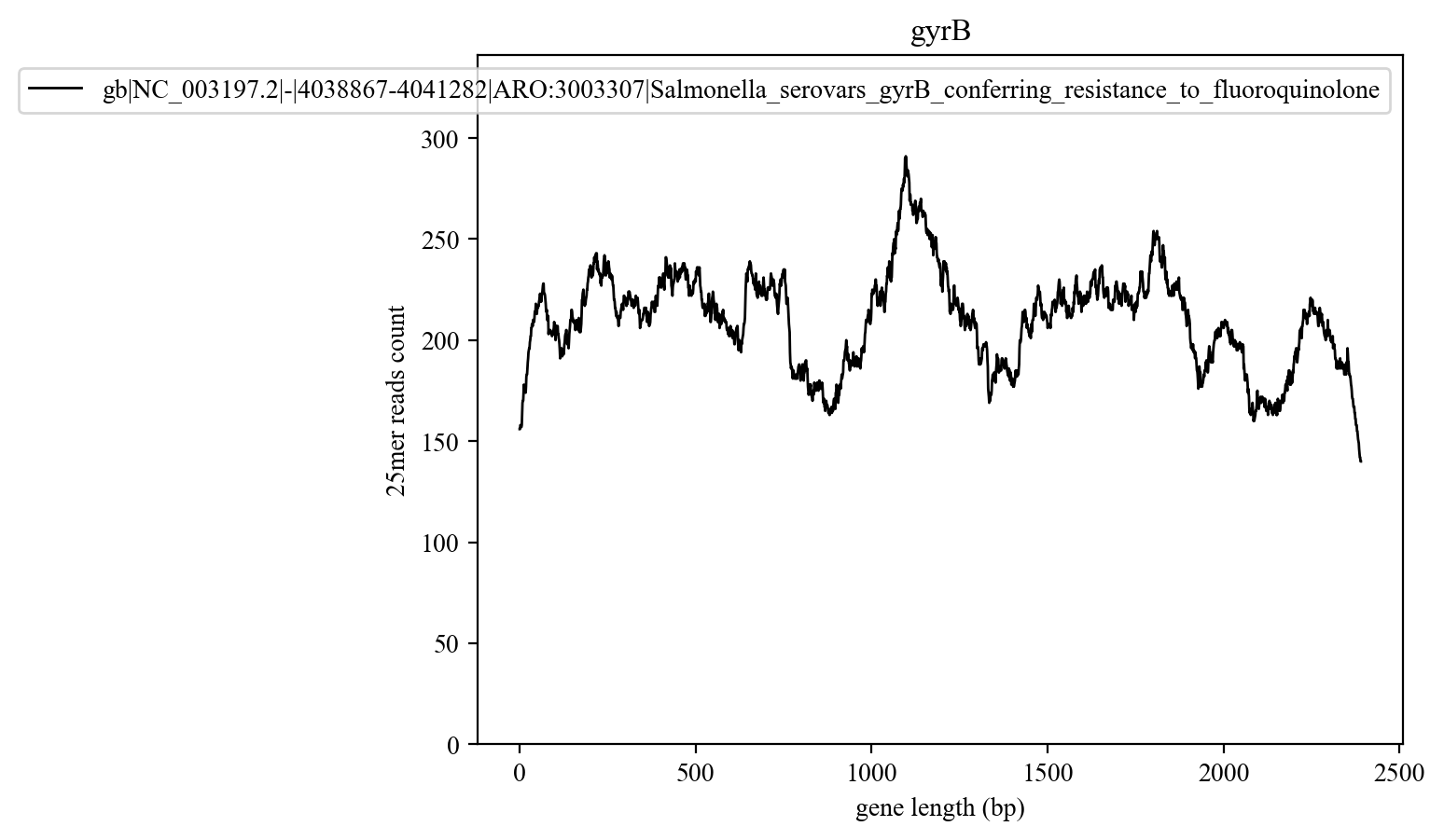

Supplement: Supplementary file 2 — Additional file 2. Archive containing files for evaluation k-mer performance and scoring generated by the k-mer method. [file 12859_2019_3335_MOESM2_ESM.zip › kmer/SJTUF10236_Typhimurium/ar_nucl_25/gyrB_25mer.png]

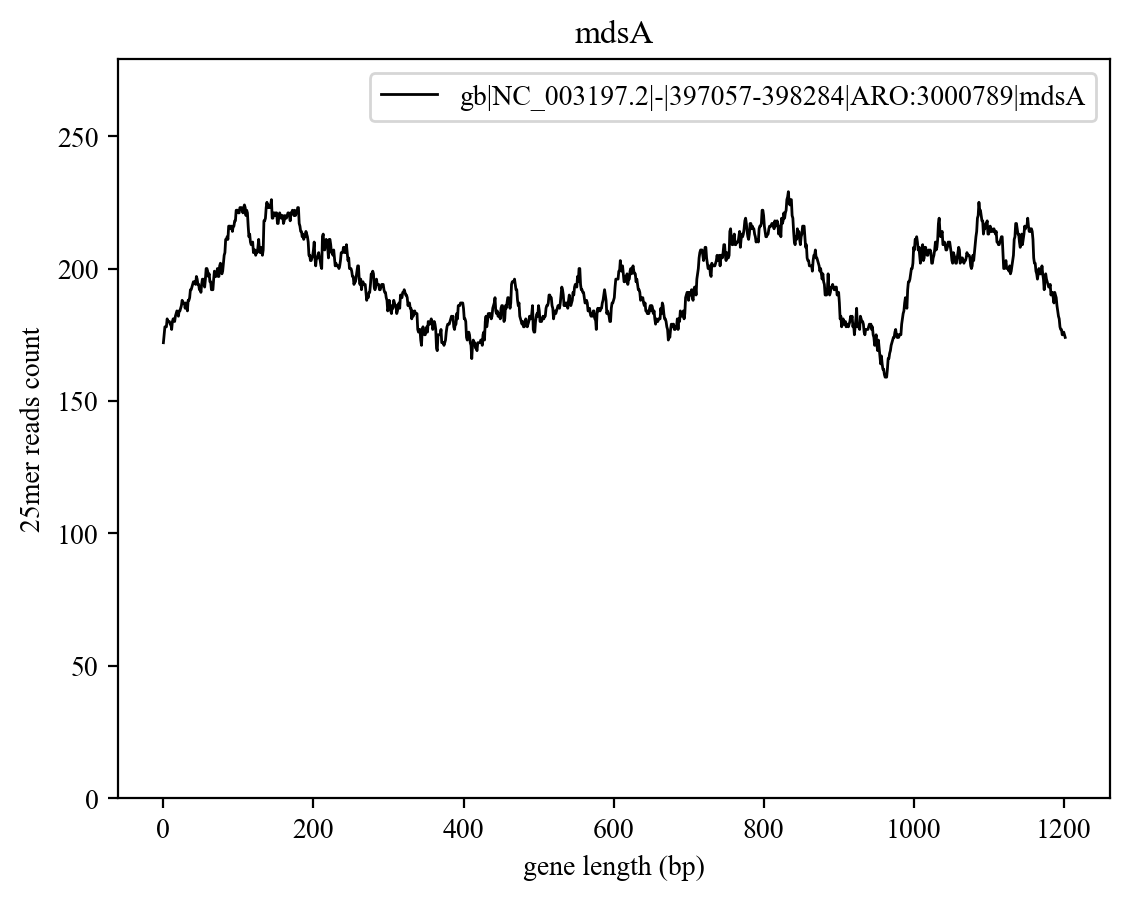

Supplement: Supplementary file 2 — Additional file 2. Archive containing files for evaluation k-mer performance and scoring generated by the k-mer method. [file 12859_2019_3335_MOESM2_ESM.zip › kmer/SJTUF10236_Typhimurium/ar_nucl_25/mdsA_25mer.png]

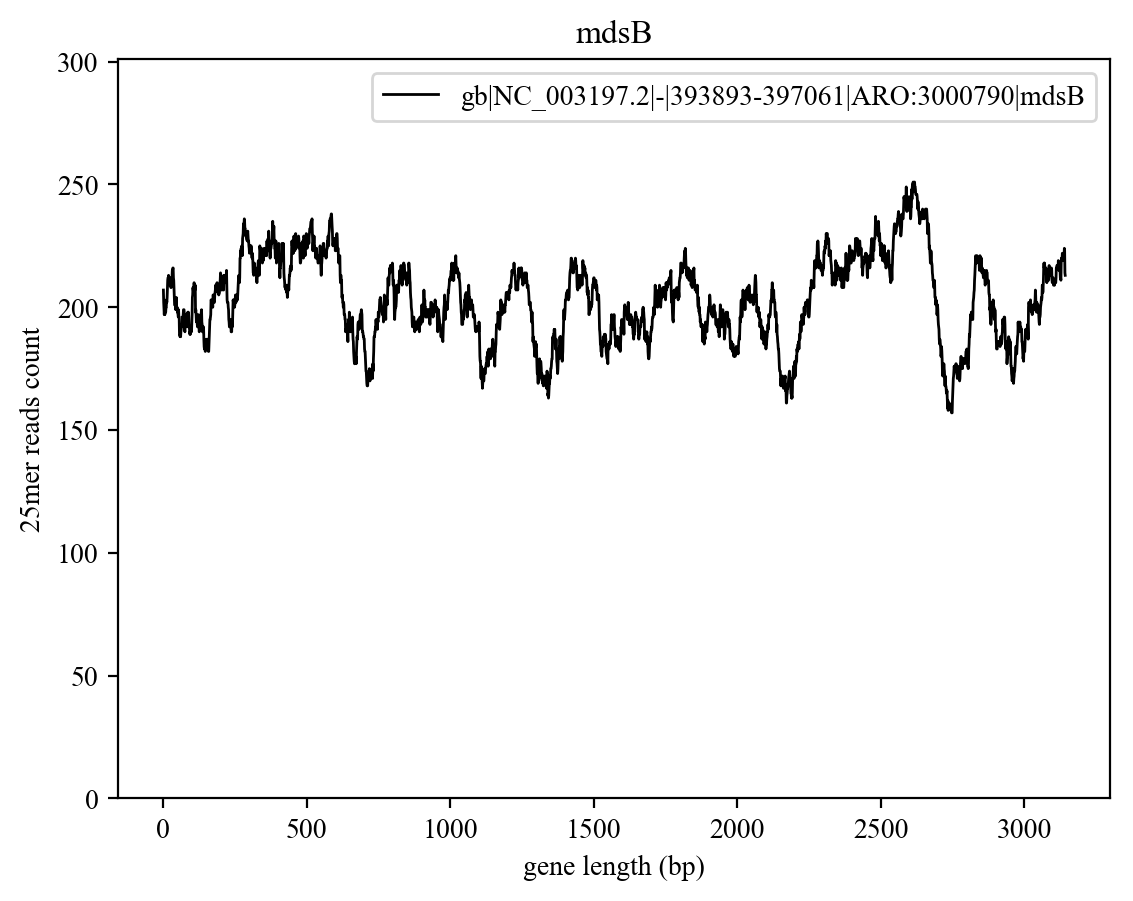

Supplement: Supplementary file 2 — Additional file 2. Archive containing files for evaluation k-mer performance and scoring generated by the k-mer method. [file 12859_2019_3335_MOESM2_ESM.zip › kmer/SJTUF10236_Typhimurium/ar_nucl_25/mdsB_25mer.png]

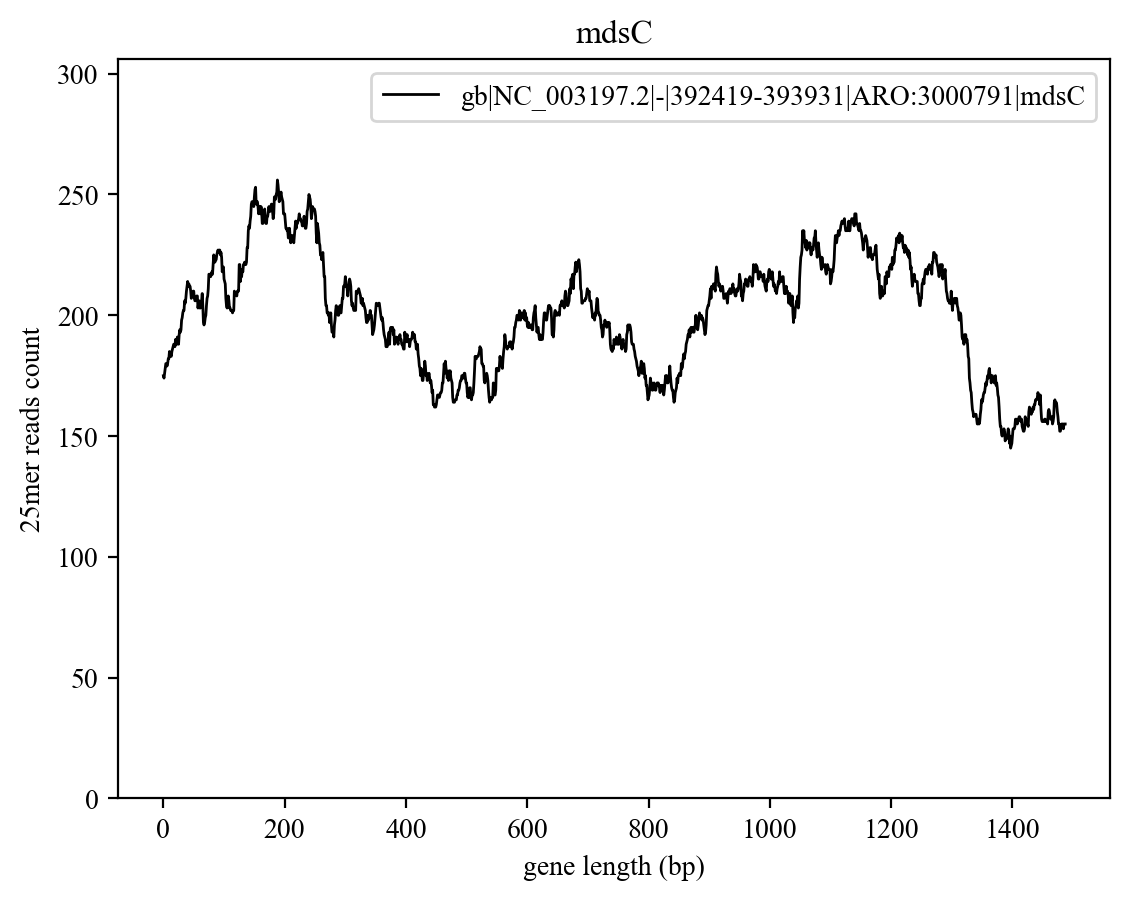

Supplement: Supplementary file 2 — Additional file 2. Archive containing files for evaluation k-mer performance and scoring generated by the k-mer method. [file 12859_2019_3335_MOESM2_ESM.zip › kmer/SJTUF10236_Typhimurium/ar_nucl_25/mdsC_25mer.png]

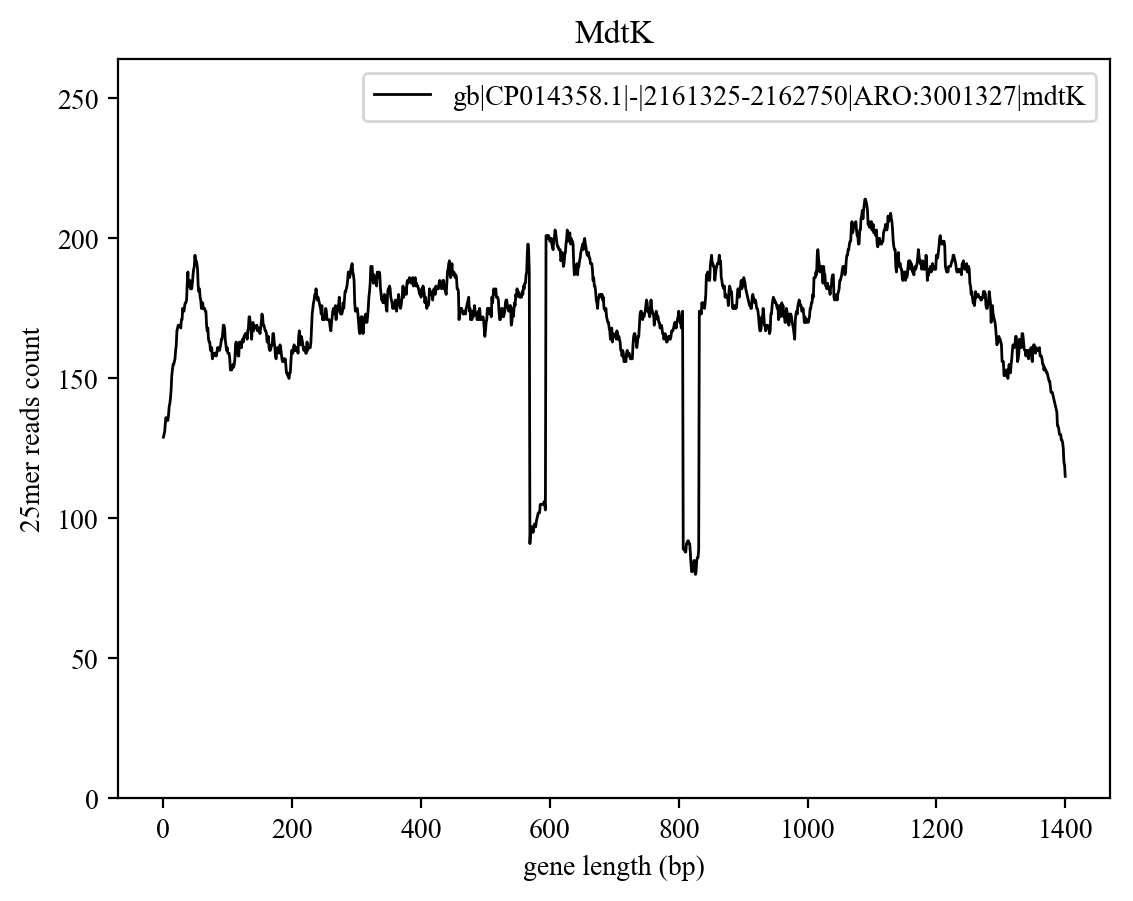

Supplement: Supplementary file 2 — Additional file 2. Archive containing files for evaluation k-mer performance and scoring generated by the k-mer method. [file 12859_2019_3335_MOESM2_ESM.zip › kmer/SJTUF10236_Typhimurium/ar_nucl_25/MdtK_25mer.png]

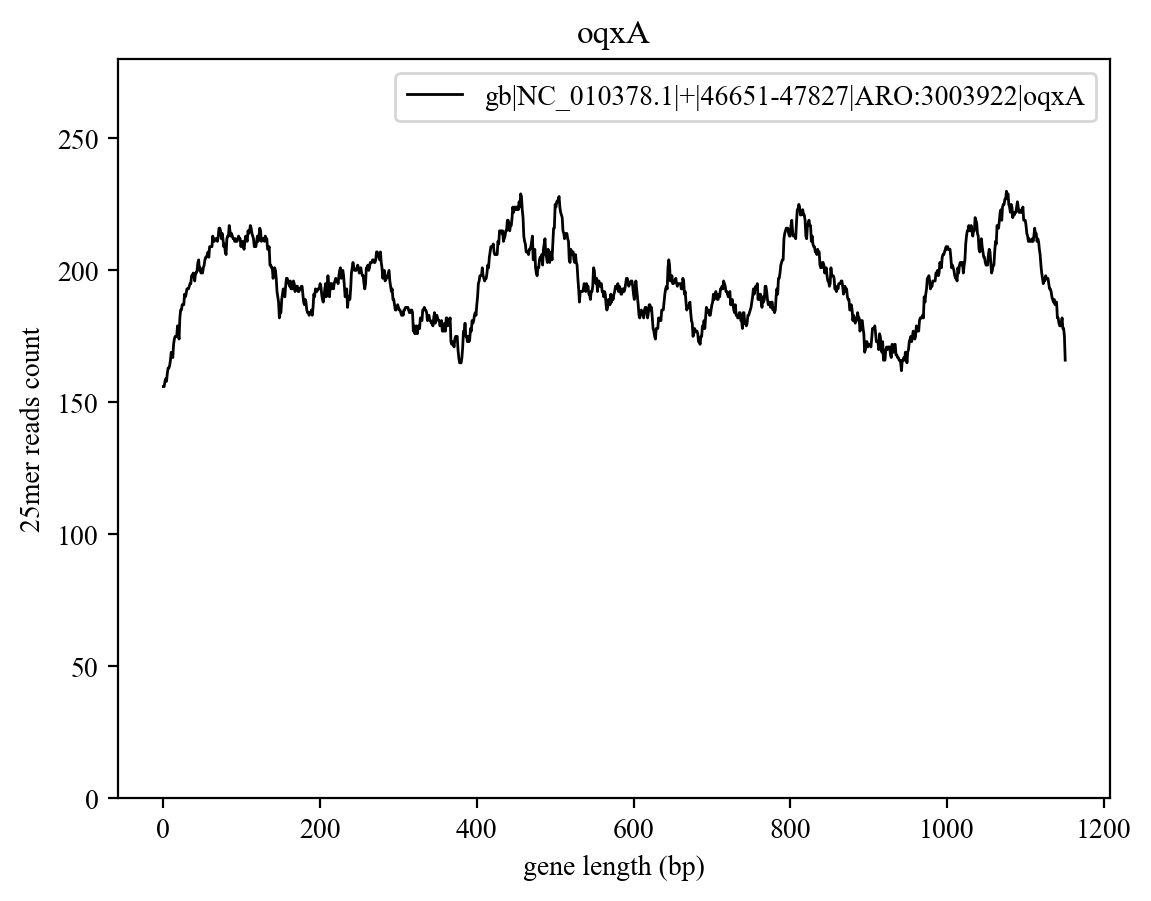

Supplement: Supplementary file 2 — Additional file 2. Archive containing files for evaluation k-mer performance and scoring generated by the k-mer method. [file 12859_2019_3335_MOESM2_ESM.zip › kmer/SJTUF10236_Typhimurium/ar_nucl_25/oqxA_25mer.png]

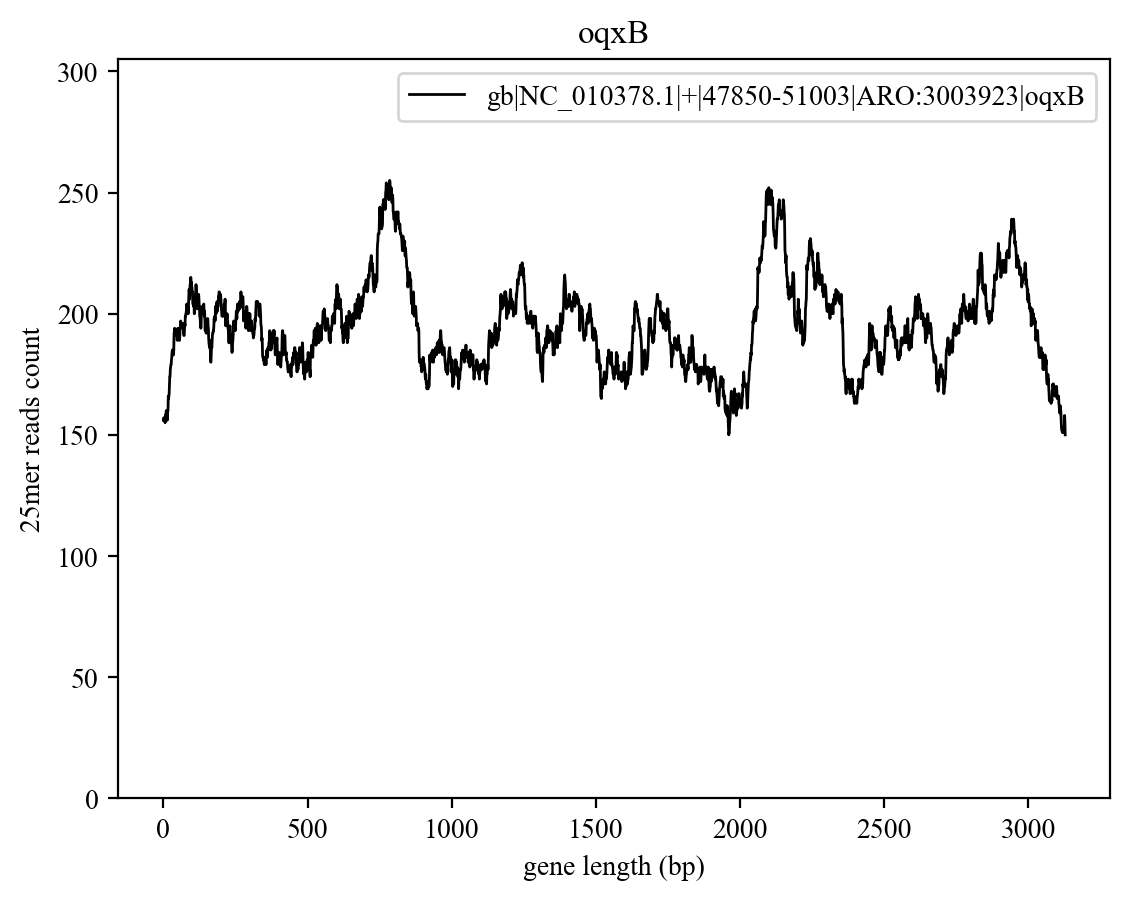

Supplement: Supplementary file 2 — Additional file 2. Archive containing files for evaluation k-mer performance and scoring generated by the k-mer method. [file 12859_2019_3335_MOESM2_ESM.zip › kmer/SJTUF10236_Typhimurium/ar_nucl_25/oqxB_25mer.png]

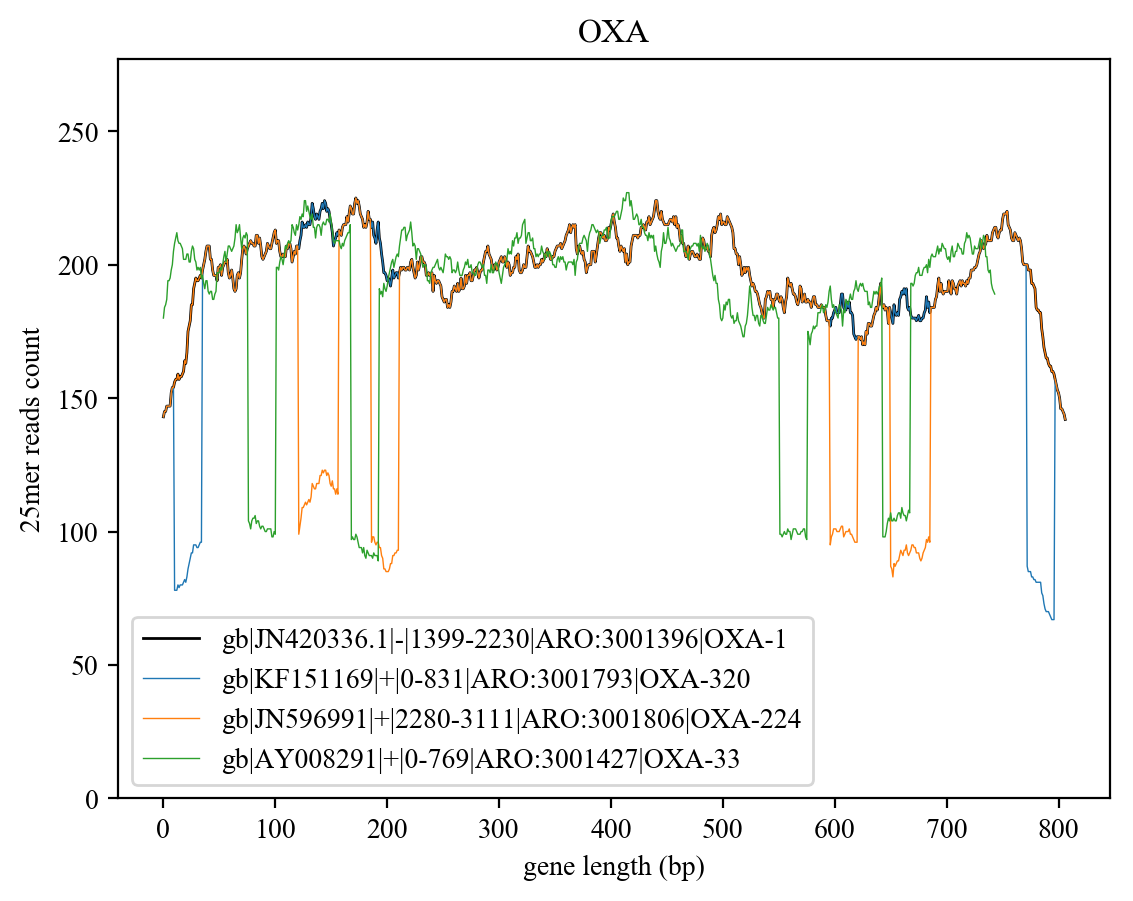

Supplement: Supplementary file 2 — Additional file 2. Archive containing files for evaluation k-mer performance and scoring generated by the k-mer method. [file 12859_2019_3335_MOESM2_ESM.zip › kmer/SJTUF10236_Typhimurium/ar_nucl_25/OXA_25mer.png]

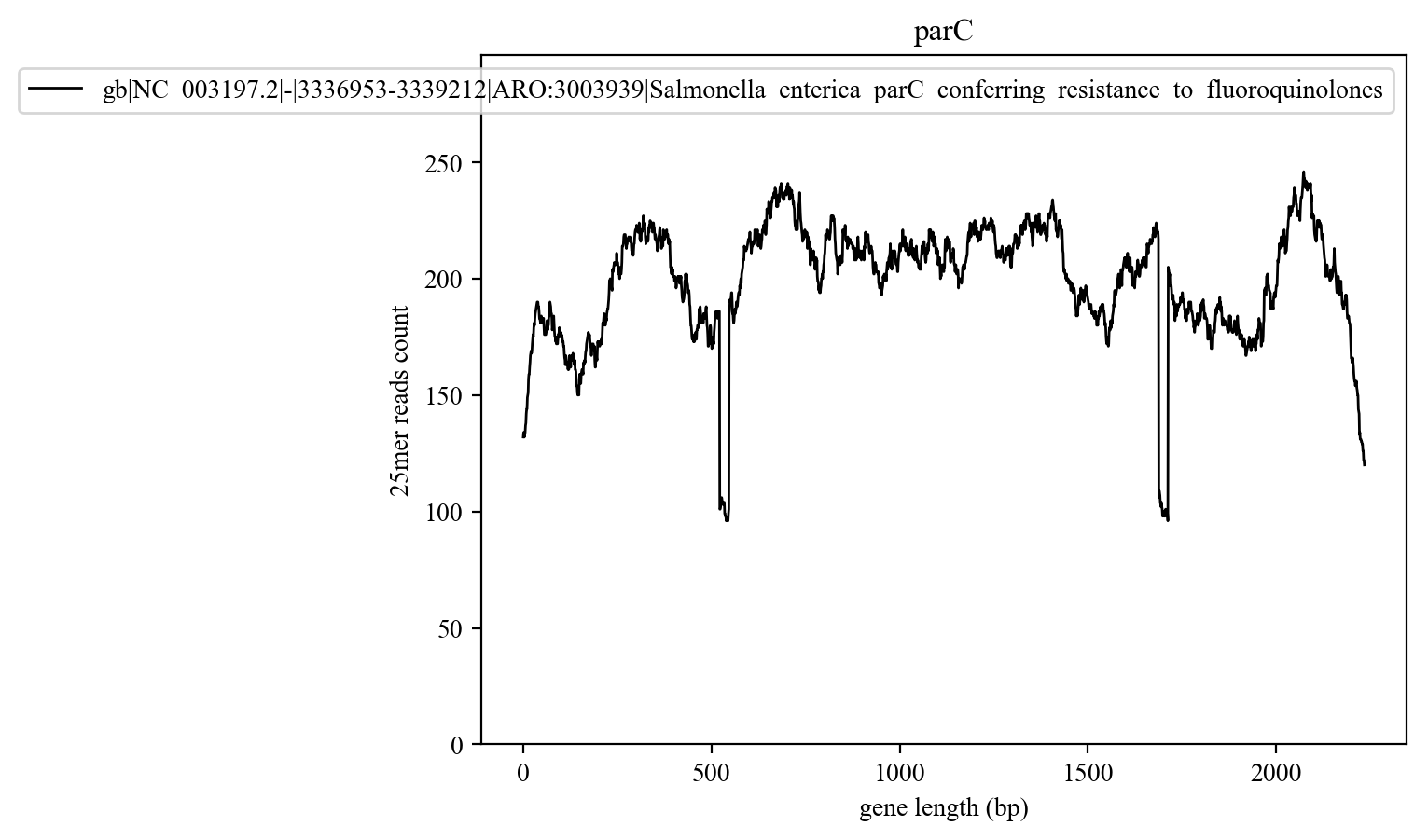

Supplement: Supplementary file 2 — Additional file 2. Archive containing files for evaluation k-mer performance and scoring generated by the k-mer method. [file 12859_2019_3335_MOESM2_ESM.zip › kmer/SJTUF10236_Typhimurium/ar_nucl_25/parC_25mer.png]

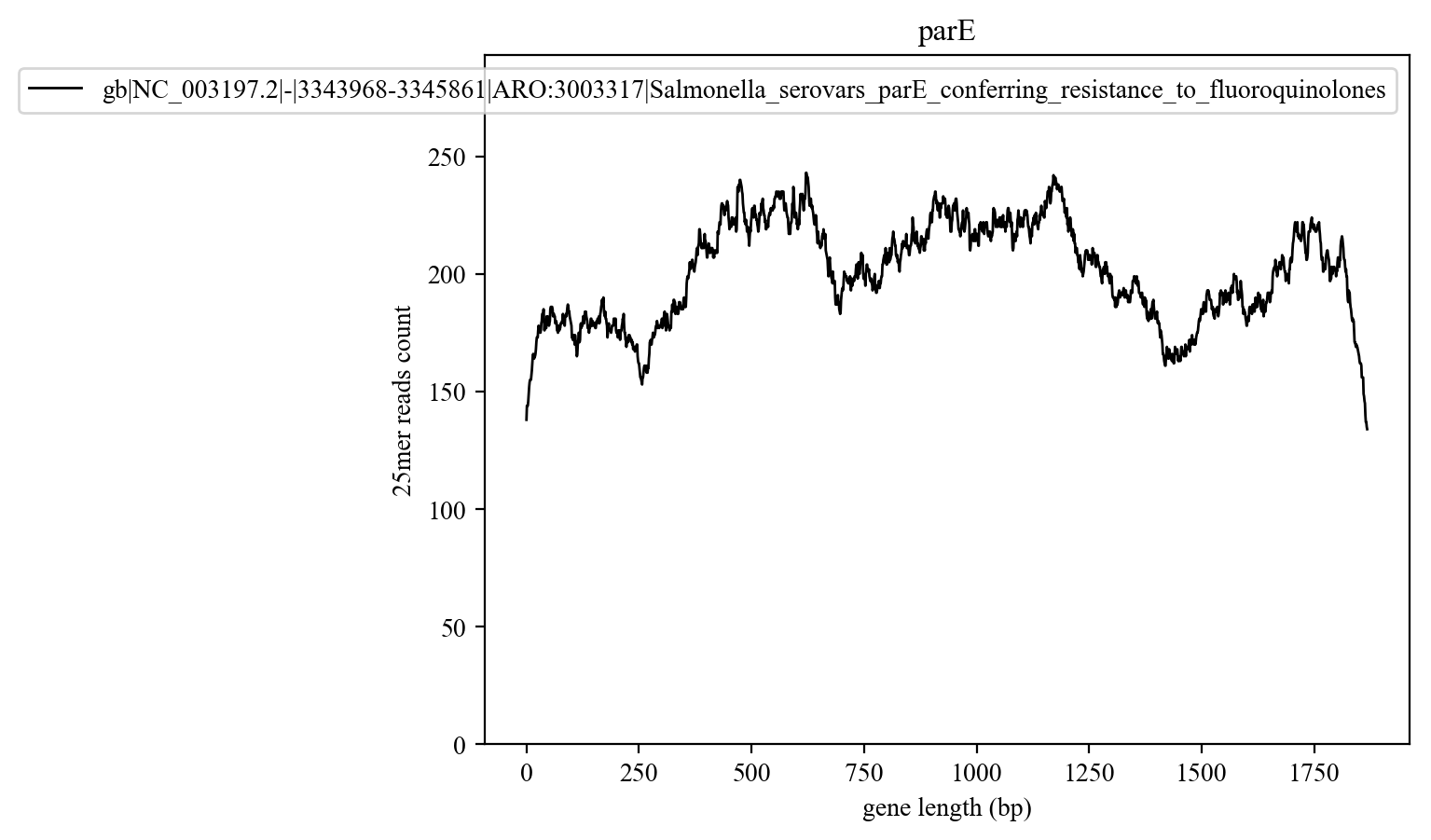

Supplement: Supplementary file 2 — Additional file 2. Archive containing files for evaluation k-mer performance and scoring generated by the k-mer method. [file 12859_2019_3335_MOESM2_ESM.zip › kmer/SJTUF10236_Typhimurium/ar_nucl_25/parE_25mer.png]

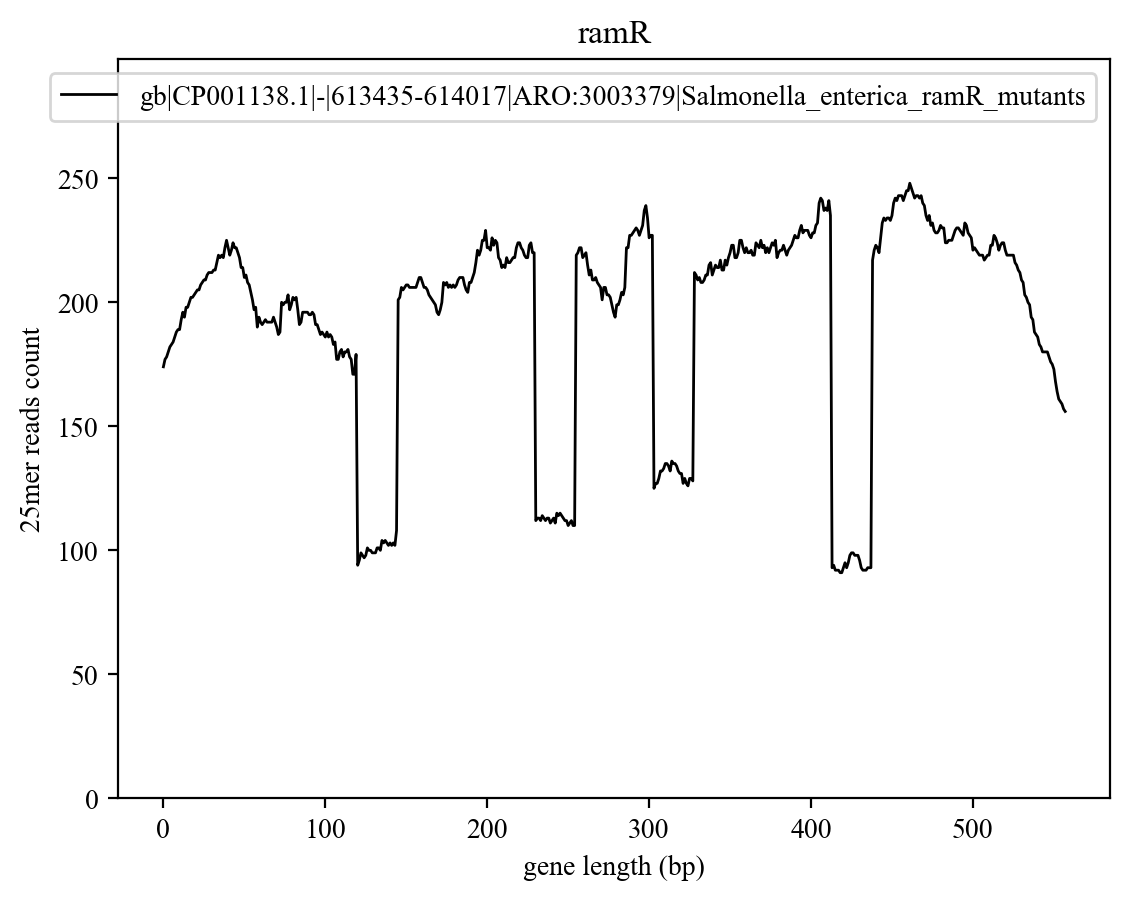

Supplement: Supplementary file 2 — Additional file 2. Archive containing files for evaluation k-mer performance and scoring generated by the k-mer method. [file 12859_2019_3335_MOESM2_ESM.zip › kmer/SJTUF10236_Typhimurium/ar_nucl_25/ramR_25mer.png]

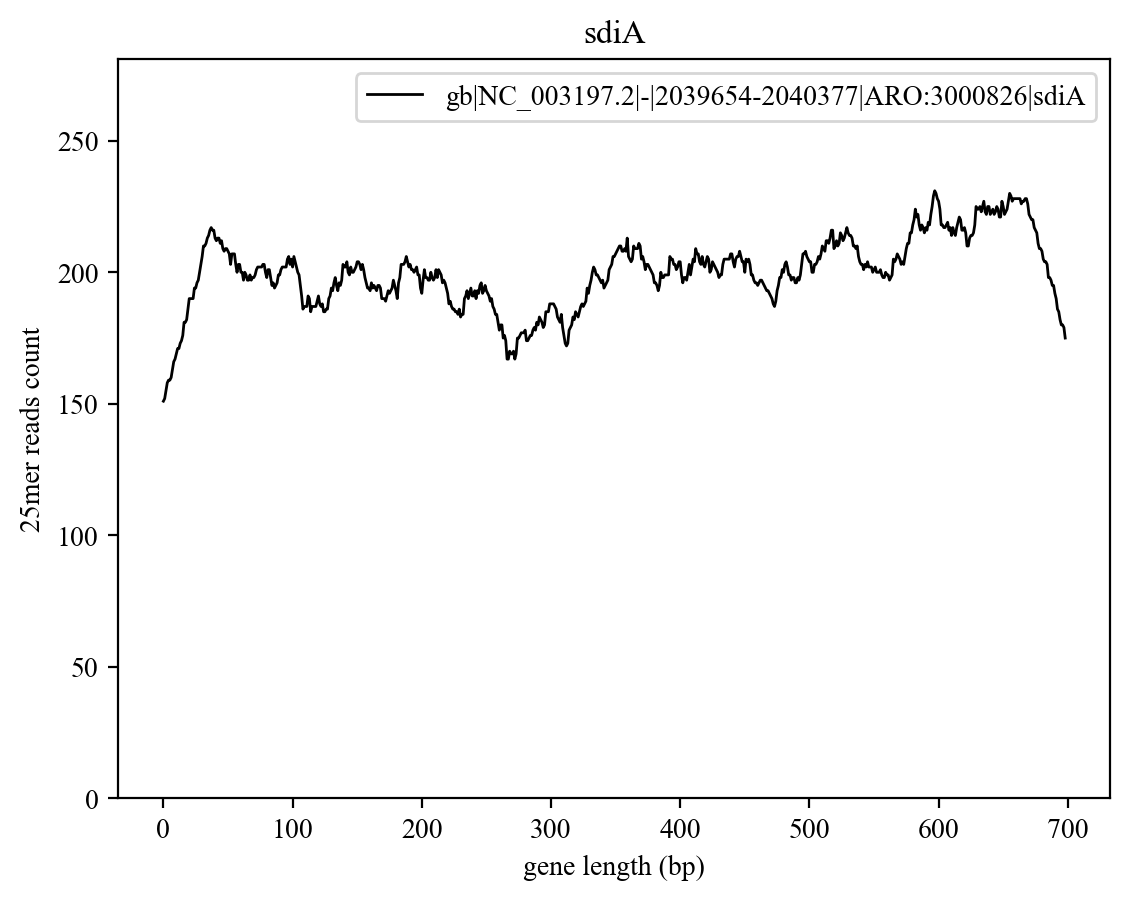

Supplement: Supplementary file 2 — Additional file 2. Archive containing files for evaluation k-mer performance and scoring generated by the k-mer method. [file 12859_2019_3335_MOESM2_ESM.zip › kmer/SJTUF10236_Typhimurium/ar_nucl_25/sdiA_25mer.png]

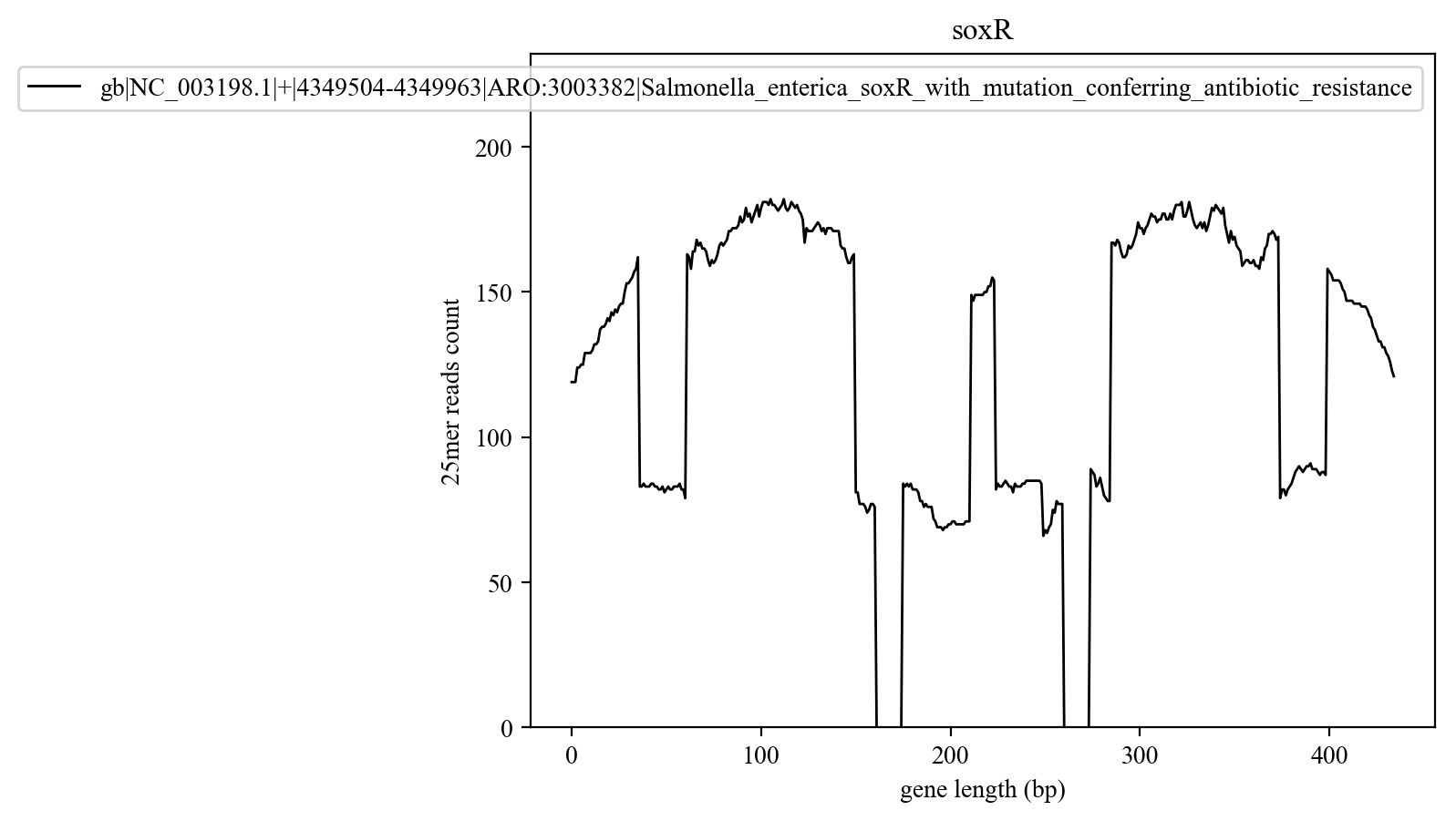

Supplement: Supplementary file 2 — Additional file 2. Archive containing files for evaluation k-mer performance and scoring generated by the k-mer method. [file 12859_2019_3335_MOESM2_ESM.zip › kmer/SJTUF10236_Typhimurium/ar_nucl_25/soxR_25mer.png]

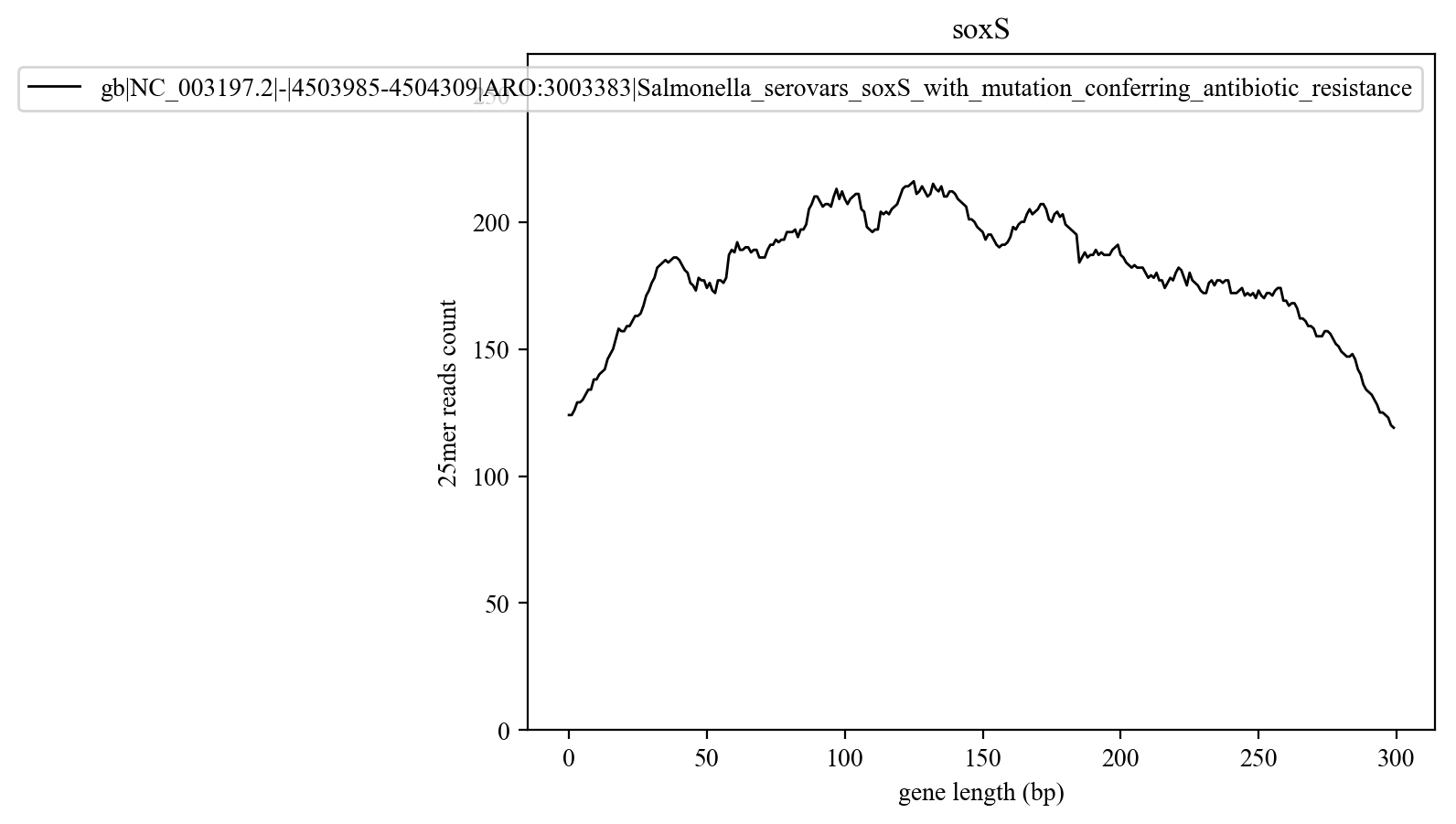

Supplement: Supplementary file 2 — Additional file 2. Archive containing files for evaluation k-mer performance and scoring generated by the k-mer method. [file 12859_2019_3335_MOESM2_ESM.zip › kmer/SJTUF10236_Typhimurium/ar_nucl_25/soxS_25mer.png]

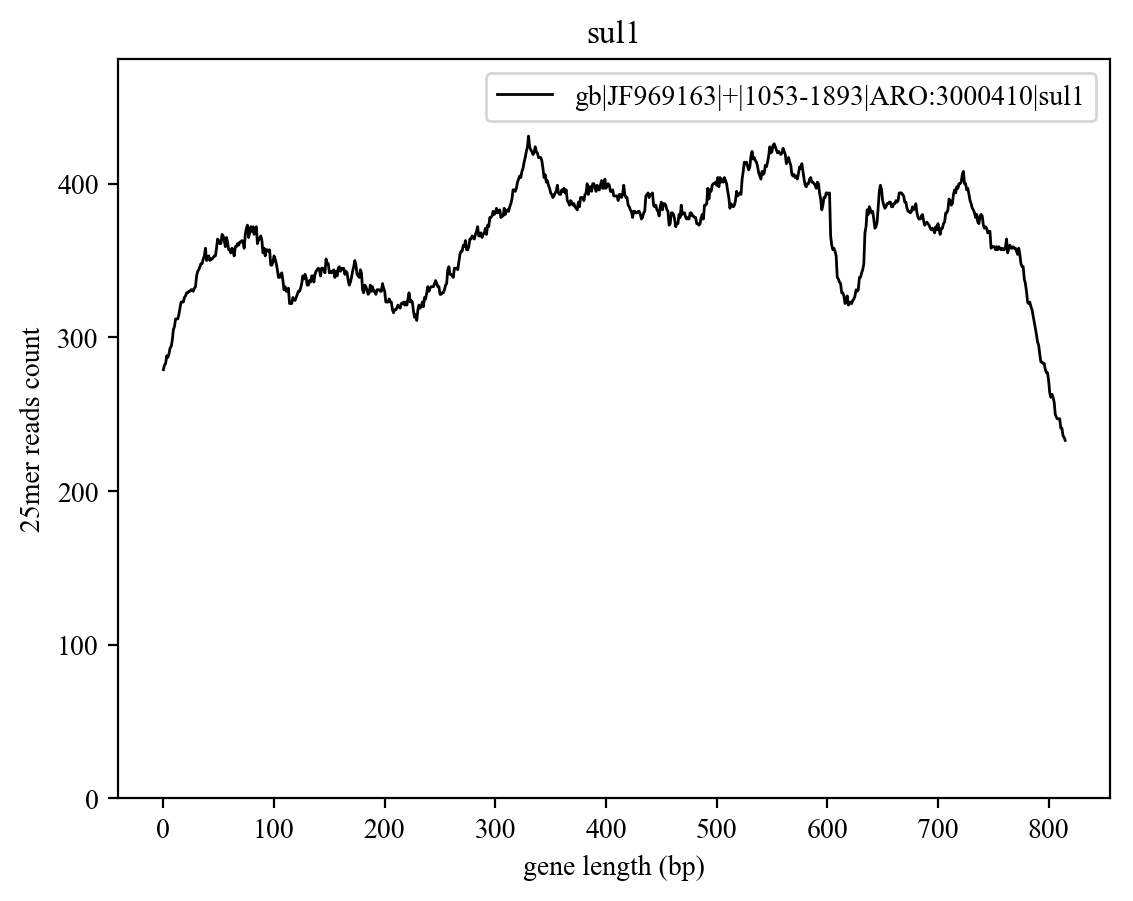

Supplement: Supplementary file 2 — Additional file 2. Archive containing files for evaluation k-mer performance and scoring generated by the k-mer method. [file 12859_2019_3335_MOESM2_ESM.zip › kmer/SJTUF10236_Typhimurium/ar_nucl_25/sul1_25mer.png]

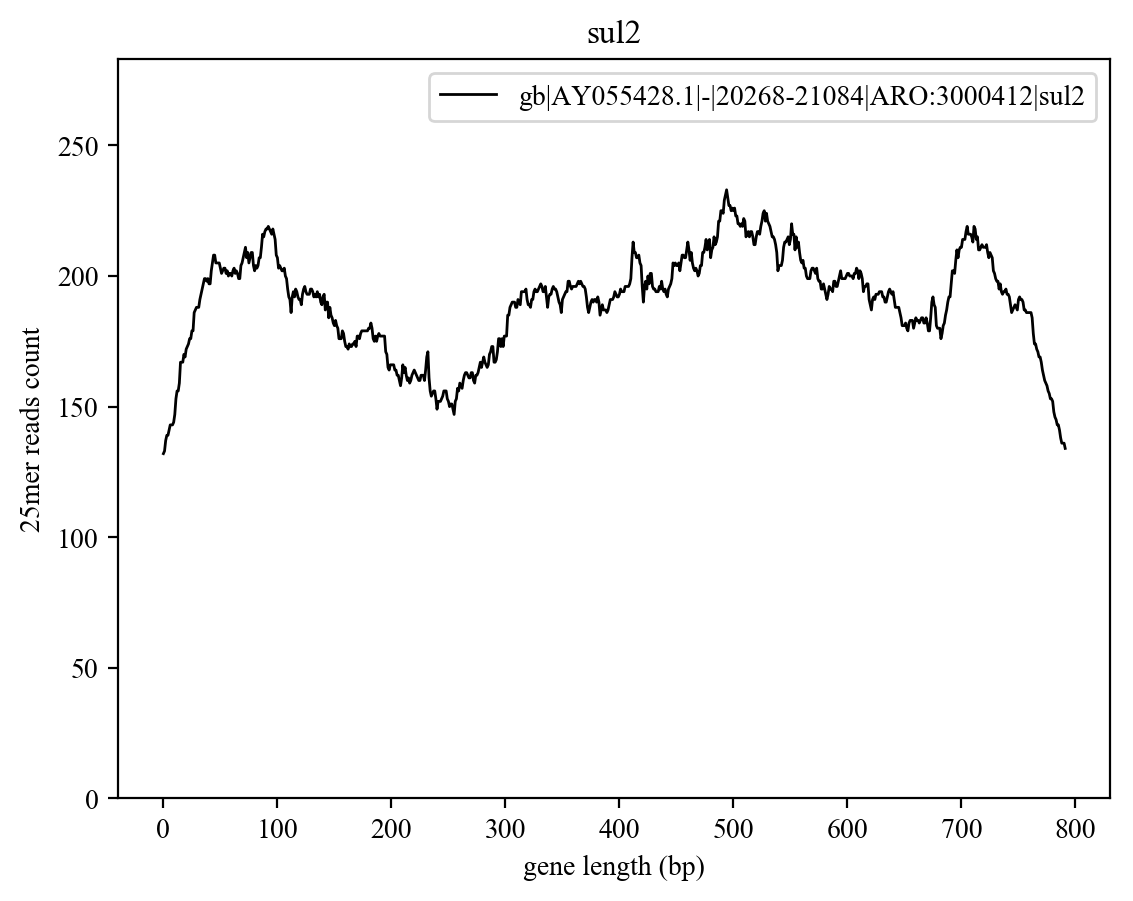

Supplement: Supplementary file 2 — Additional file 2. Archive containing files for evaluation k-mer performance and scoring generated by the k-mer method. [file 12859_2019_3335_MOESM2_ESM.zip › kmer/SJTUF10236_Typhimurium/ar_nucl_25/sul2_25mer.png]

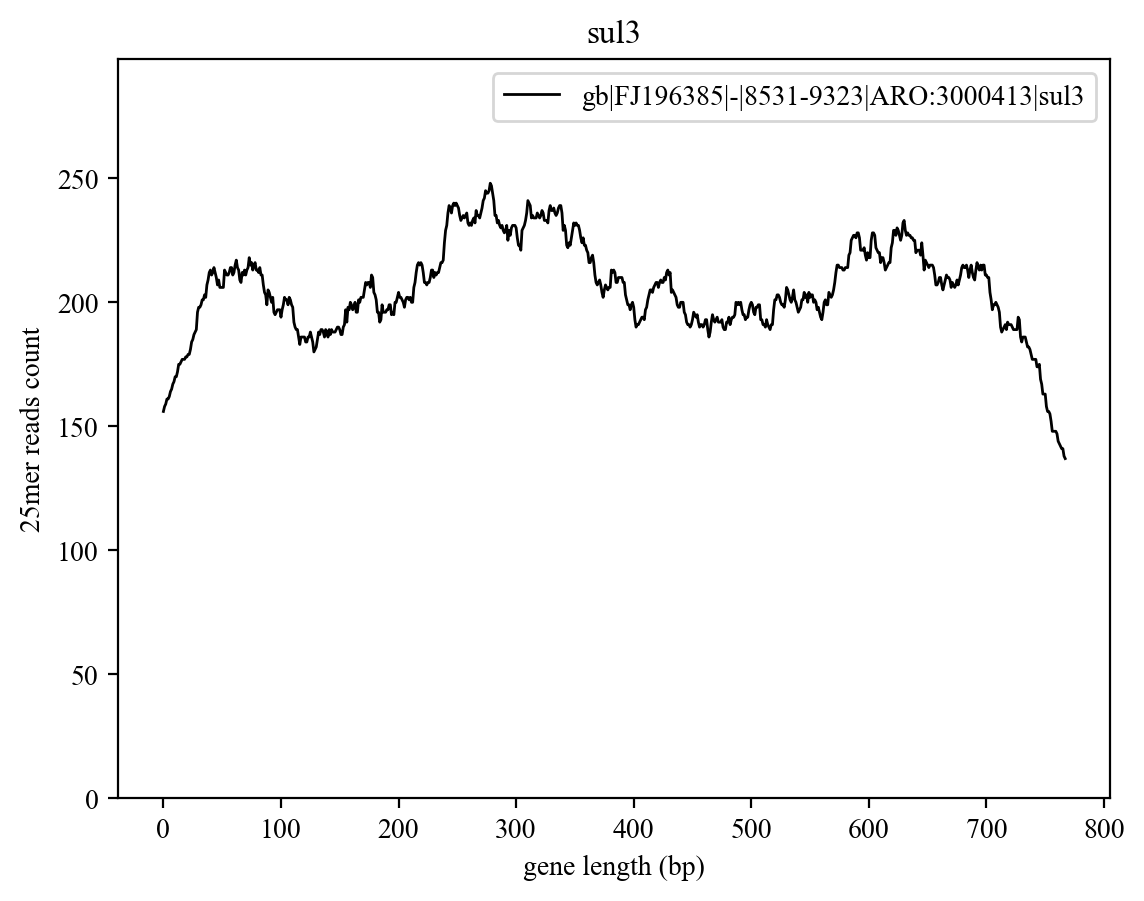

Supplement: Supplementary file 2 — Additional file 2. Archive containing files for evaluation k-mer performance and scoring generated by the k-mer method. [file 12859_2019_3335_MOESM2_ESM.zip › kmer/SJTUF10236_Typhimurium/ar_nucl_25/sul3_25mer.png]

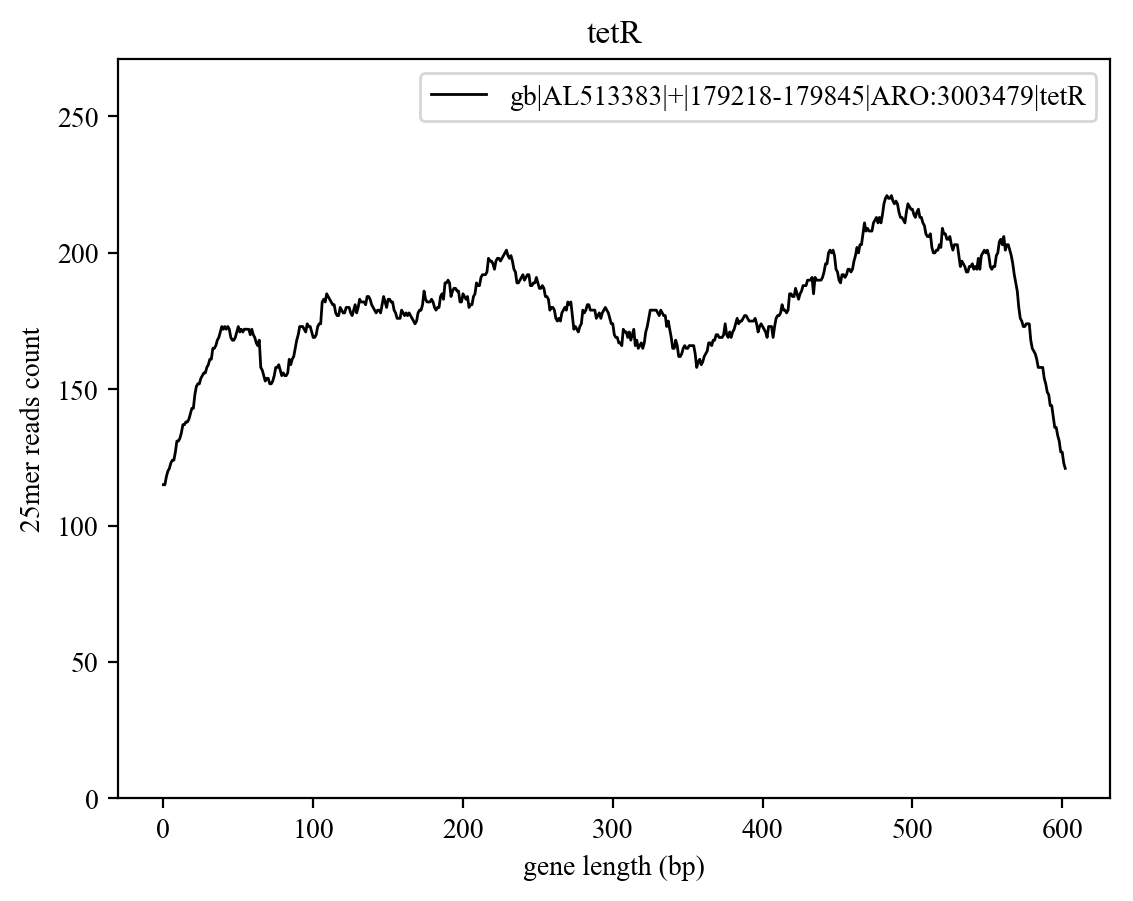

Supplement: Supplementary file 2 — Additional file 2. Archive containing files for evaluation k-mer performance and scoring generated by the k-mer method. [file 12859_2019_3335_MOESM2_ESM.zip › kmer/SJTUF10236_Typhimurium/ar_nucl_25/tetR_25mer.png]

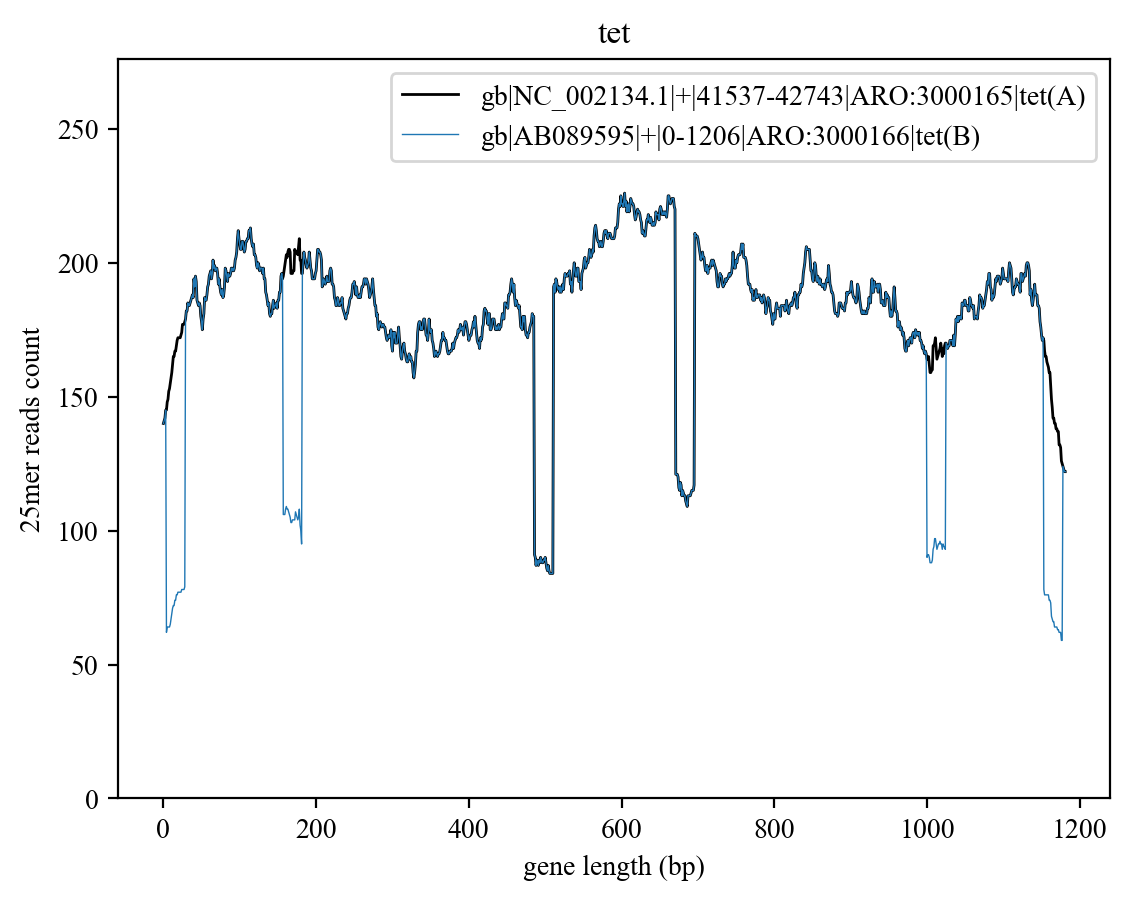

Supplement: Supplementary file 2 — Additional file 2. Archive containing files for evaluation k-mer performance and scoring generated by the k-mer method. [file 12859_2019_3335_MOESM2_ESM.zip › kmer/SJTUF10236_Typhimurium/ar_nucl_25/tet_25mer.png]

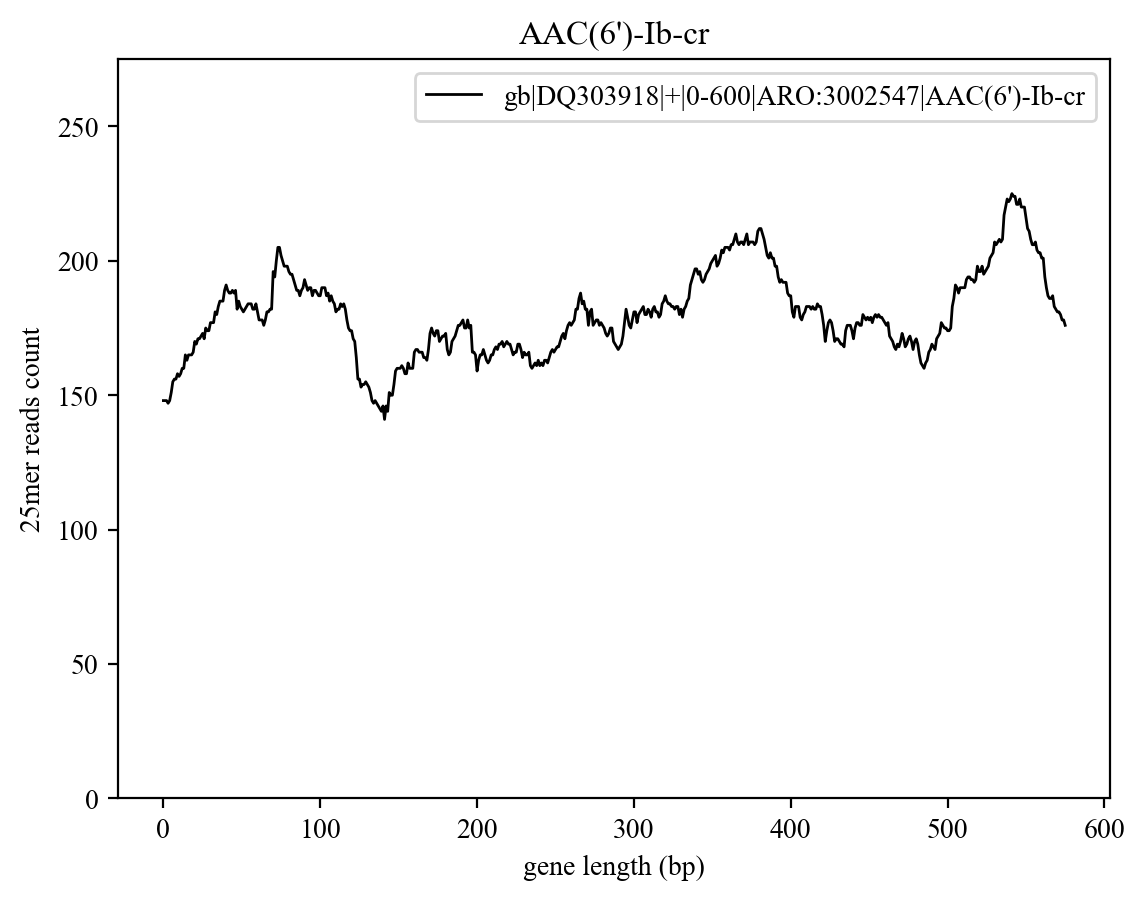

Supplement: Supplementary file 2 — Additional file 2. Archive containing files for evaluation k-mer performance and scoring generated by the k-mer method. [file 12859_2019_3335_MOESM2_ESM.zip › kmer/SJTUF10250_Typhimurium/ar_nucl_25/AAC(6')-Ib-cr_25mer.png]

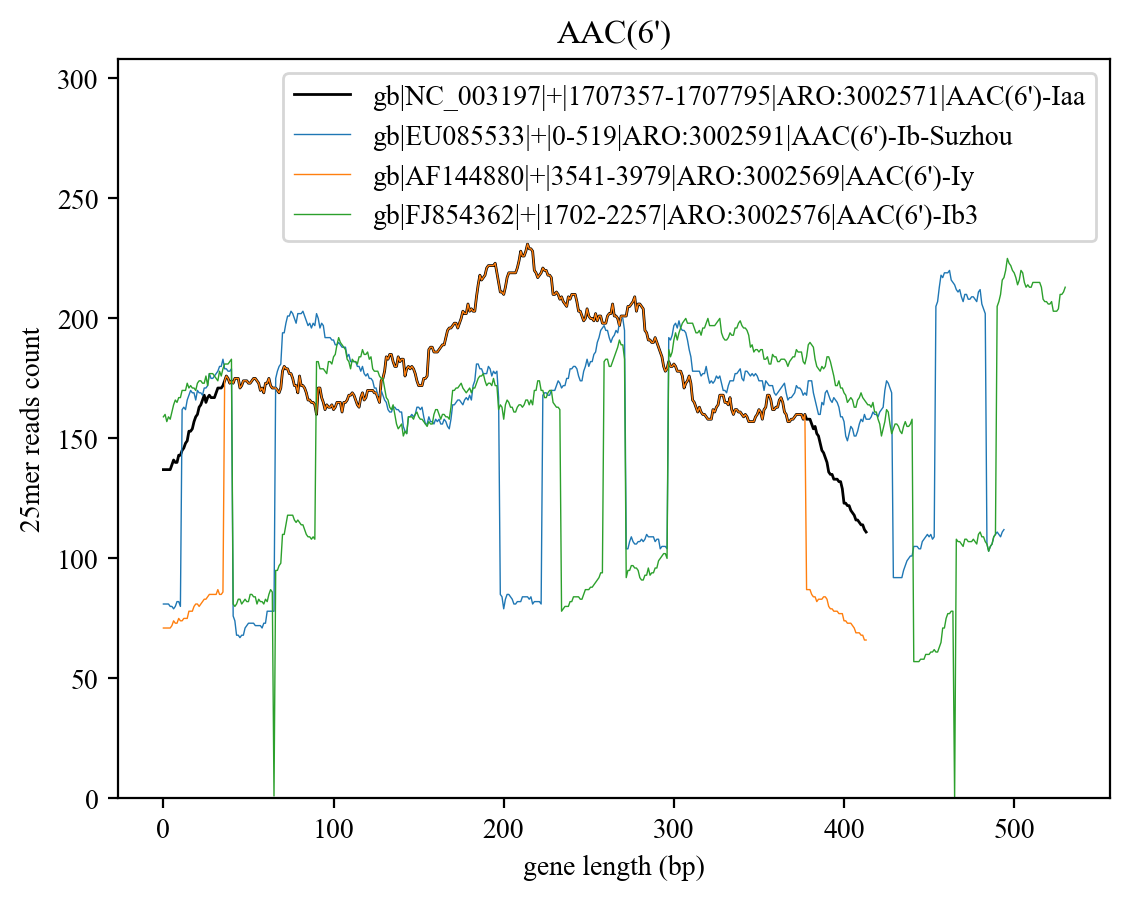

Supplement: Supplementary file 2 — Additional file 2. Archive containing files for evaluation k-mer performance and scoring generated by the k-mer method. [file 12859_2019_3335_MOESM2_ESM.zip › kmer/SJTUF10250_Typhimurium/ar_nucl_25/AAC(6')_25mer.png]

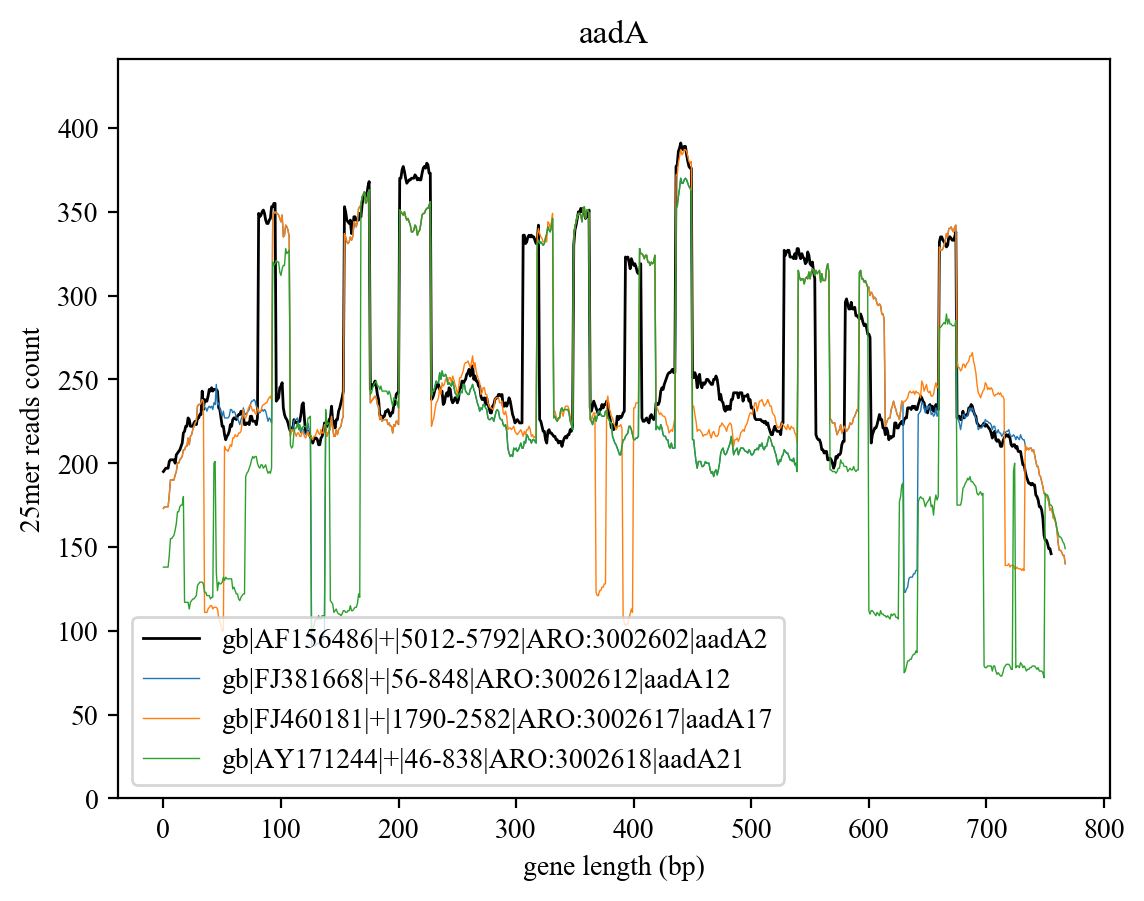

Supplement: Supplementary file 2 — Additional file 2. Archive containing files for evaluation k-mer performance and scoring generated by the k-mer method. [file 12859_2019_3335_MOESM2_ESM.zip › kmer/SJTUF10250_Typhimurium/ar_nucl_25/aadA_25mer.png]

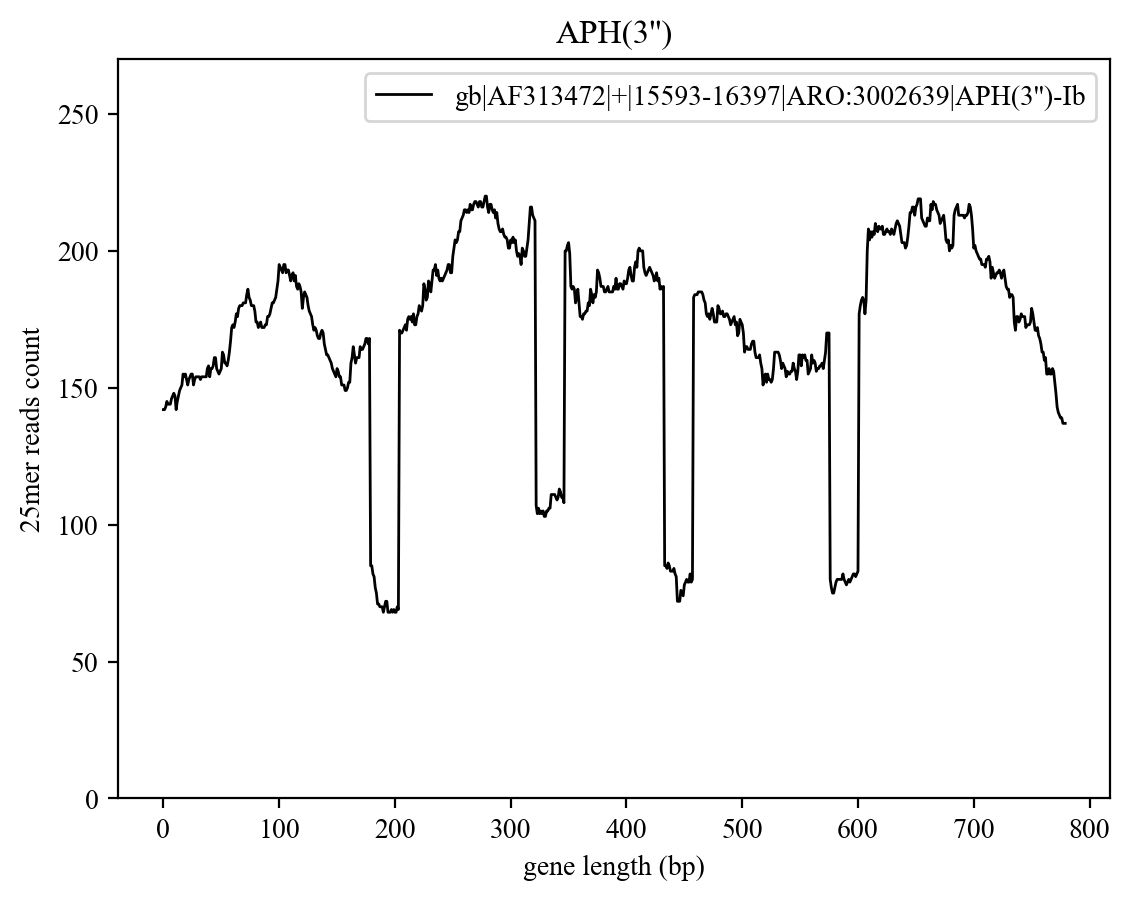

Supplement: Supplementary file 2 — Additional file 2. Archive containing files for evaluation k-mer performance and scoring generated by the k-mer method. [file 12859_2019_3335_MOESM2_ESM.zip › kmer/SJTUF10250_Typhimurium/ar_nucl_25/APH(3'')_25mer.png]

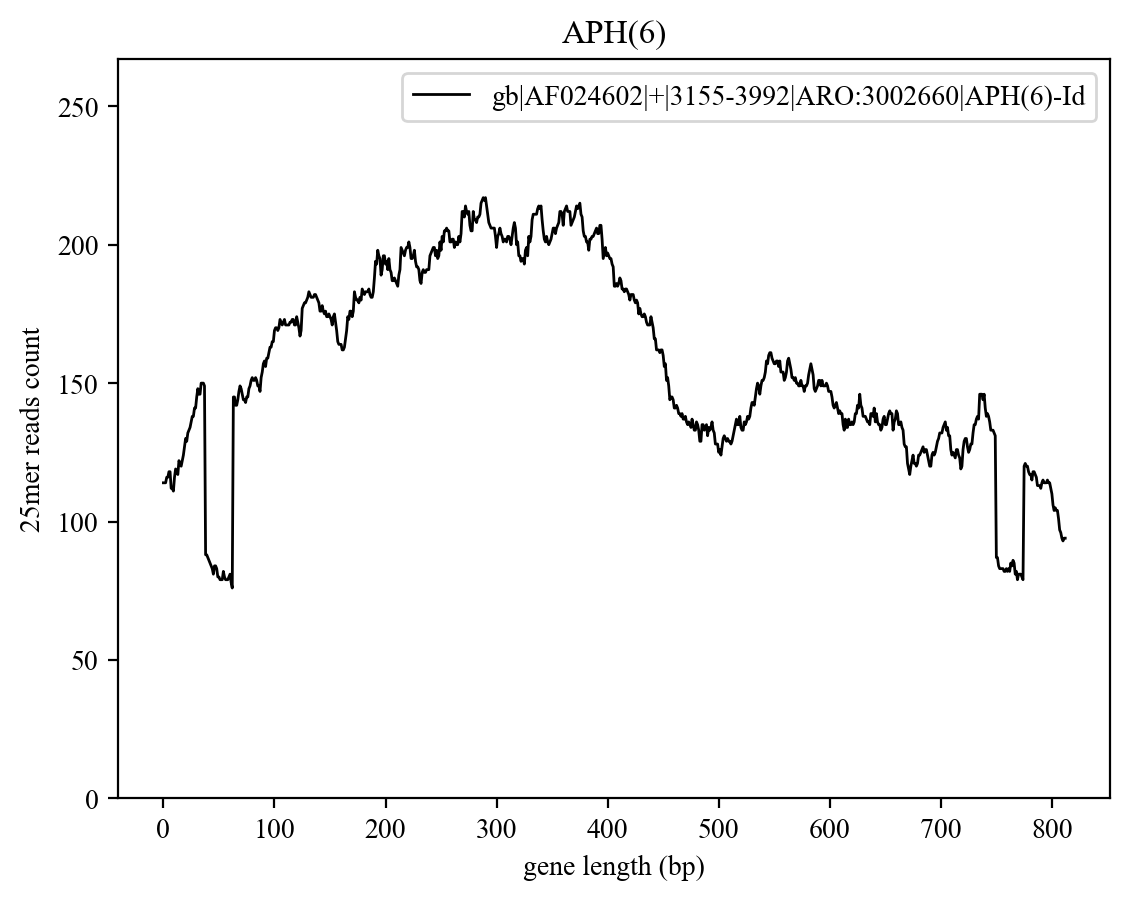

Supplement: Supplementary file 2 — Additional file 2. Archive containing files for evaluation k-mer performance and scoring generated by the k-mer method. [file 12859_2019_3335_MOESM2_ESM.zip › kmer/SJTUF10250_Typhimurium/ar_nucl_25/APH(6)_25mer.png]

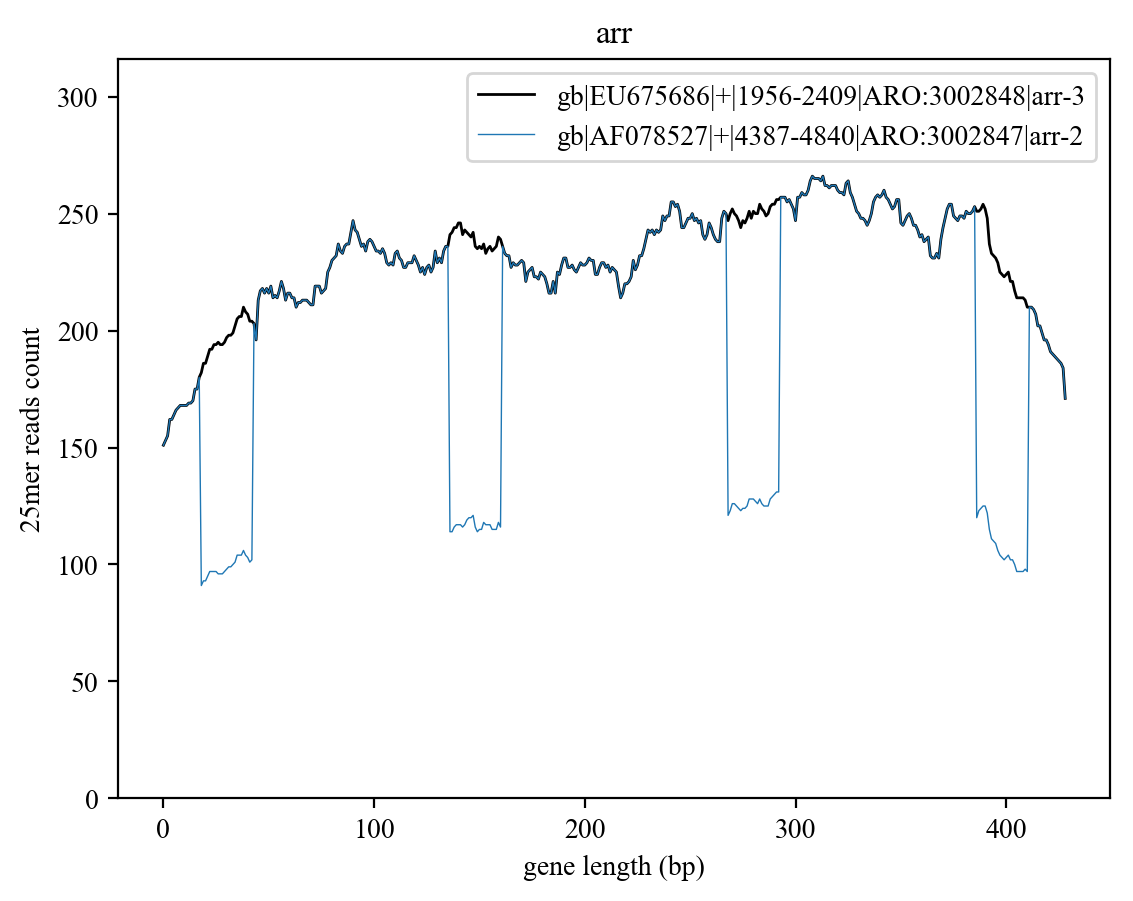

Supplement: Supplementary file 2 — Additional file 2. Archive containing files for evaluation k-mer performance and scoring generated by the k-mer method. [file 12859_2019_3335_MOESM2_ESM.zip › kmer/SJTUF10250_Typhimurium/ar_nucl_25/arr_25mer.png]

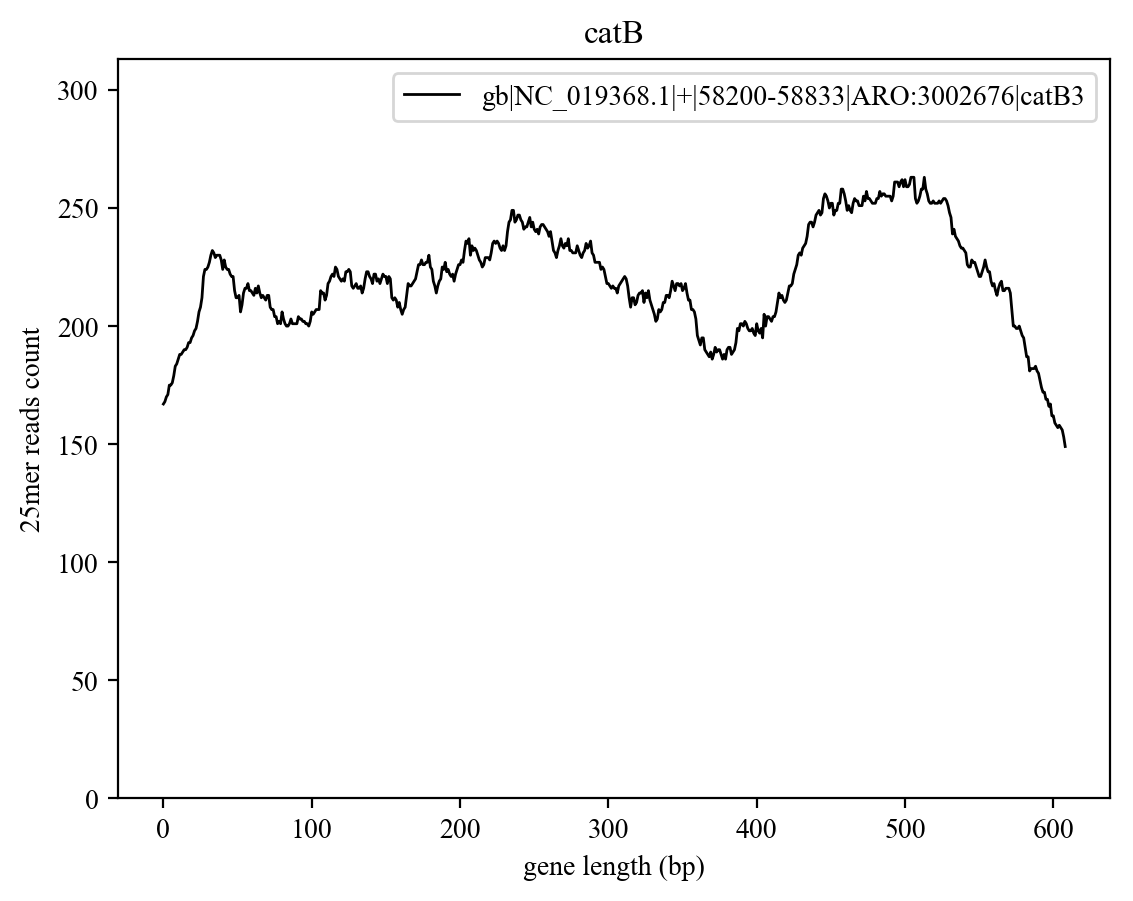

Supplement: Supplementary file 2 — Additional file 2. Archive containing files for evaluation k-mer performance and scoring generated by the k-mer method. [file 12859_2019_3335_MOESM2_ESM.zip › kmer/SJTUF10250_Typhimurium/ar_nucl_25/catB_25mer.png]

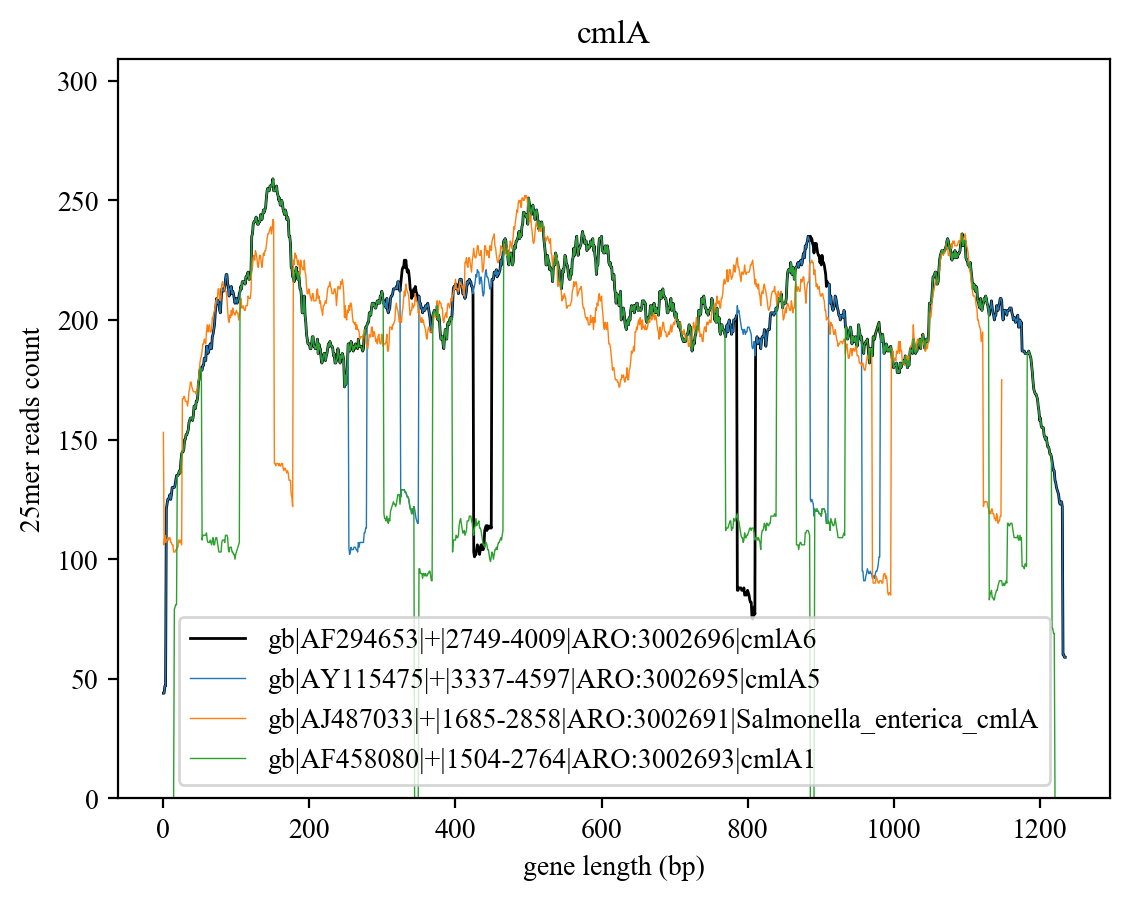

Supplement: Supplementary file 2 — Additional file 2. Archive containing files for evaluation k-mer performance and scoring generated by the k-mer method. [file 12859_2019_3335_MOESM2_ESM.zip › kmer/SJTUF10250_Typhimurium/ar_nucl_25/cmlA_25mer.png]

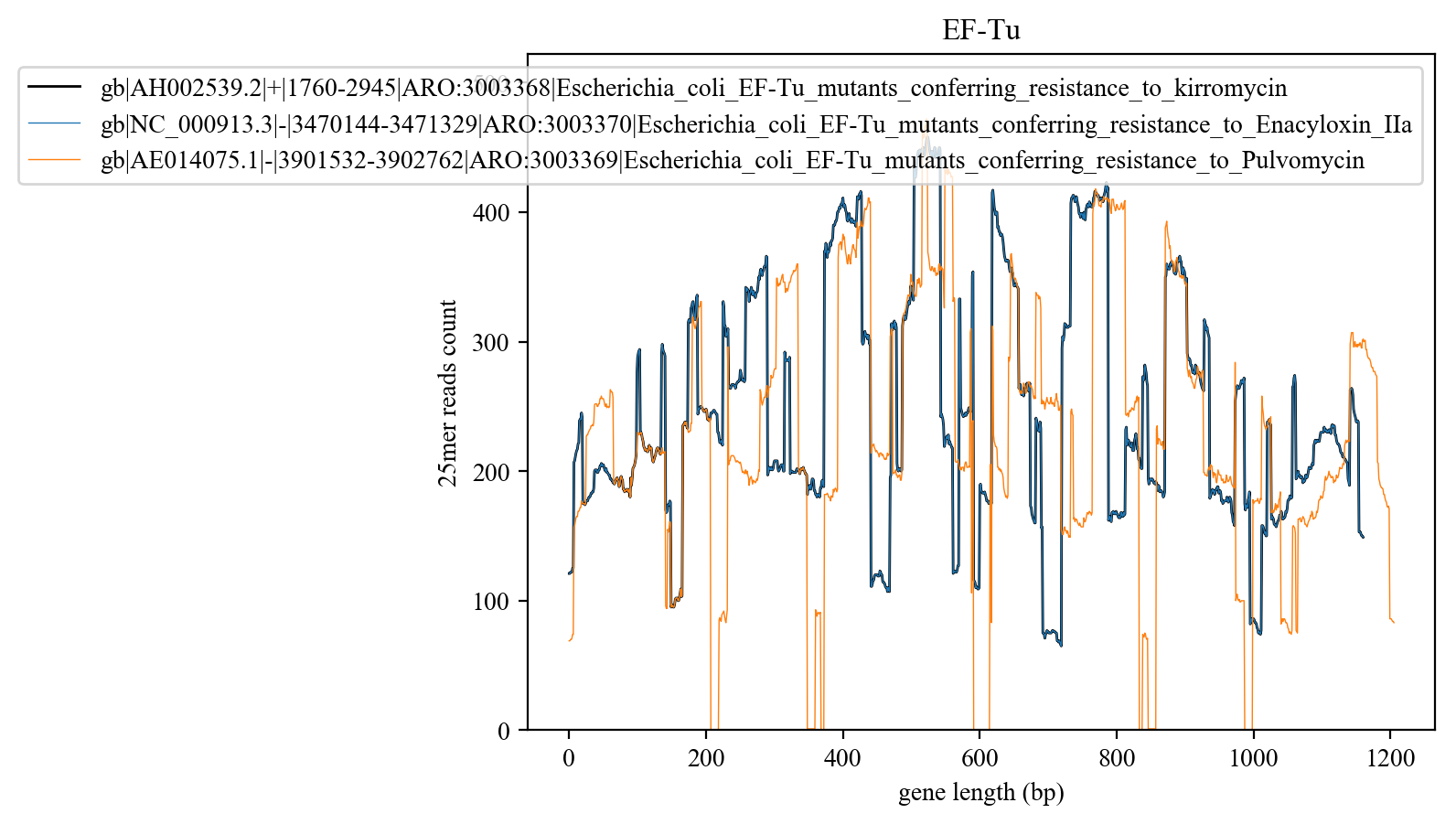

Supplement: Supplementary file 2 — Additional file 2. Archive containing files for evaluation k-mer performance and scoring generated by the k-mer method. [file 12859_2019_3335_MOESM2_ESM.zip › kmer/SJTUF10250_Typhimurium/ar_nucl_25/EF-Tu_25mer.png]

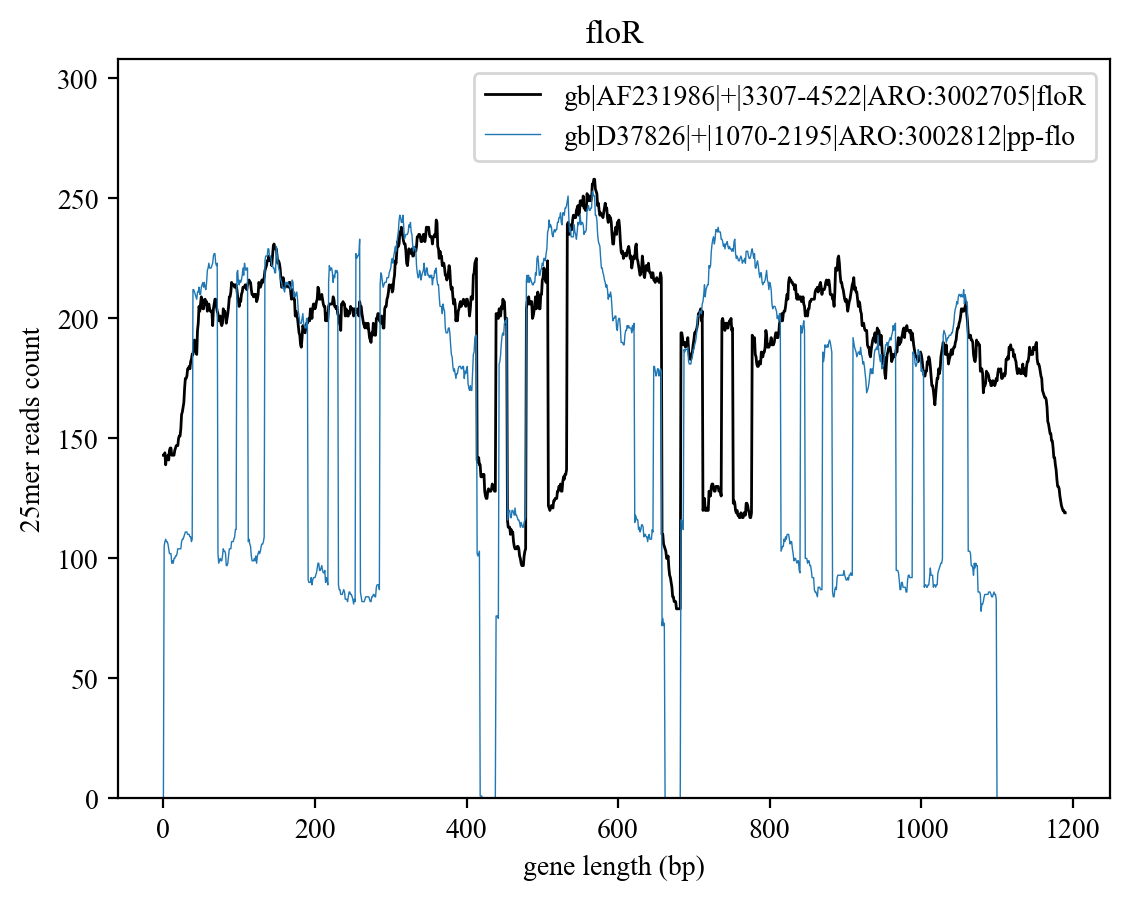

Supplement: Supplementary file 2 — Additional file 2. Archive containing files for evaluation k-mer performance and scoring generated by the k-mer method. [file 12859_2019_3335_MOESM2_ESM.zip › kmer/SJTUF10250_Typhimurium/ar_nucl_25/floR_25mer.png]

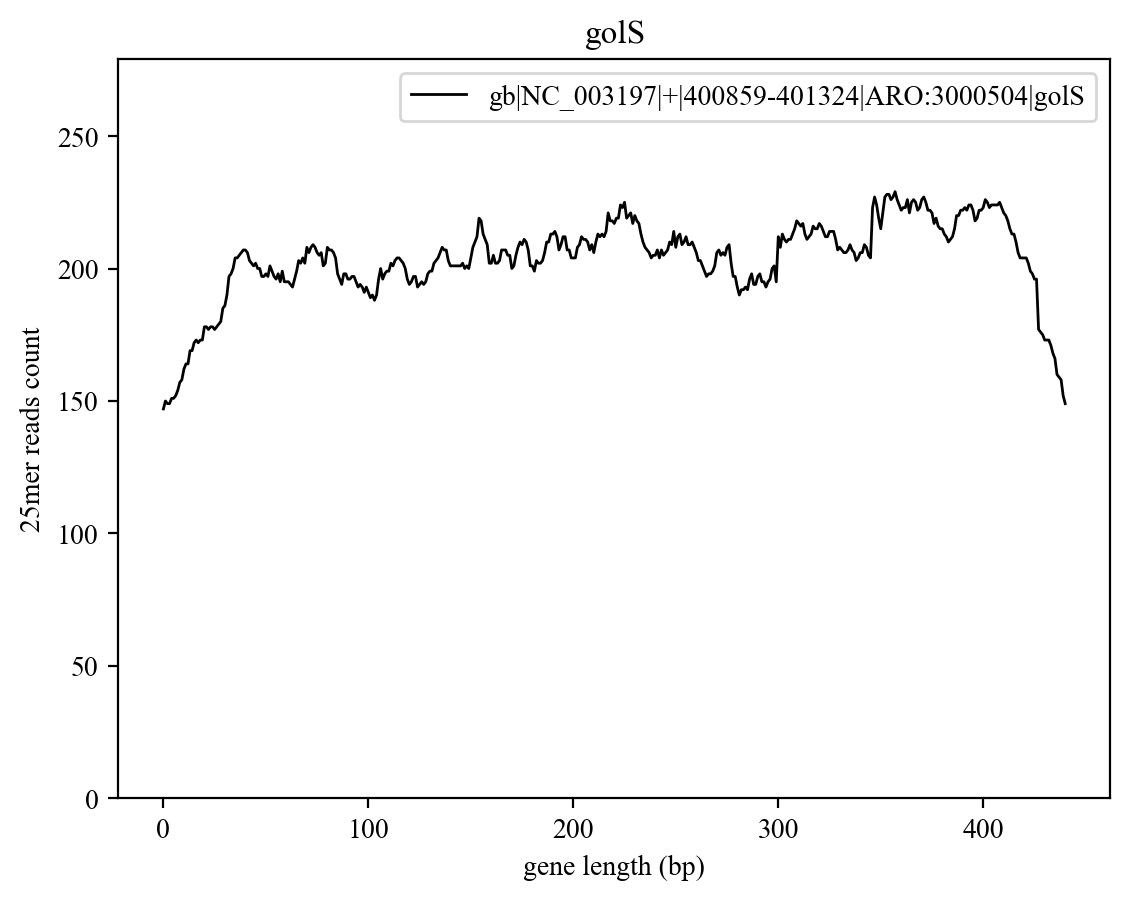

Supplement: Supplementary file 2 — Additional file 2. Archive containing files for evaluation k-mer performance and scoring generated by the k-mer method. [file 12859_2019_3335_MOESM2_ESM.zip › kmer/SJTUF10250_Typhimurium/ar_nucl_25/golS_25mer.png]

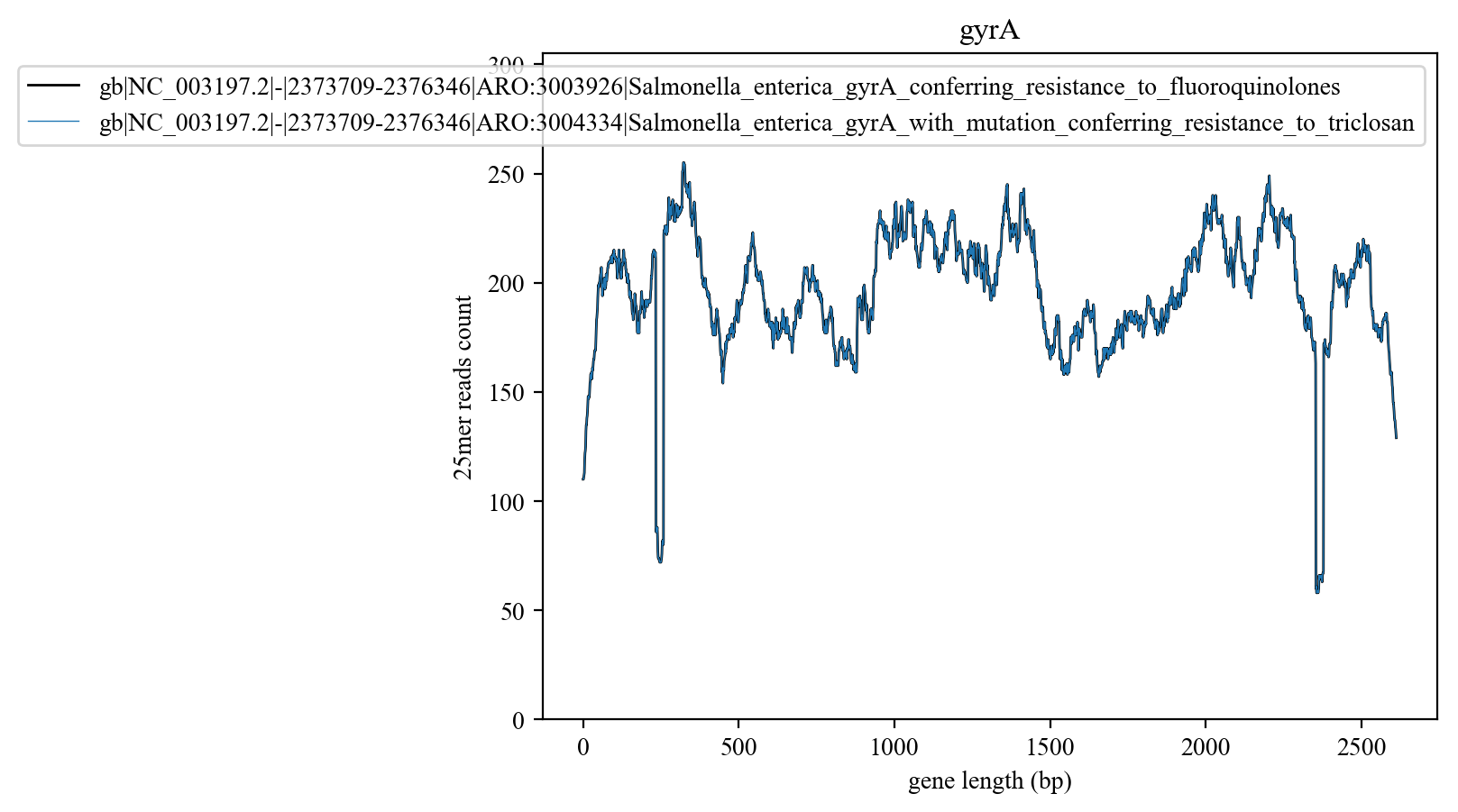

Supplement: Supplementary file 2 — Additional file 2. Archive containing files for evaluation k-mer performance and scoring generated by the k-mer method. [file 12859_2019_3335_MOESM2_ESM.zip › kmer/SJTUF10250_Typhimurium/ar_nucl_25/gyrA_25mer.png]

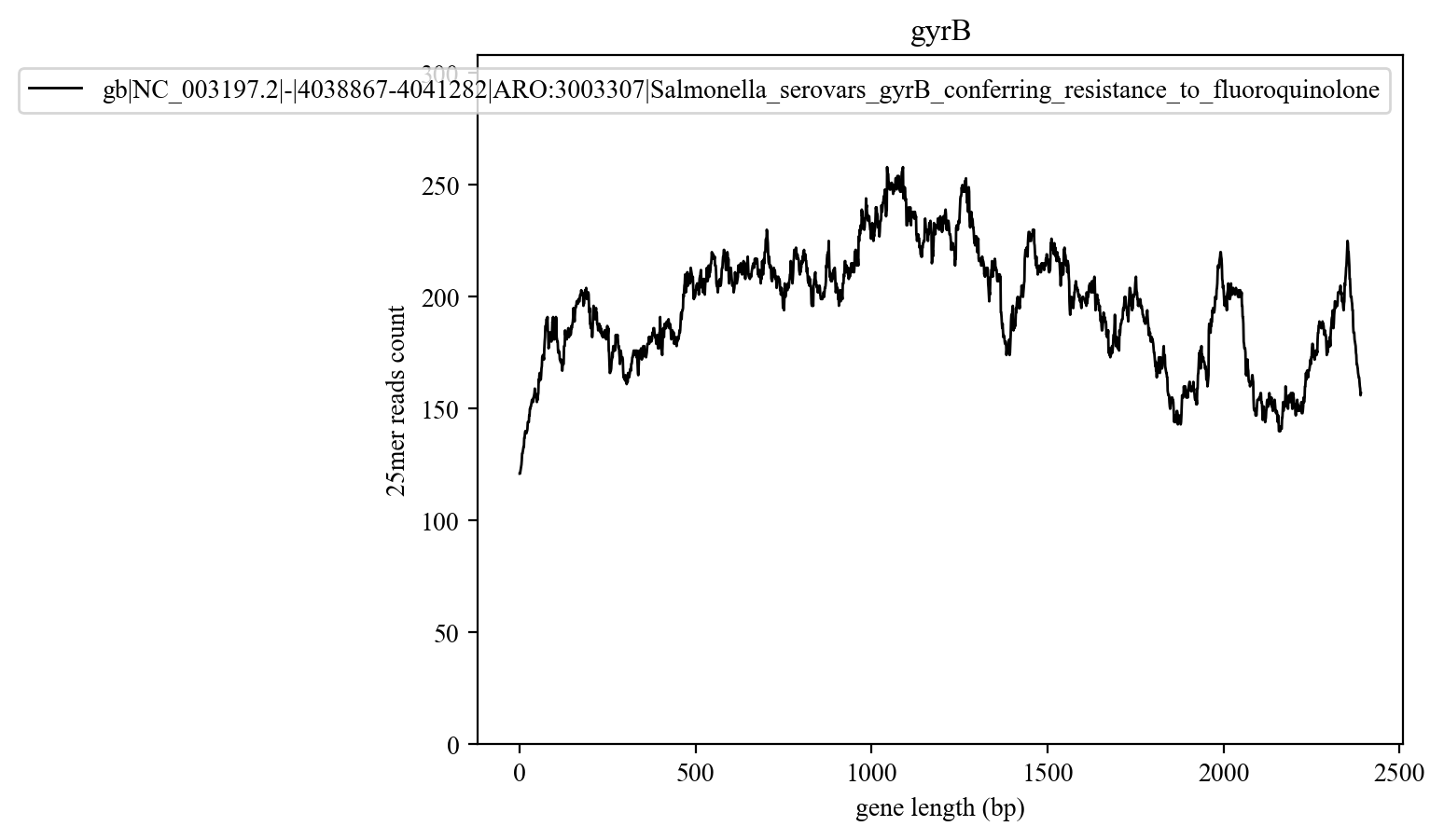

Supplement: Supplementary file 2 — Additional file 2. Archive containing files for evaluation k-mer performance and scoring generated by the k-mer method. [file 12859_2019_3335_MOESM2_ESM.zip › kmer/SJTUF10250_Typhimurium/ar_nucl_25/gyrB_25mer.png]

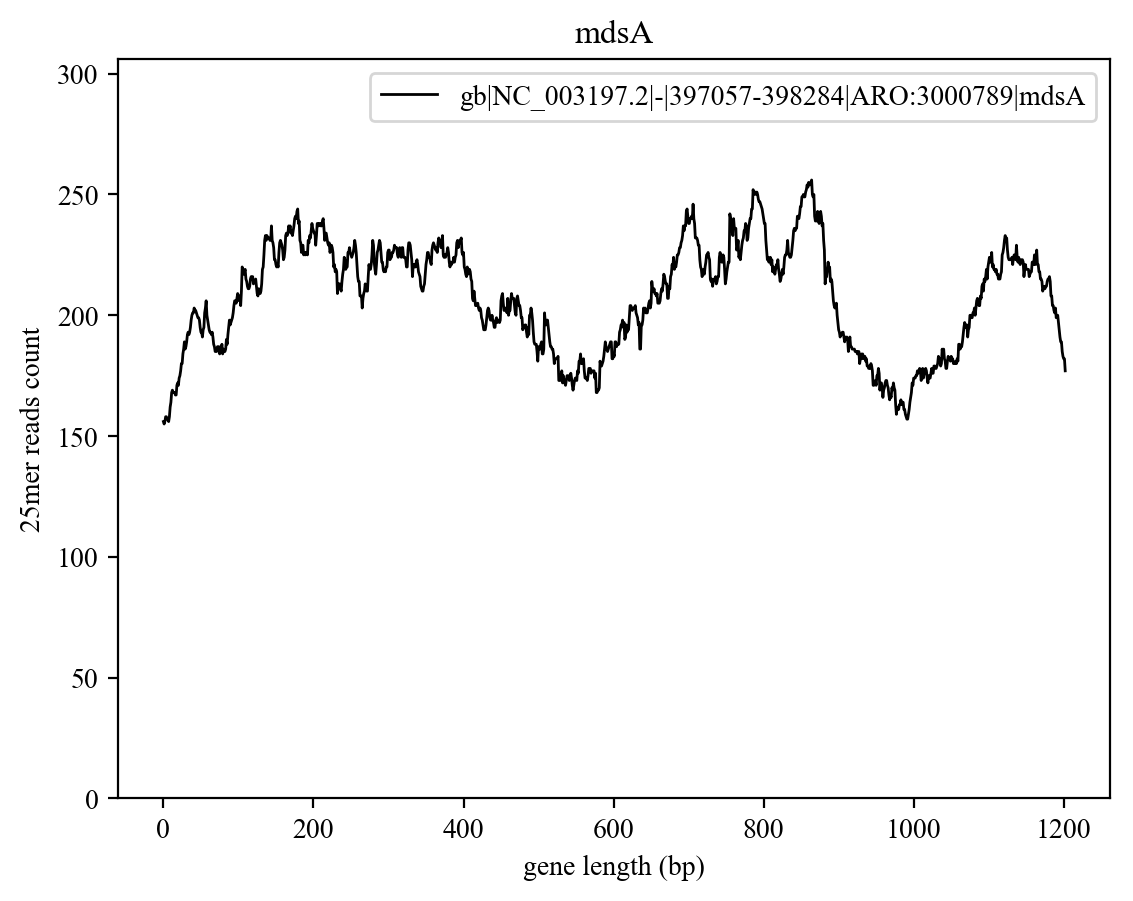

Supplement: Supplementary file 2 — Additional file 2. Archive containing files for evaluation k-mer performance and scoring generated by the k-mer method. [file 12859_2019_3335_MOESM2_ESM.zip › kmer/SJTUF10250_Typhimurium/ar_nucl_25/mdsA_25mer.png]

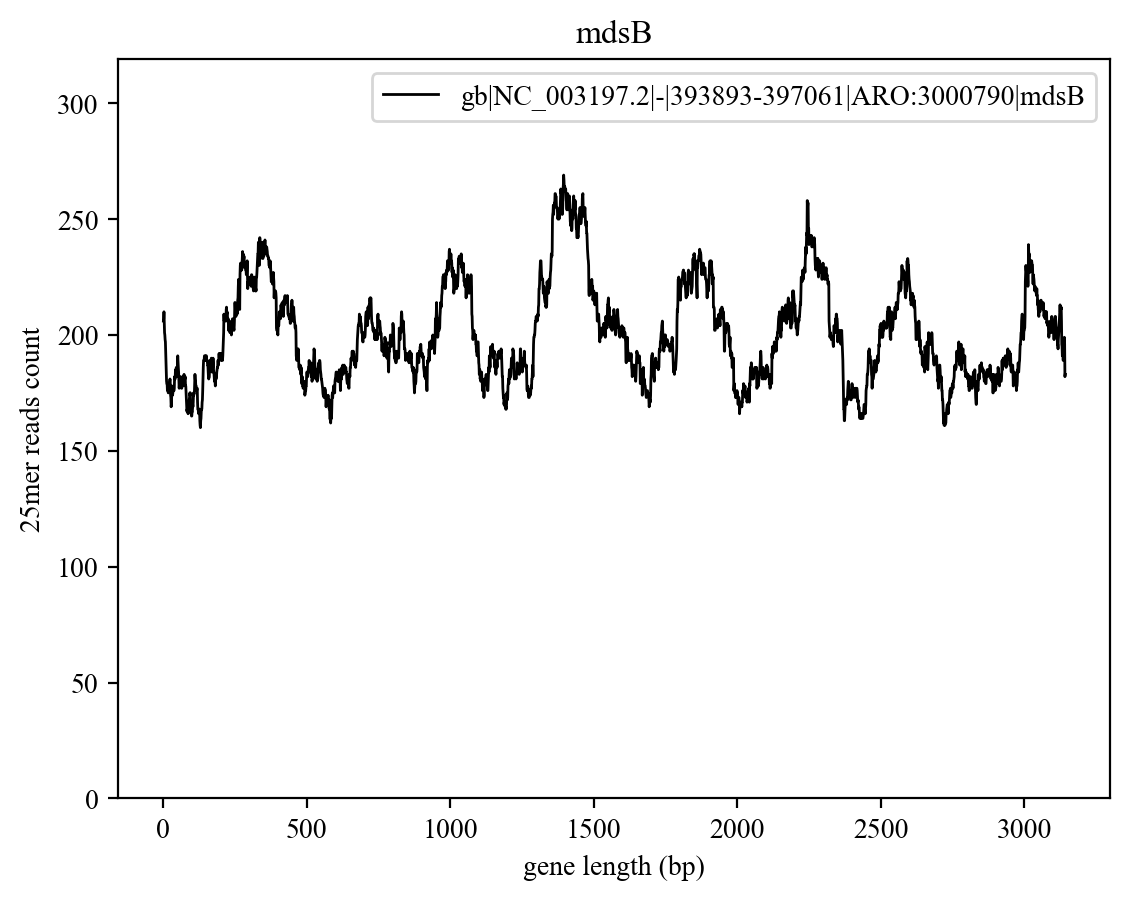

Supplement: Supplementary file 2 — Additional file 2. Archive containing files for evaluation k-mer performance and scoring generated by the k-mer method. [file 12859_2019_3335_MOESM2_ESM.zip › kmer/SJTUF10250_Typhimurium/ar_nucl_25/mdsB_25mer.png]

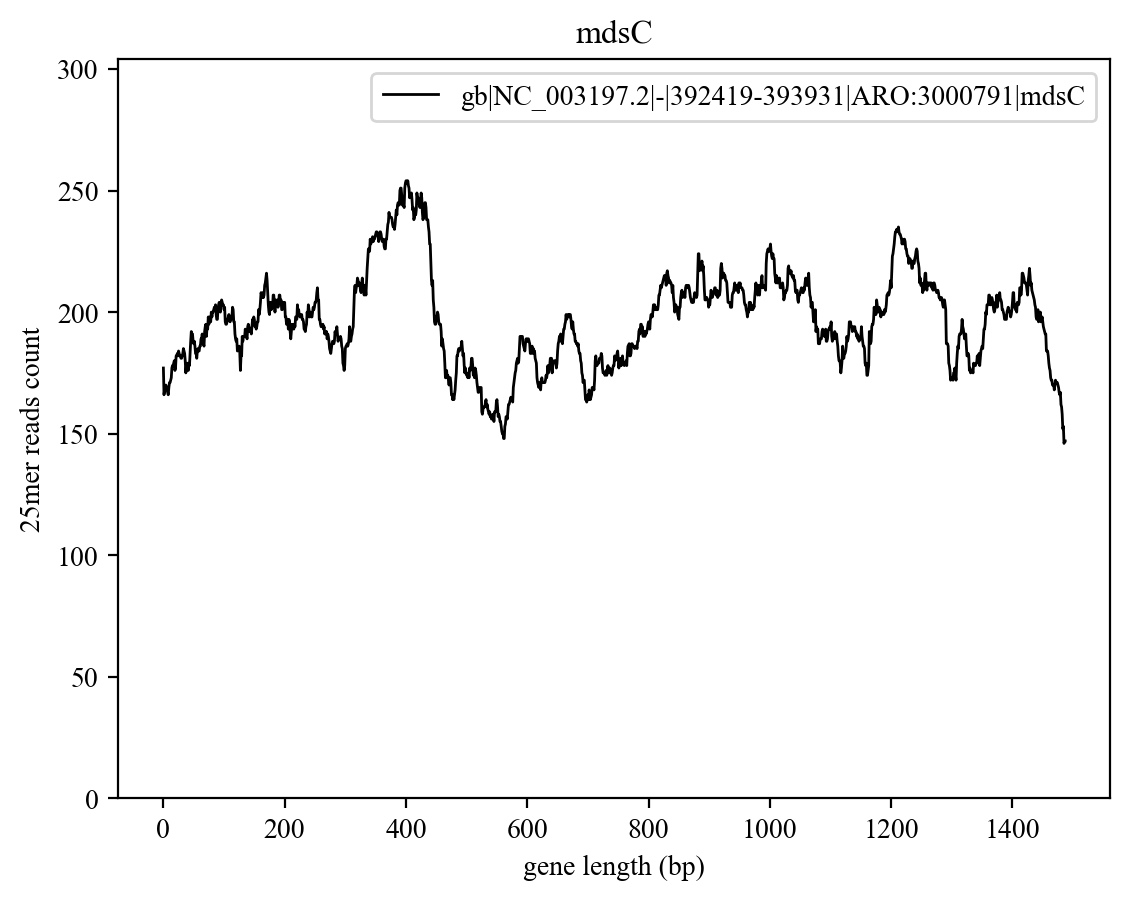

Supplement: Supplementary file 2 — Additional file 2. Archive containing files for evaluation k-mer performance and scoring generated by the k-mer method. [file 12859_2019_3335_MOESM2_ESM.zip › kmer/SJTUF10250_Typhimurium/ar_nucl_25/mdsC_25mer.png]

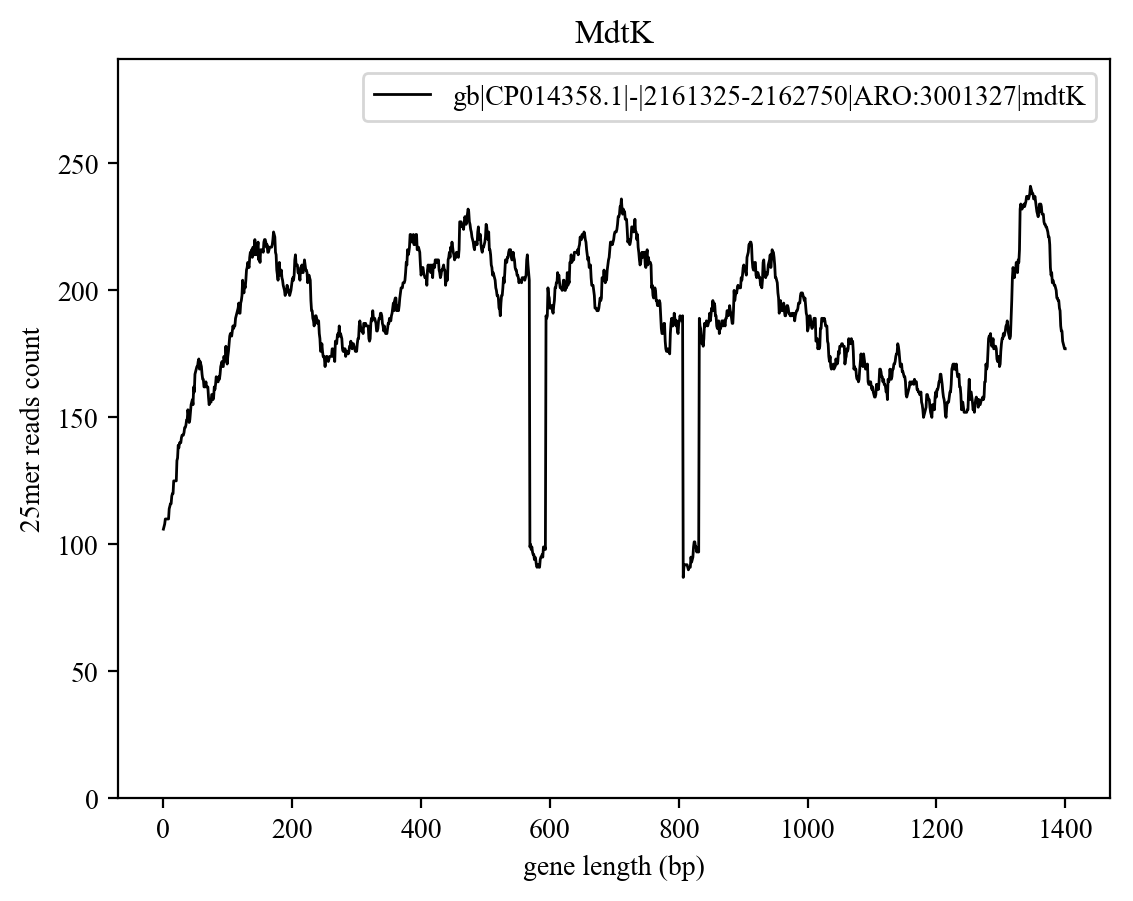

Supplement: Supplementary file 2 — Additional file 2. Archive containing files for evaluation k-mer performance and scoring generated by the k-mer method. [file 12859_2019_3335_MOESM2_ESM.zip › kmer/SJTUF10250_Typhimurium/ar_nucl_25/MdtK_25mer.png]

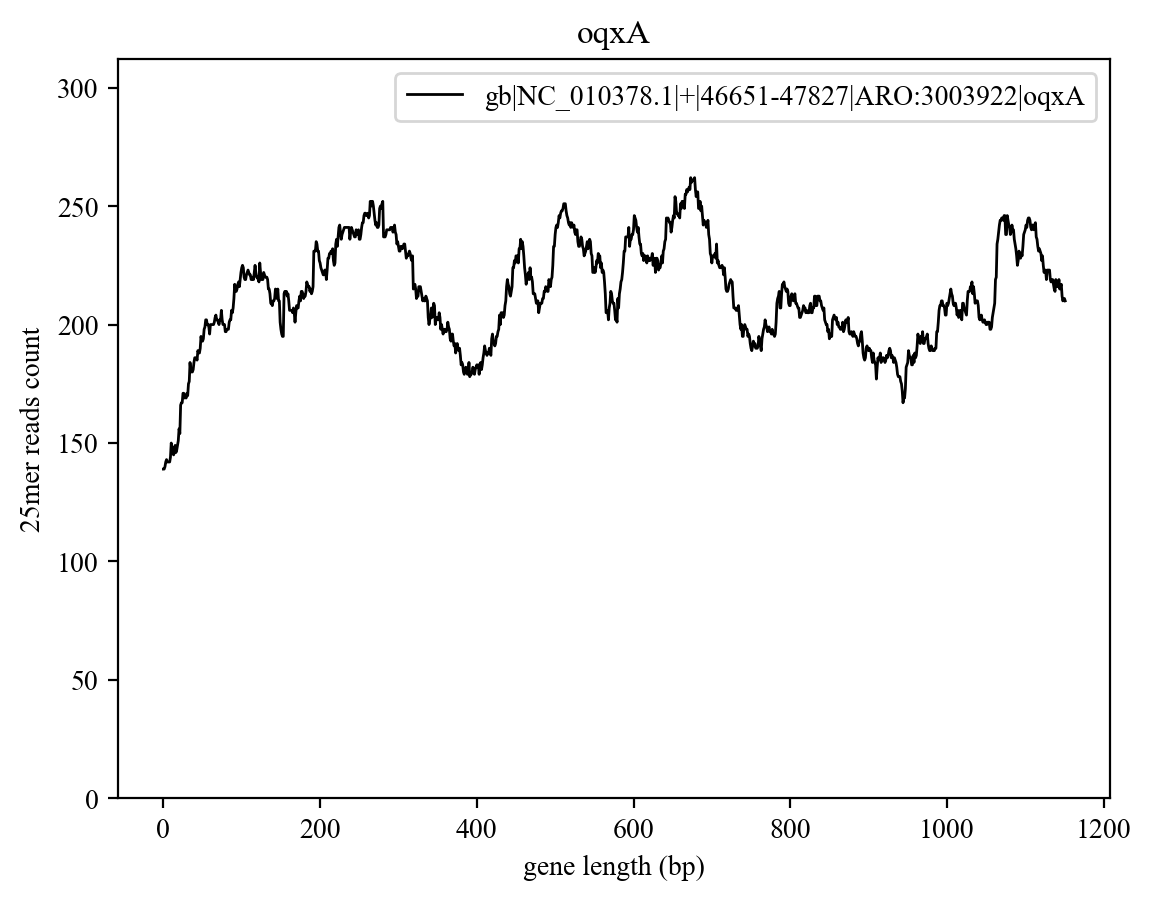

Supplement: Supplementary file 2 — Additional file 2. Archive containing files for evaluation k-mer performance and scoring generated by the k-mer method. [file 12859_2019_3335_MOESM2_ESM.zip › kmer/SJTUF10250_Typhimurium/ar_nucl_25/oqxA_25mer.png]

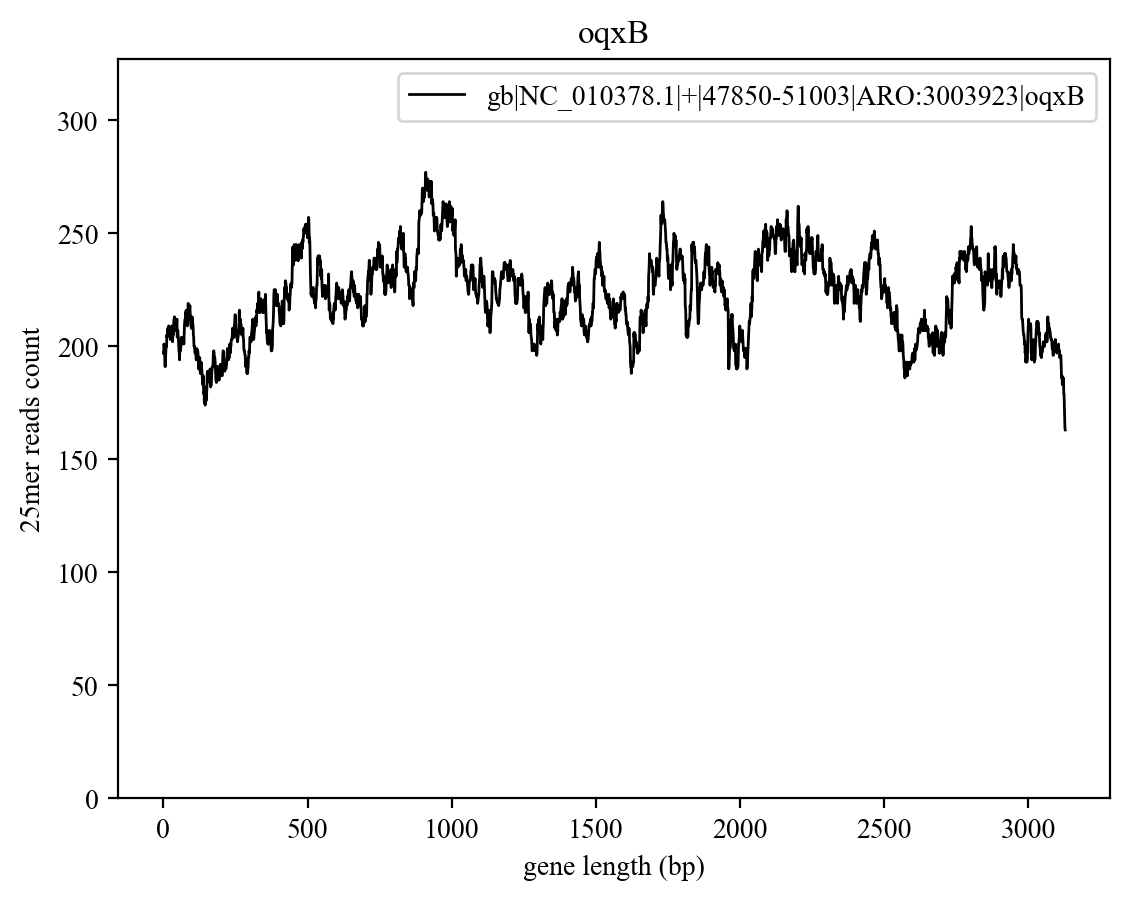

Supplement: Supplementary file 2 — Additional file 2. Archive containing files for evaluation k-mer performance and scoring generated by the k-mer method. [file 12859_2019_3335_MOESM2_ESM.zip › kmer/SJTUF10250_Typhimurium/ar_nucl_25/oqxB_25mer.png]

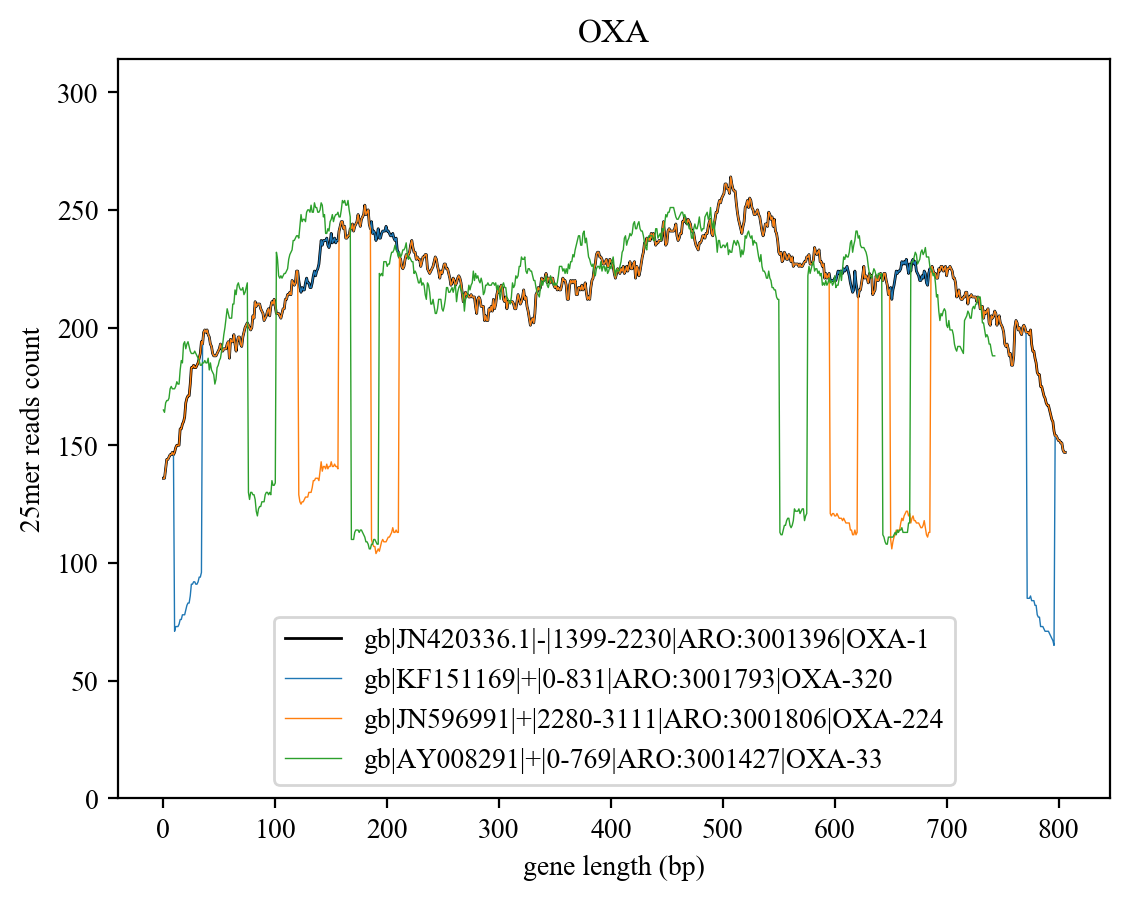

Supplement: Supplementary file 2 — Additional file 2. Archive containing files for evaluation k-mer performance and scoring generated by the k-mer method. [file 12859_2019_3335_MOESM2_ESM.zip › kmer/SJTUF10250_Typhimurium/ar_nucl_25/OXA_25mer.png]

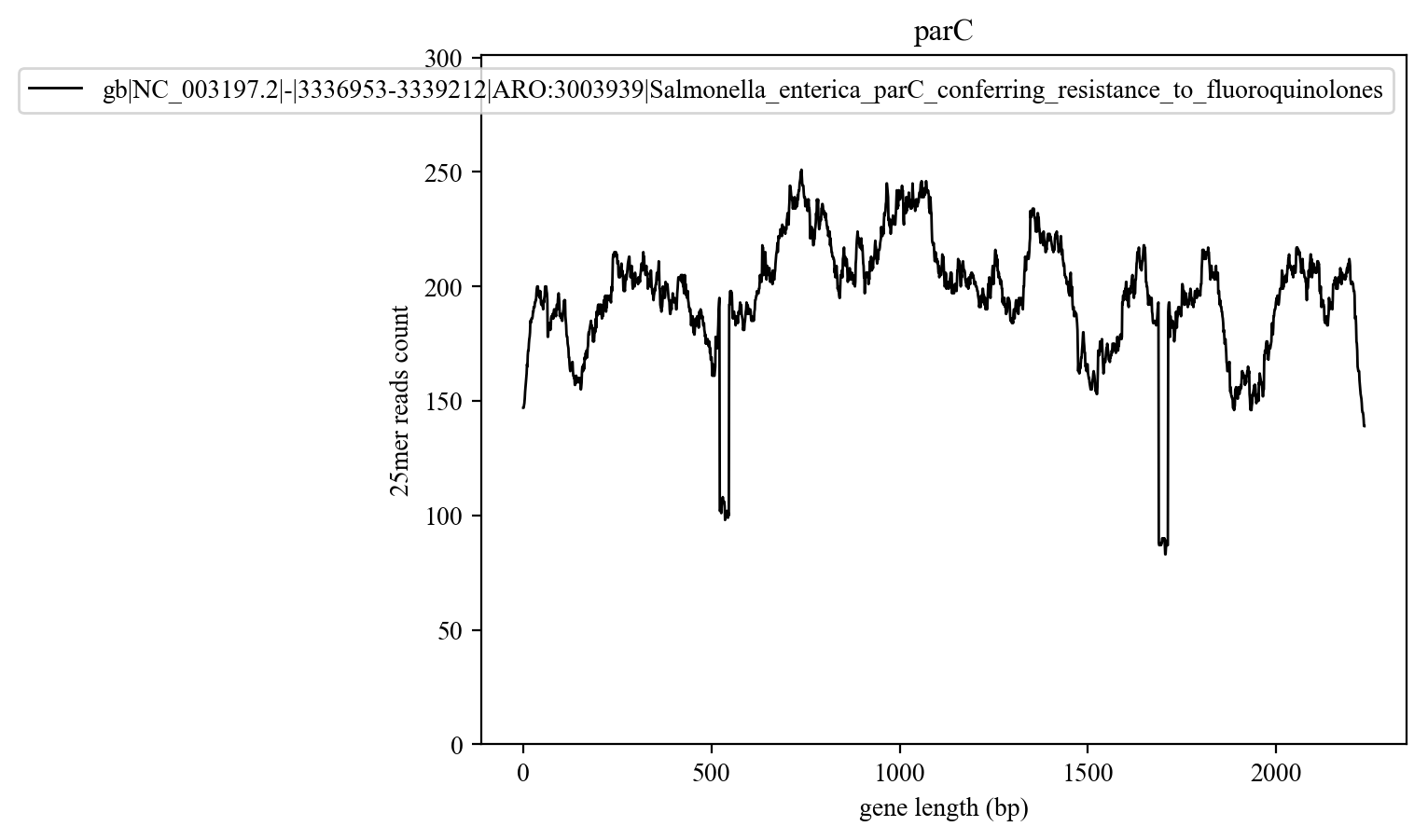

Supplement: Supplementary file 2 — Additional file 2. Archive containing files for evaluation k-mer performance and scoring generated by the k-mer method. [file 12859_2019_3335_MOESM2_ESM.zip › kmer/SJTUF10250_Typhimurium/ar_nucl_25/parC_25mer.png]

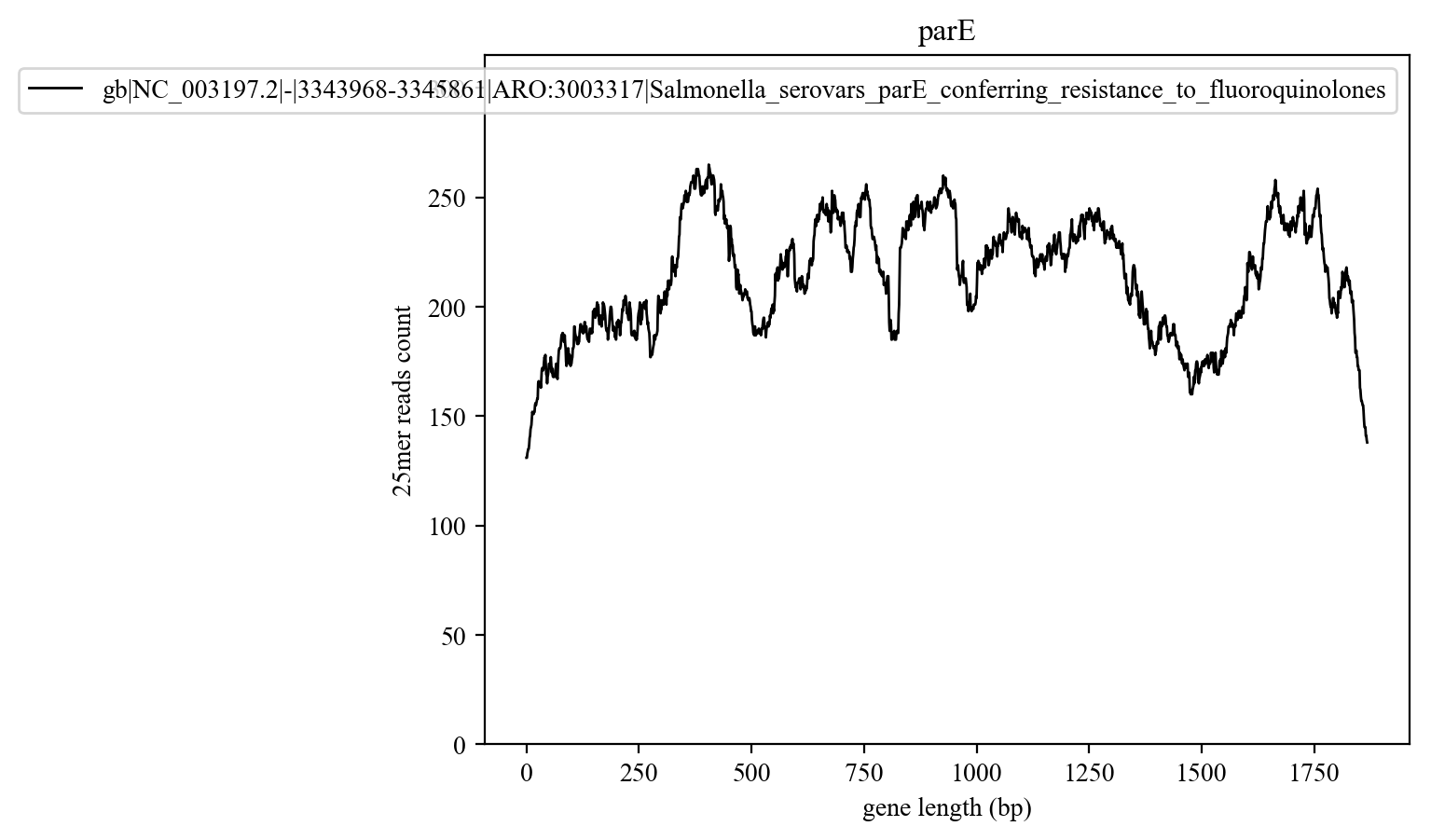

Supplement: Supplementary file 2 — Additional file 2. Archive containing files for evaluation k-mer performance and scoring generated by the k-mer method. [file 12859_2019_3335_MOESM2_ESM.zip › kmer/SJTUF10250_Typhimurium/ar_nucl_25/parE_25mer.png]

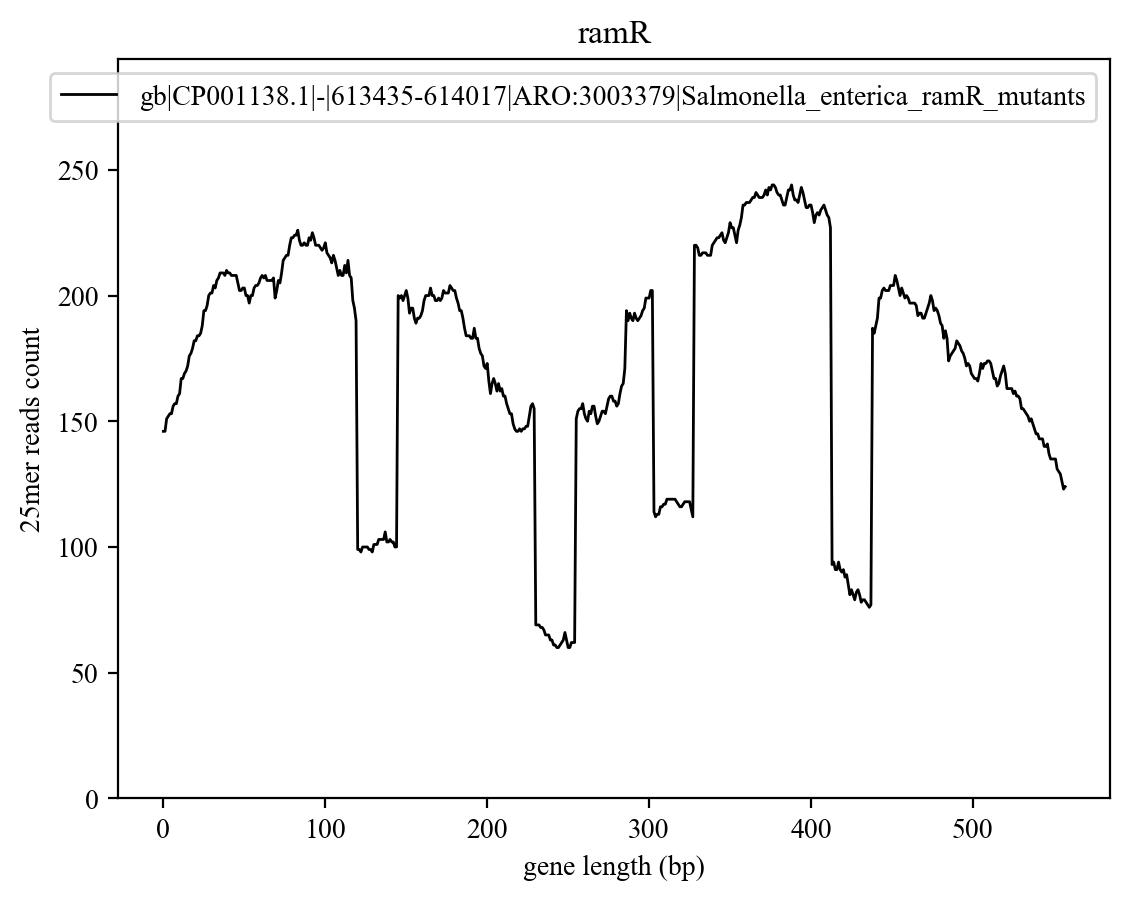

Supplement: Supplementary file 2 — Additional file 2. Archive containing files for evaluation k-mer performance and scoring generated by the k-mer method. [file 12859_2019_3335_MOESM2_ESM.zip › kmer/SJTUF10250_Typhimurium/ar_nucl_25/ramR_25mer.png]

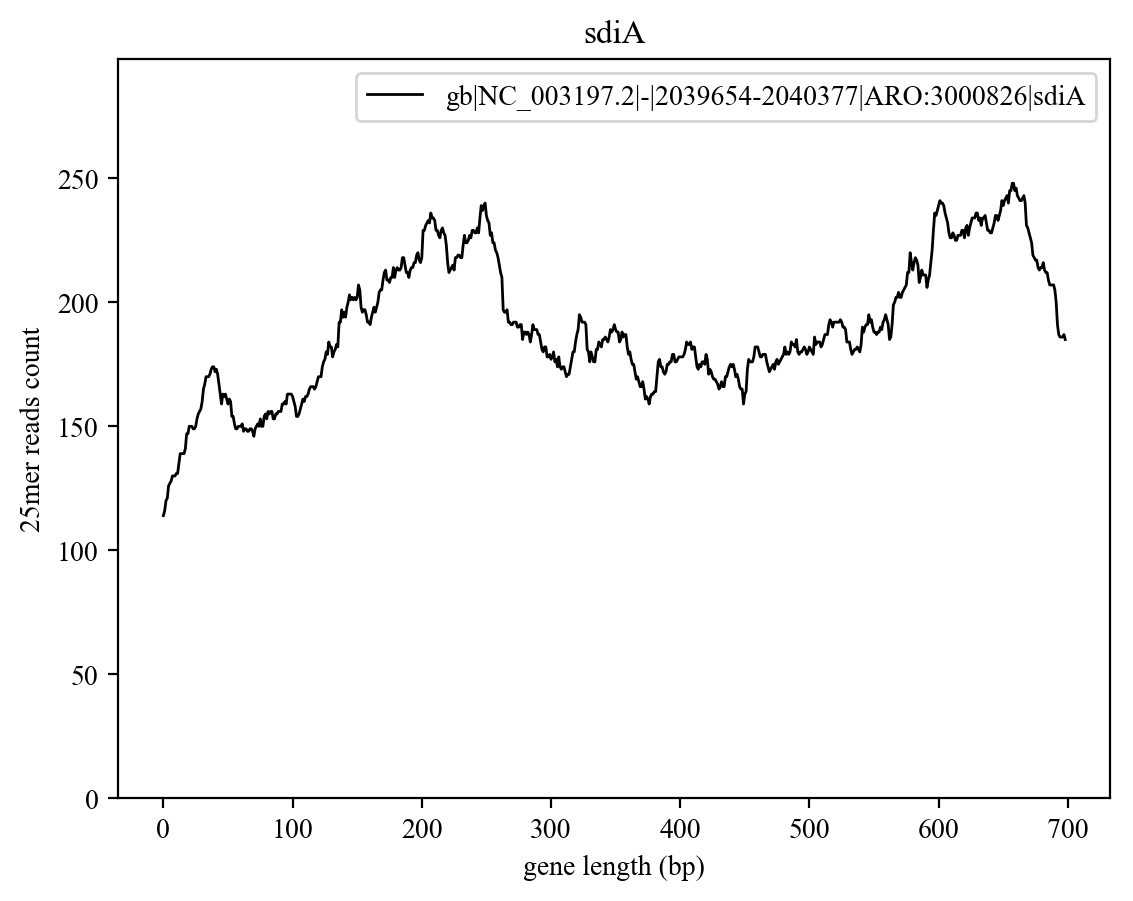

Supplement: Supplementary file 2 — Additional file 2. Archive containing files for evaluation k-mer performance and scoring generated by the k-mer method. [file 12859_2019_3335_MOESM2_ESM.zip › kmer/SJTUF10250_Typhimurium/ar_nucl_25/sdiA_25mer.png]

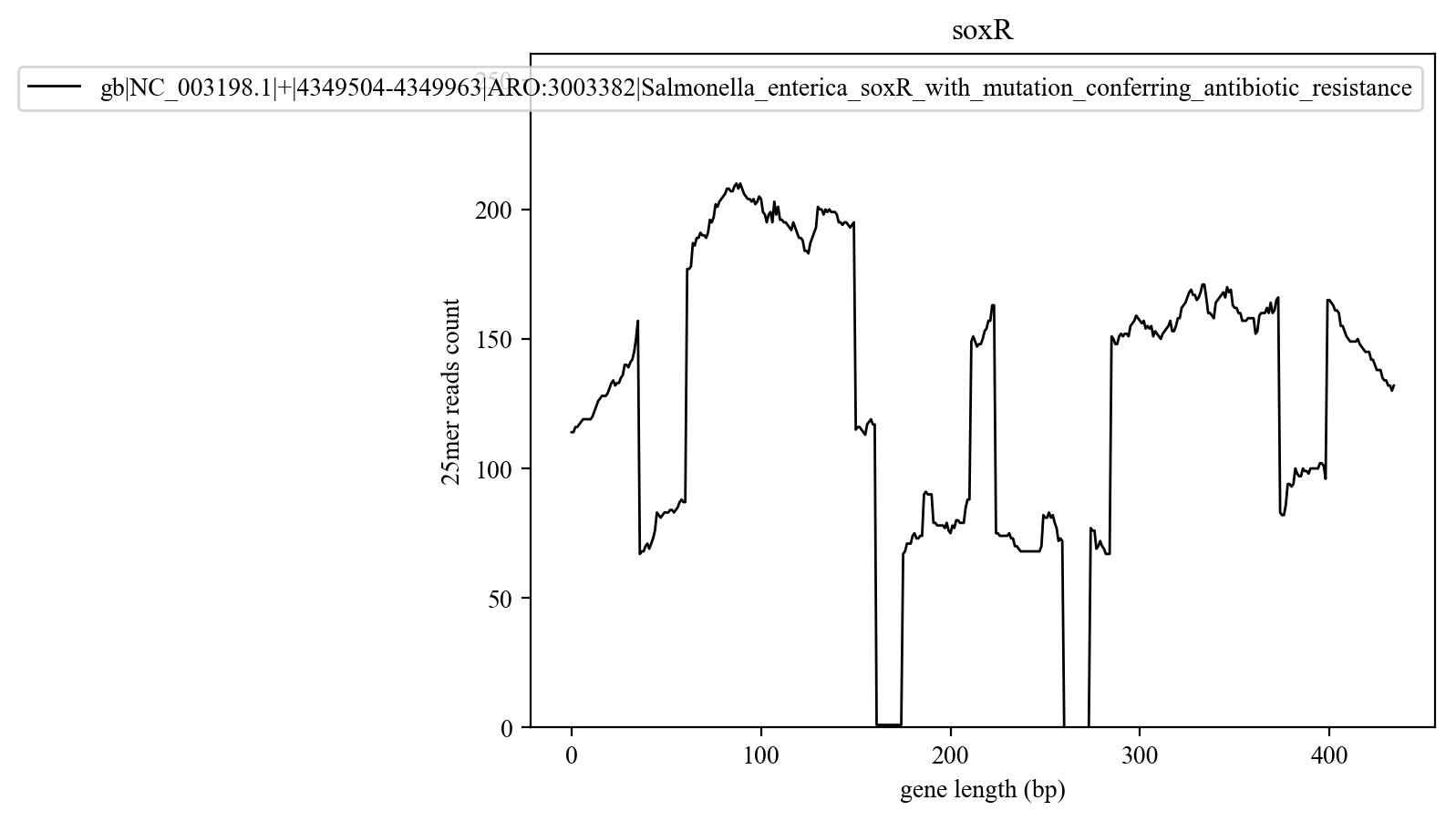

Supplement: Supplementary file 2 — Additional file 2. Archive containing files for evaluation k-mer performance and scoring generated by the k-mer method. [file 12859_2019_3335_MOESM2_ESM.zip › kmer/SJTUF10250_Typhimurium/ar_nucl_25/soxR_25mer.png]

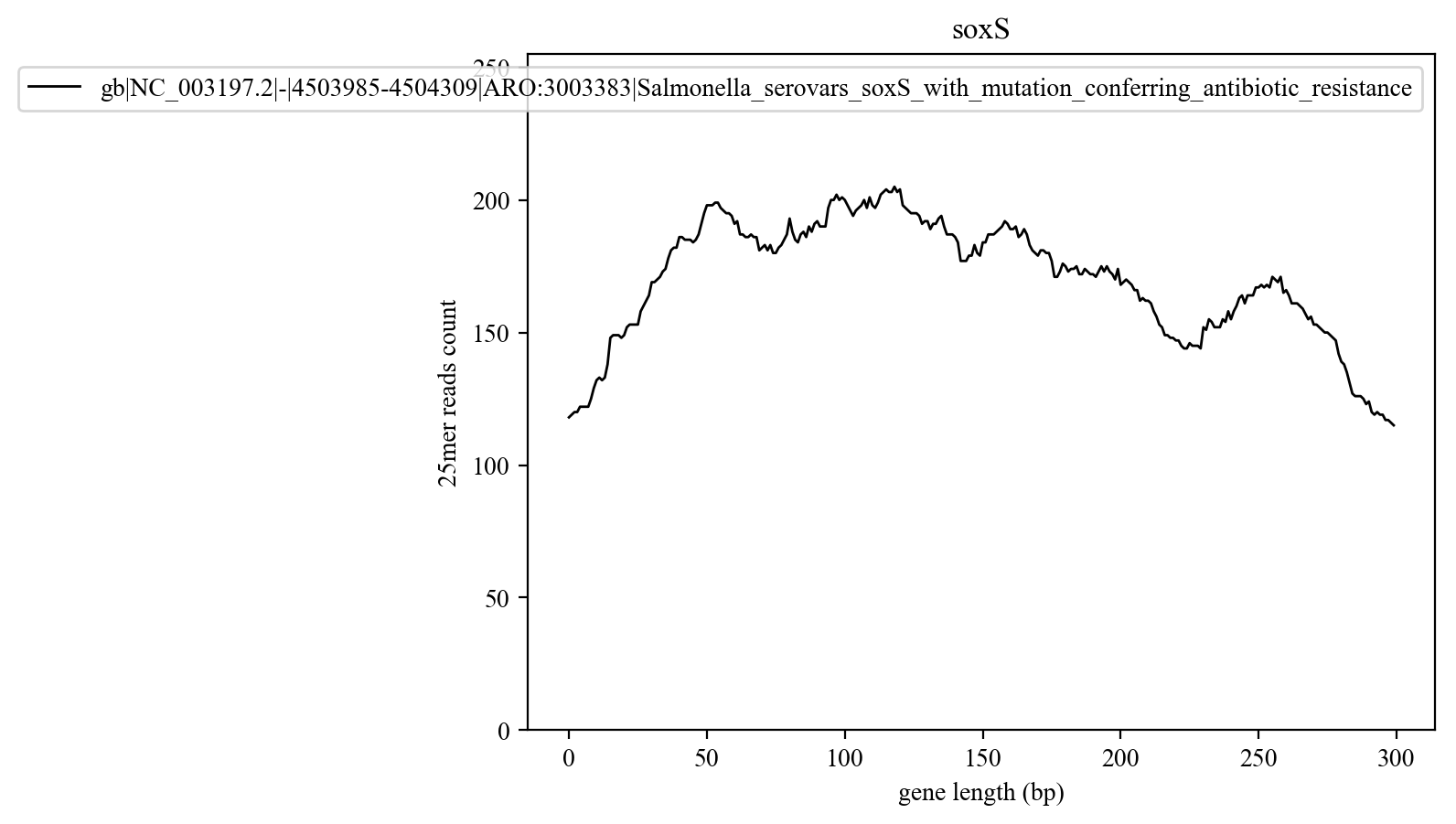

Supplement: Supplementary file 2 — Additional file 2. Archive containing files for evaluation k-mer performance and scoring generated by the k-mer method. [file 12859_2019_3335_MOESM2_ESM.zip › kmer/SJTUF10250_Typhimurium/ar_nucl_25/soxS_25mer.png]

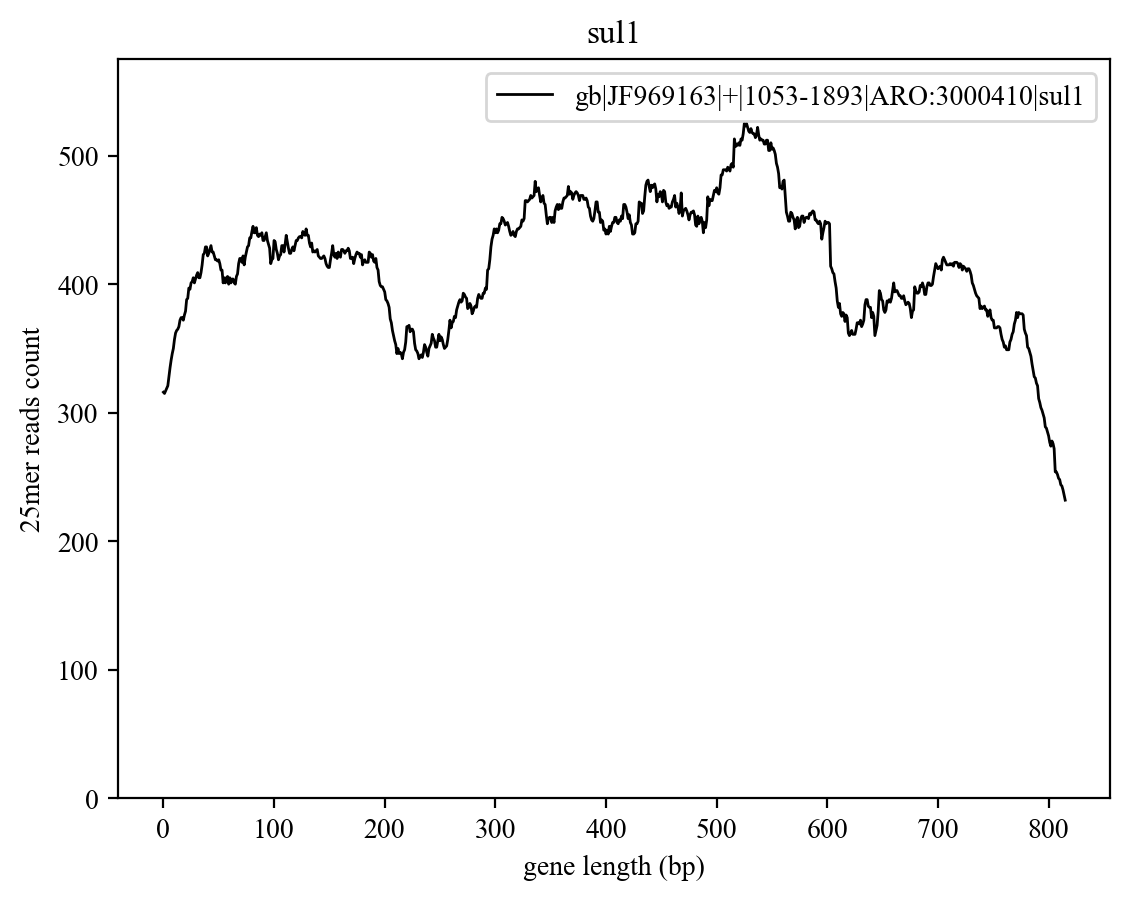

Supplement: Supplementary file 2 — Additional file 2. Archive containing files for evaluation k-mer performance and scoring generated by the k-mer method. [file 12859_2019_3335_MOESM2_ESM.zip › kmer/SJTUF10250_Typhimurium/ar_nucl_25/sul1_25mer.png]

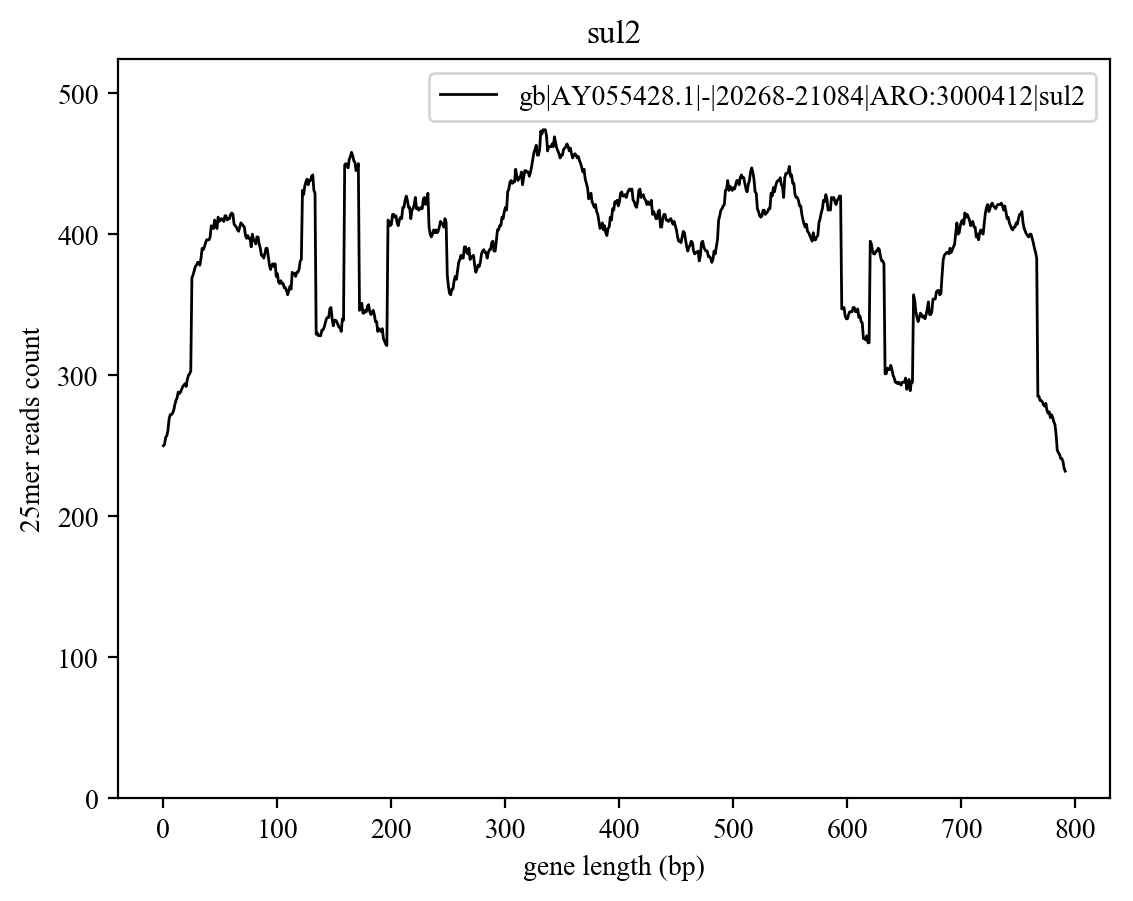

Supplement: Supplementary file 2 — Additional file 2. Archive containing files for evaluation k-mer performance and scoring generated by the k-mer method. [file 12859_2019_3335_MOESM2_ESM.zip › kmer/SJTUF10250_Typhimurium/ar_nucl_25/sul2_25mer.png]

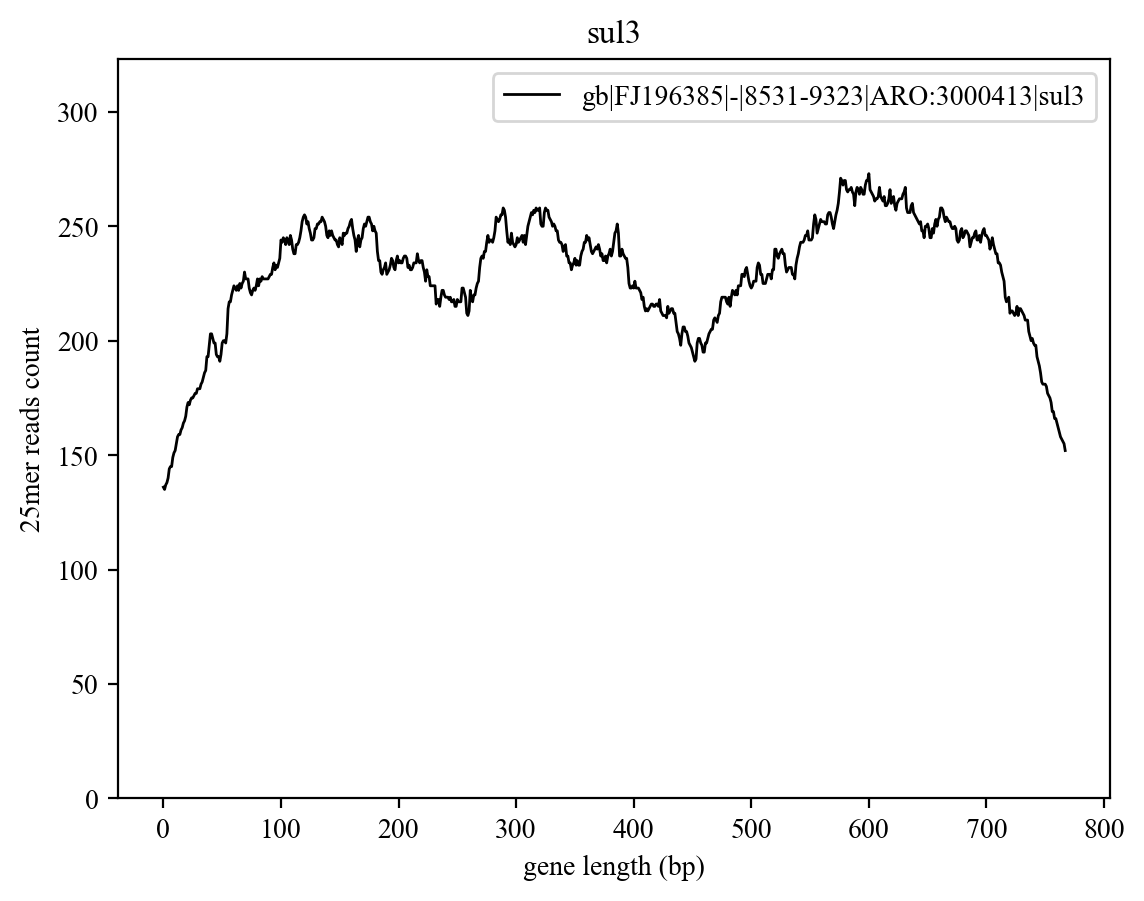

Supplement: Supplementary file 2 — Additional file 2. Archive containing files for evaluation k-mer performance and scoring generated by the k-mer method. [file 12859_2019_3335_MOESM2_ESM.zip › kmer/SJTUF10250_Typhimurium/ar_nucl_25/sul3_25mer.png]

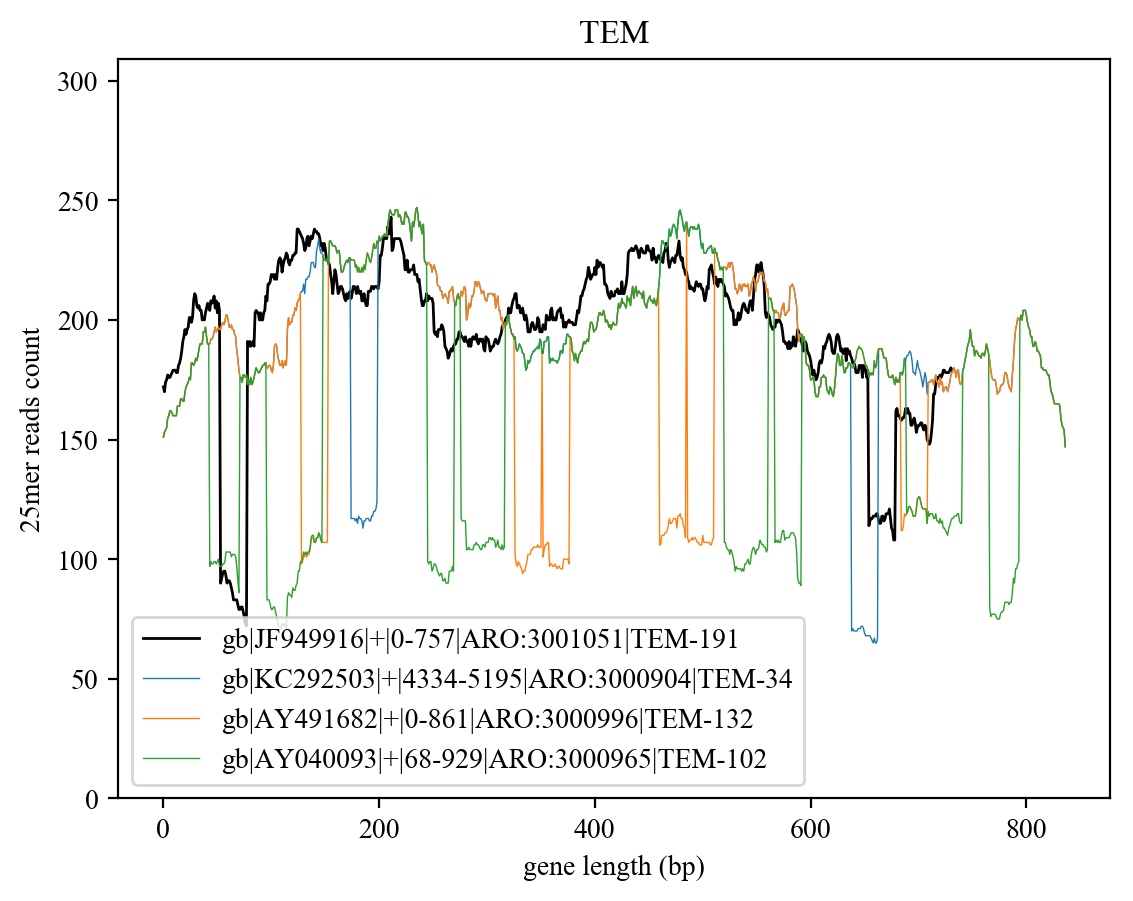

Supplement: Supplementary file 2 — Additional file 2. Archive containing files for evaluation k-mer performance and scoring generated by the k-mer method. [file 12859_2019_3335_MOESM2_ESM.zip › kmer/SJTUF10250_Typhimurium/ar_nucl_25/TEM_25mer.png]

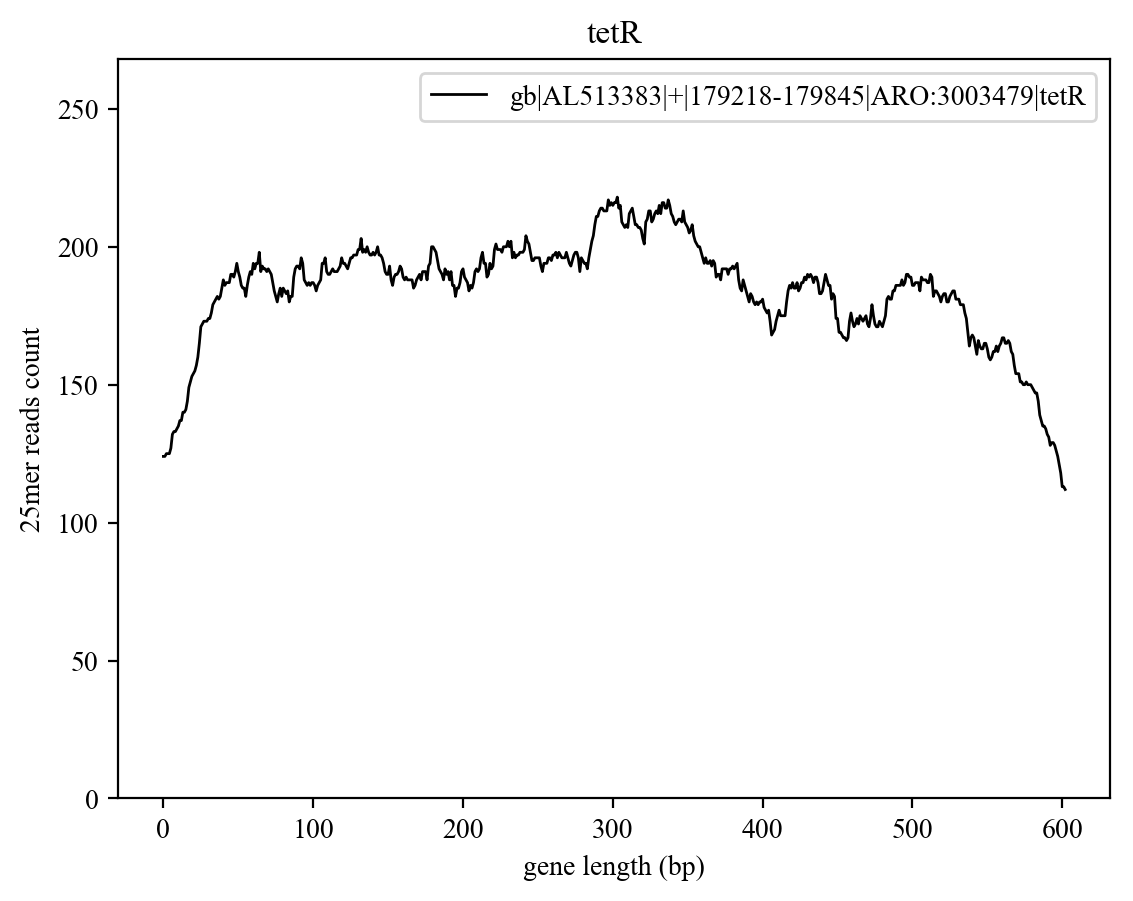

Supplement: Supplementary file 2 — Additional file 2. Archive containing files for evaluation k-mer performance and scoring generated by the k-mer method. [file 12859_2019_3335_MOESM2_ESM.zip › kmer/SJTUF10250_Typhimurium/ar_nucl_25/tetR_25mer.png]

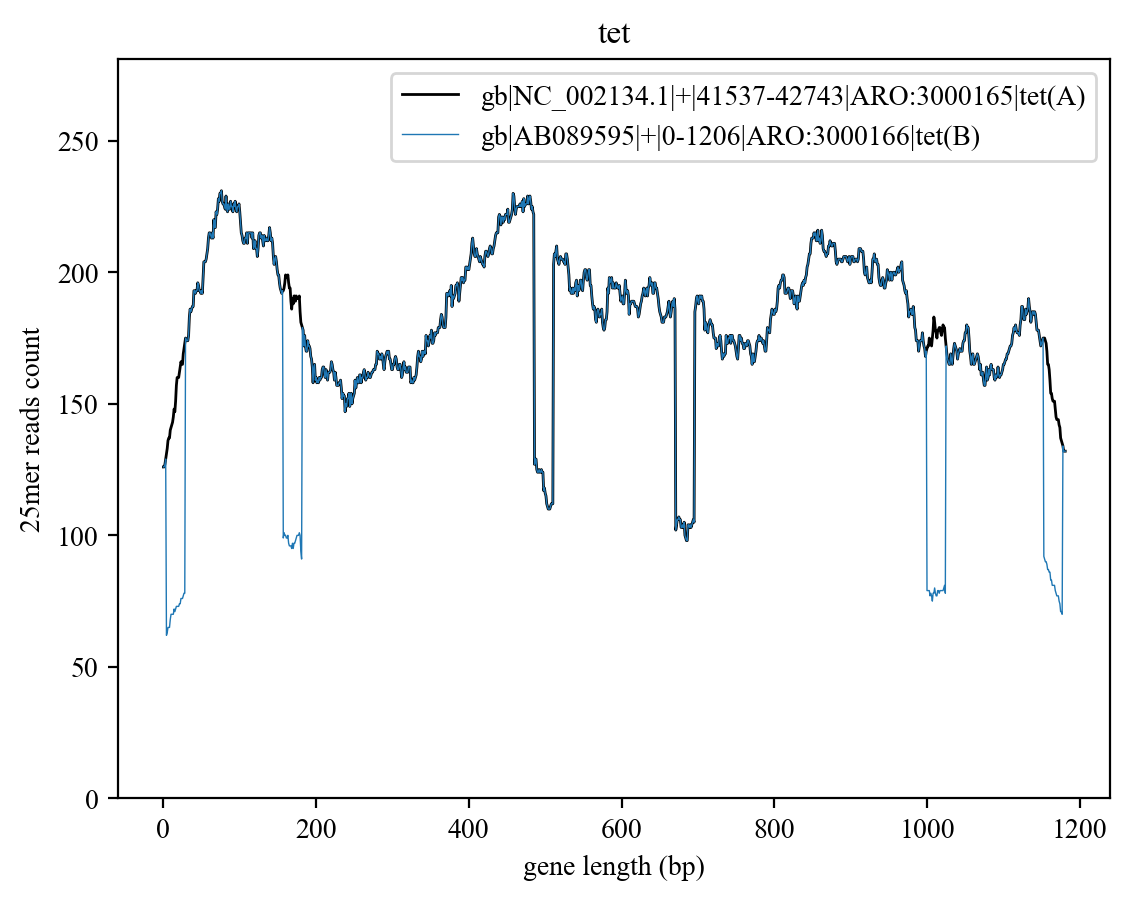

Supplement: Supplementary file 2 — Additional file 2. Archive containing files for evaluation k-mer performance and scoring generated by the k-mer method. [file 12859_2019_3335_MOESM2_ESM.zip › kmer/SJTUF10250_Typhimurium/ar_nucl_25/tet_25mer.png]

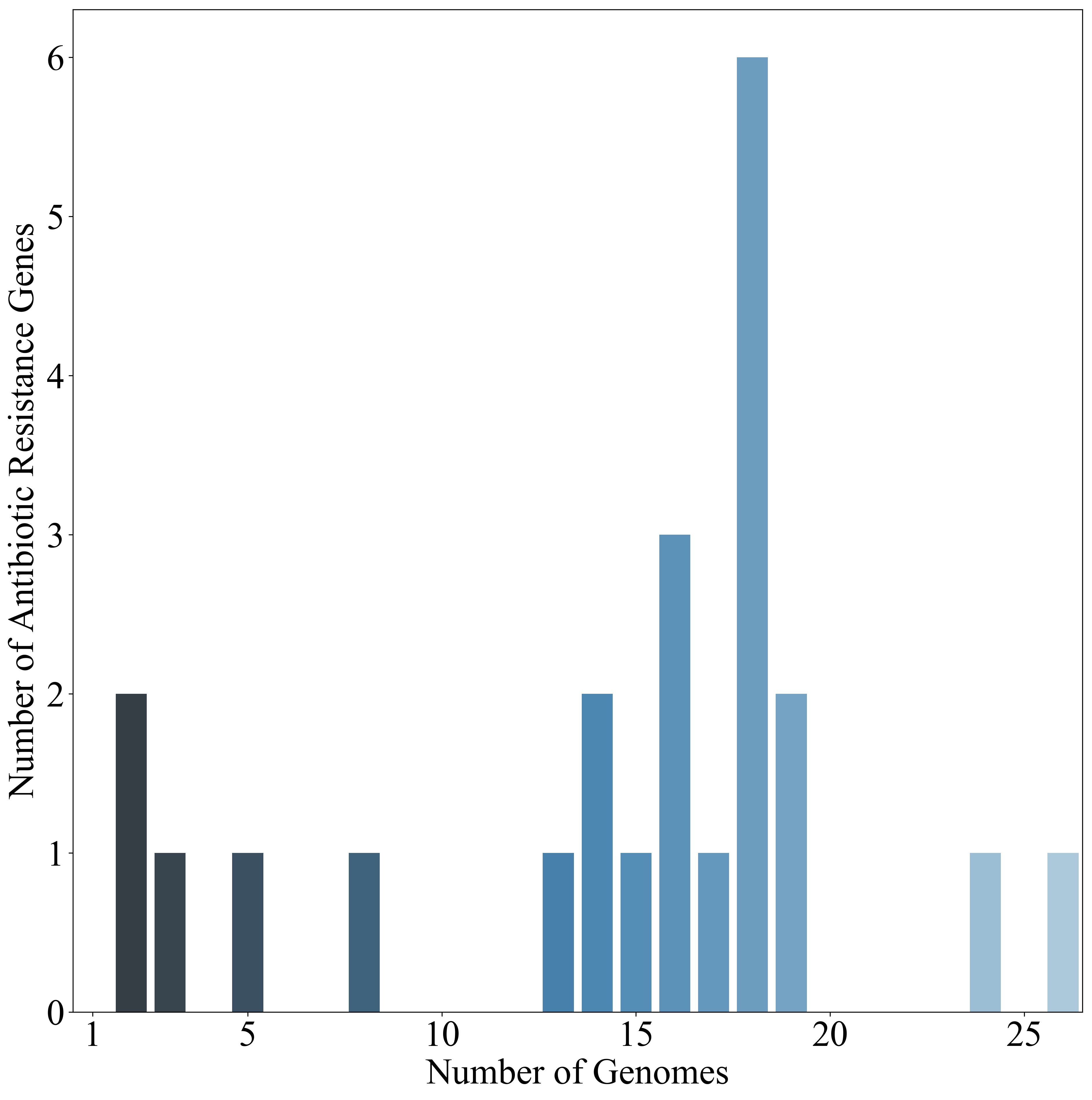

Supplement: Supplementary file 4 — Additional file 4. Archive containing results of analysis for nucleotide sequences of 26 S. enterica genomes annotated by the ResFinder database. [file 12859_2019_3335_MOESM4_ESM.zip › pangenome/4_ar_distribution.png]

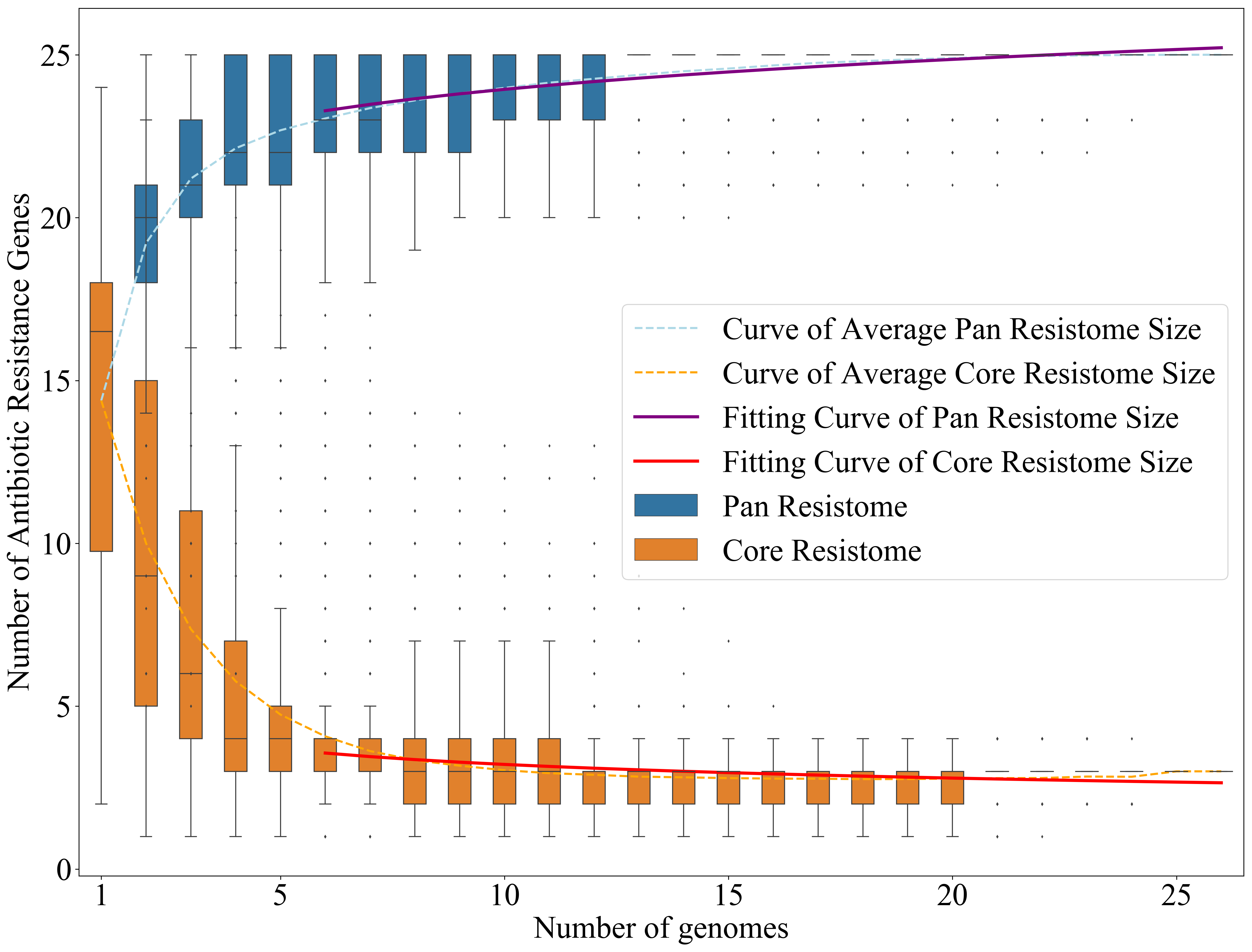

Supplement: Supplementary file 4 — Additional file 4. Archive containing results of analysis for nucleotide sequences of 26 S. enterica genomes annotated by the ResFinder database. [file 12859_2019_3335_MOESM4_ESM.zip › pangenome/5_pangenome.png]

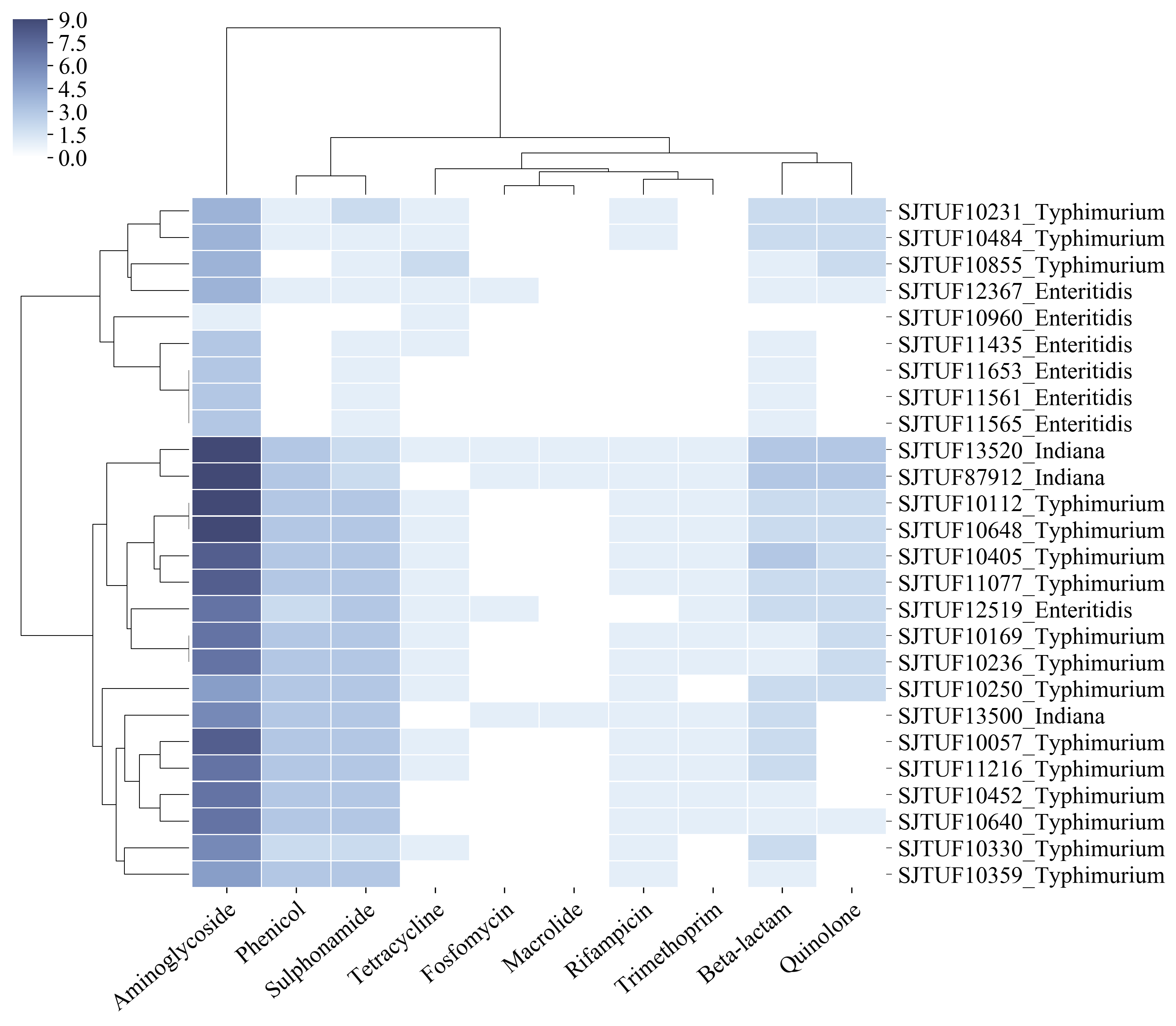

Supplement: Supplementary file 4 — Additional file 4. Archive containing results of analysis for nucleotide sequences of 26 S. enterica genomes annotated by the ResFinder database. [file 12859_2019_3335_MOESM4_ESM.zip › analysis/1_ar_cluster.png]

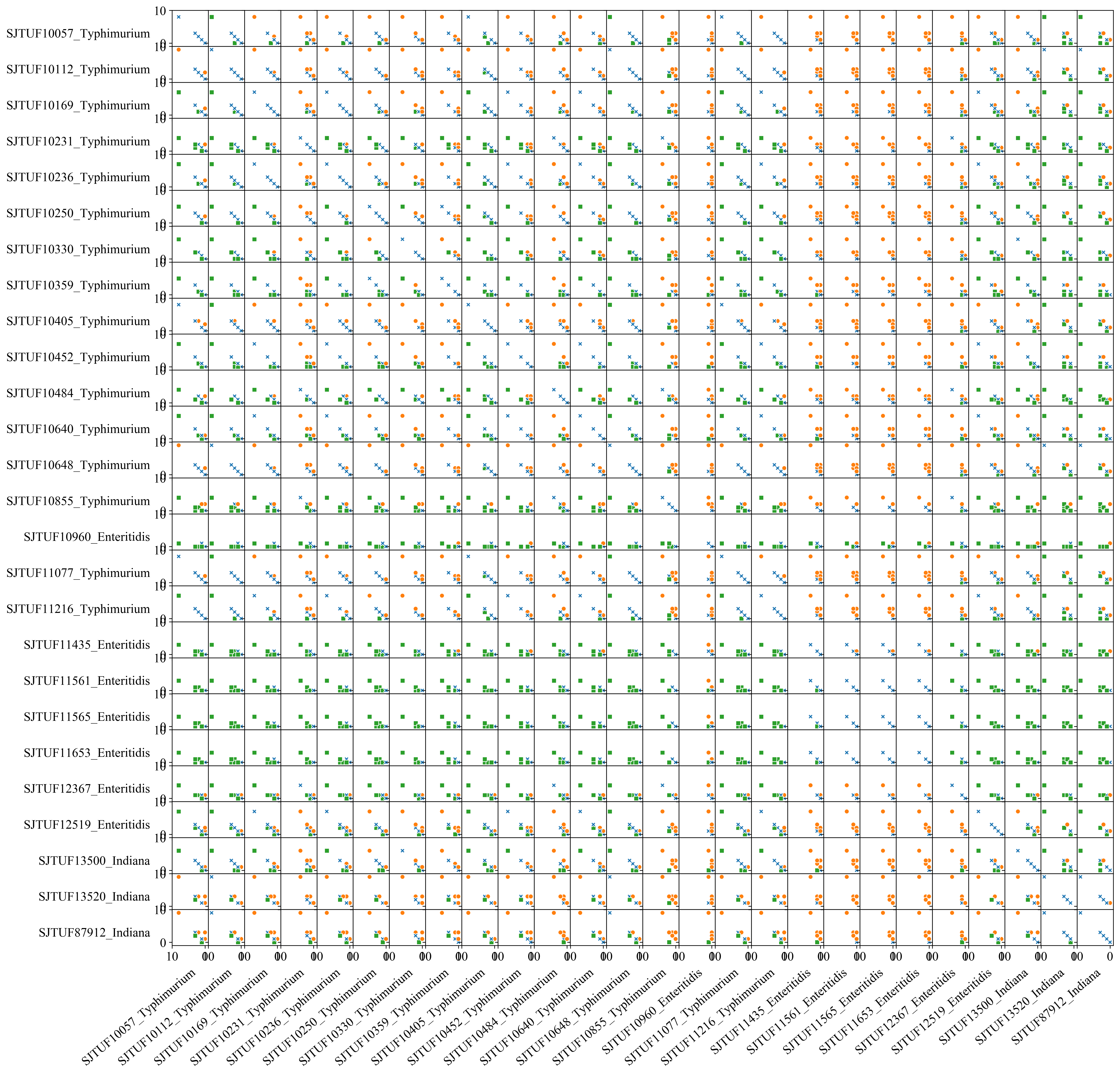

Supplement: Supplementary file 4 — Additional file 4. Archive containing results of analysis for nucleotide sequences of 26 S. enterica genomes annotated by the ResFinder database. [file 12859_2019_3335_MOESM4_ESM.zip › analysis/1_ar_corr.png]

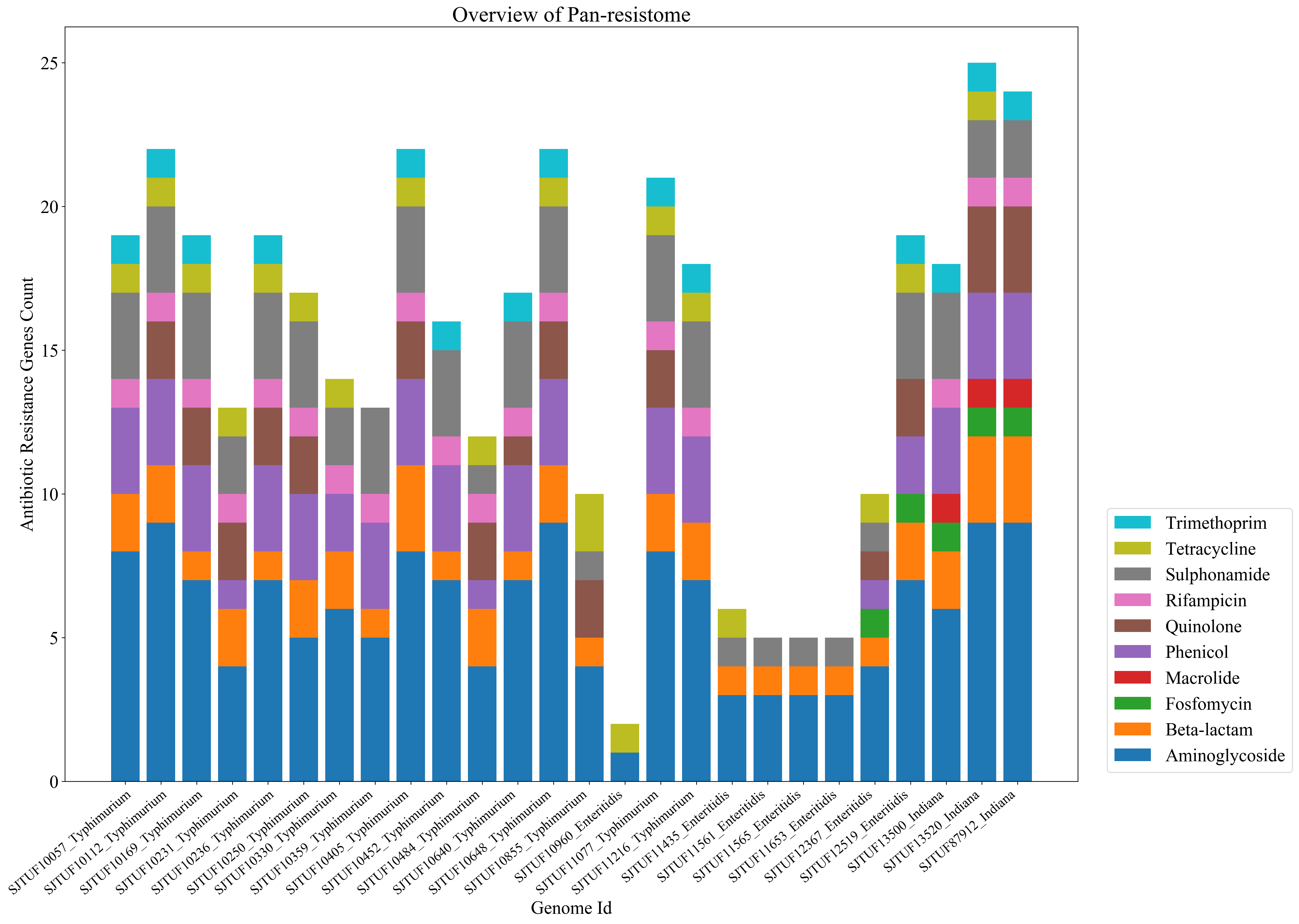

Supplement: Supplementary file 4 — Additional file 4. Archive containing results of analysis for nucleotide sequences of 26 S. enterica genomes annotated by the ResFinder database. [file 12859_2019_3335_MOESM4_ESM.zip › analysis/1_class_summary.png]
