# Supplementary figures and images for: PRAP: Pan Resistome analysis pipeline (part 3 of 3)
Source: BMC Bioinformatics. 2020 Jan 15;21:20. doi: 10.1186/s12859-019-3335-y (PMC6964052; doi:10.1186/s12859-019-3335-y)

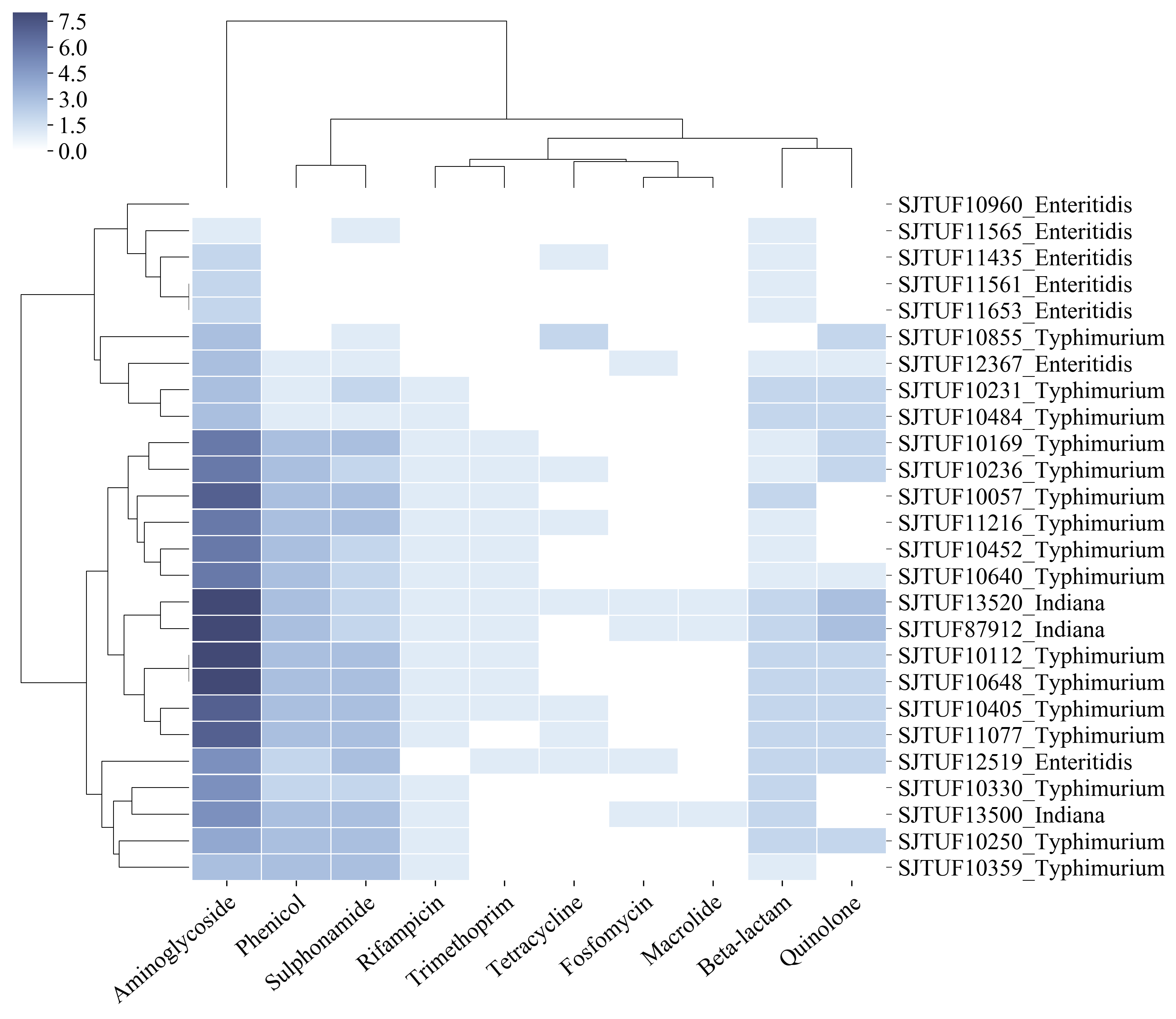

Supplement: Supplementary file 4 — Additional file 4. Archive containing results of analysis for nucleotide sequences of 26 S. enterica genomes annotated by the ResFinder database. [file 12859_2019_3335_MOESM4_ESM.zip › analysis/2_accessory_ar_cluster.png]

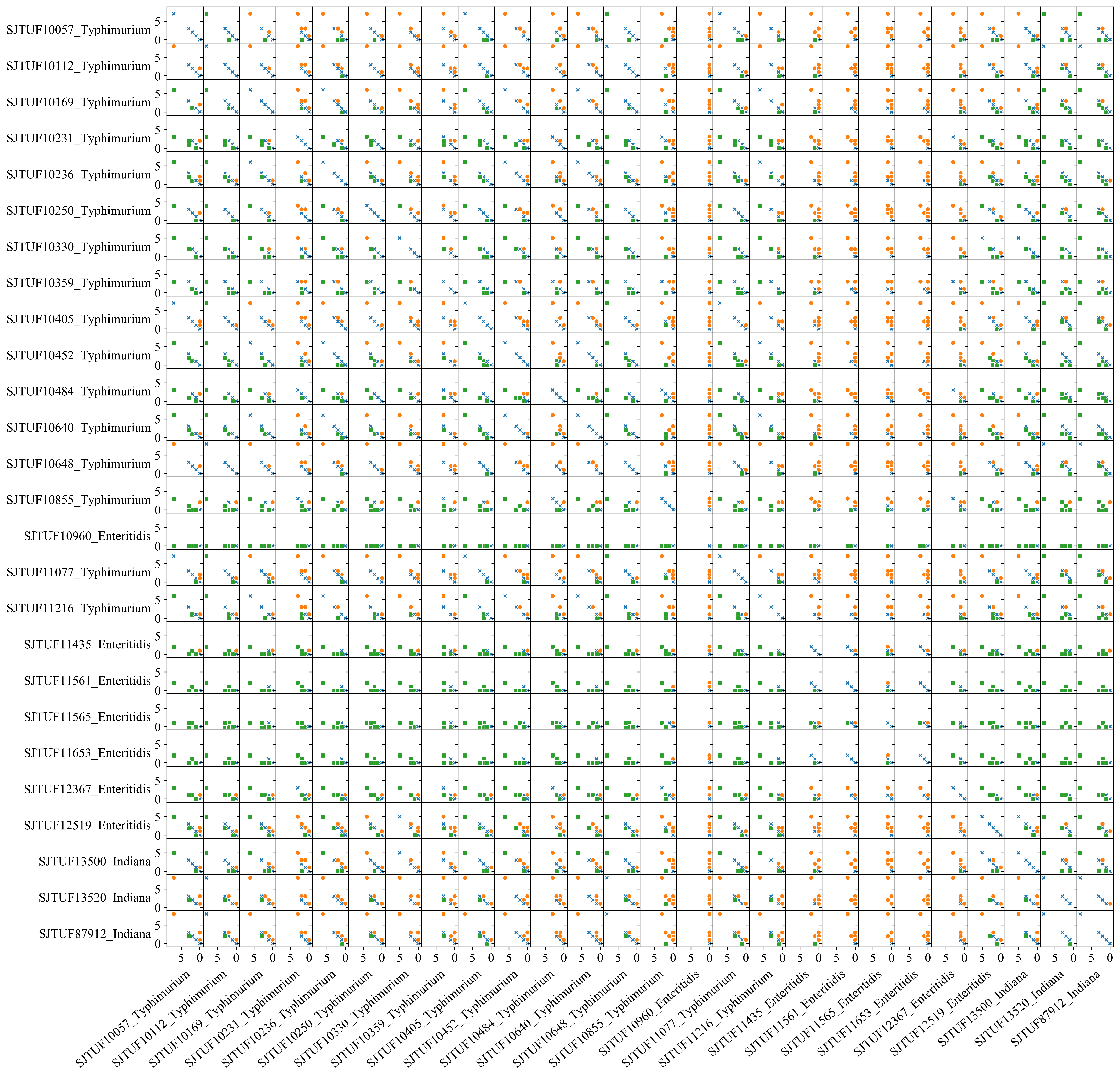

Supplement: Supplementary file 4 — Additional file 4. Archive containing results of analysis for nucleotide sequences of 26 S. enterica genomes annotated by the ResFinder database. [file 12859_2019_3335_MOESM4_ESM.zip › analysis/2_accessory_ar_corr.png]

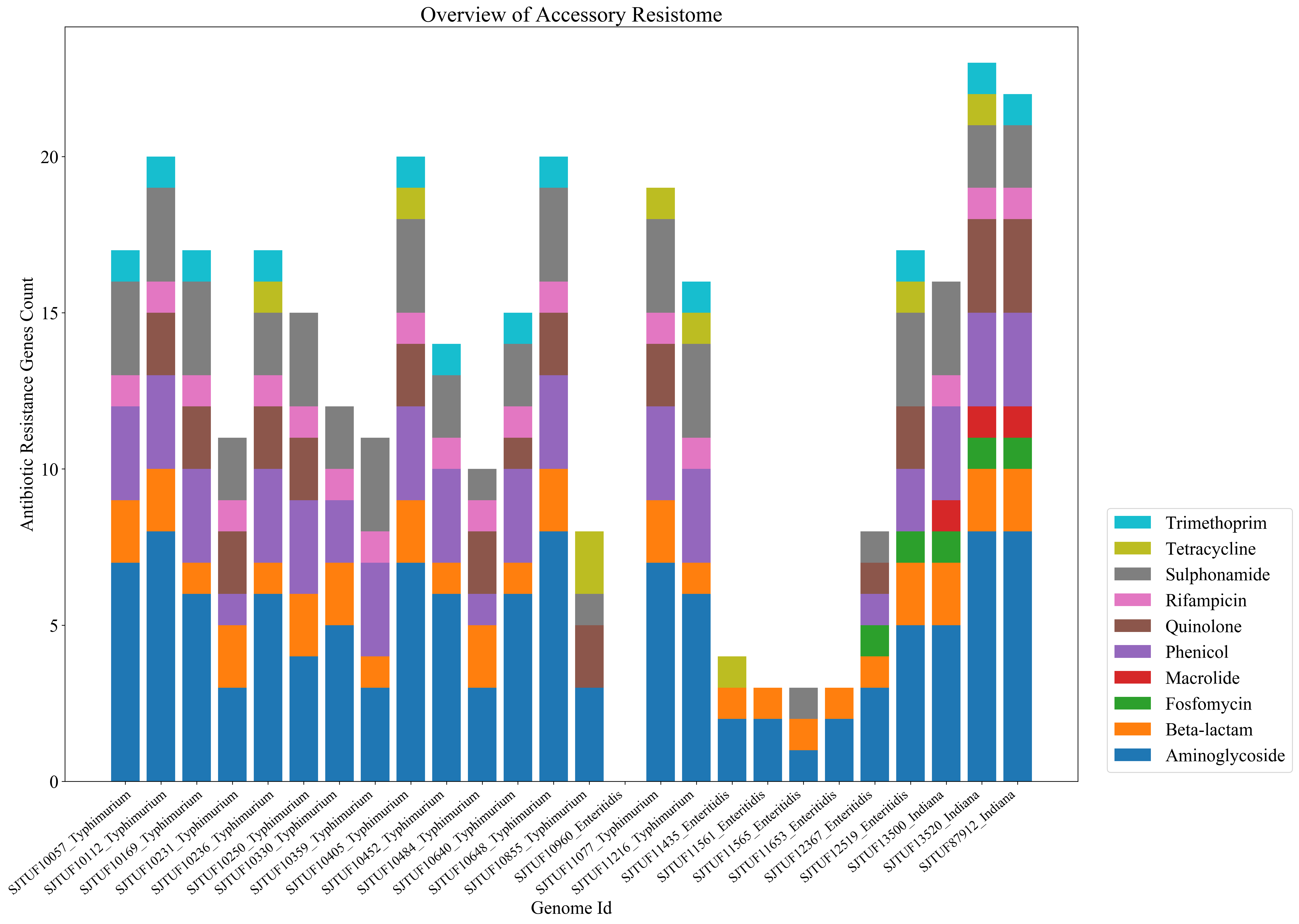

Supplement: Supplementary file 4 — Additional file 4. Archive containing results of analysis for nucleotide sequences of 26 S. enterica genomes annotated by the ResFinder database. [file 12859_2019_3335_MOESM4_ESM.zip › analysis/2_accessory_class_summary.png]

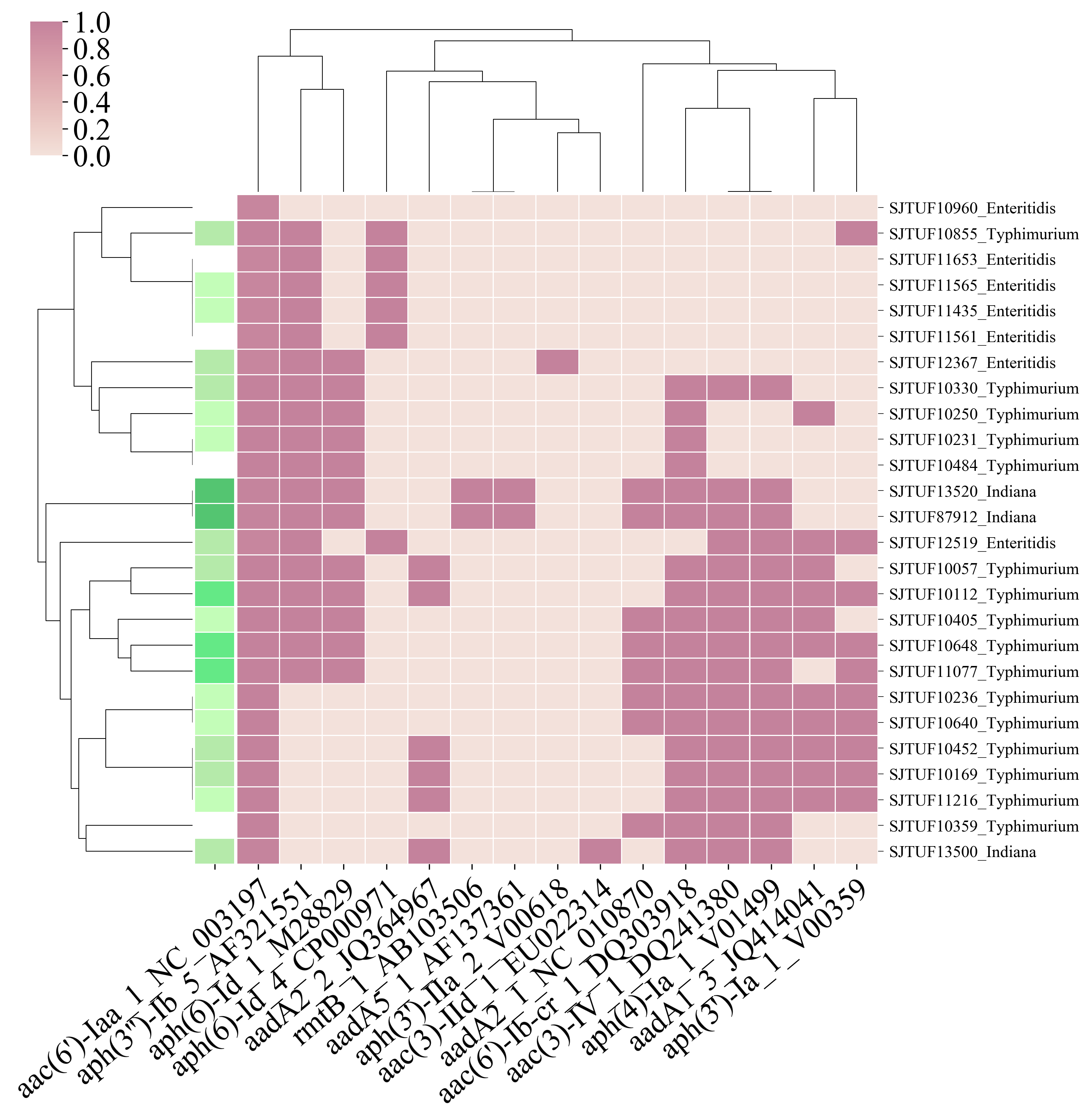

Supplement: Supplementary file 4 — Additional file 4. Archive containing results of analysis for nucleotide sequences of 26 S. enterica genomes annotated by the ResFinder database. [file 12859_2019_3335_MOESM4_ESM.zip › analysis/3_Aminoglycoside_matrix.png]

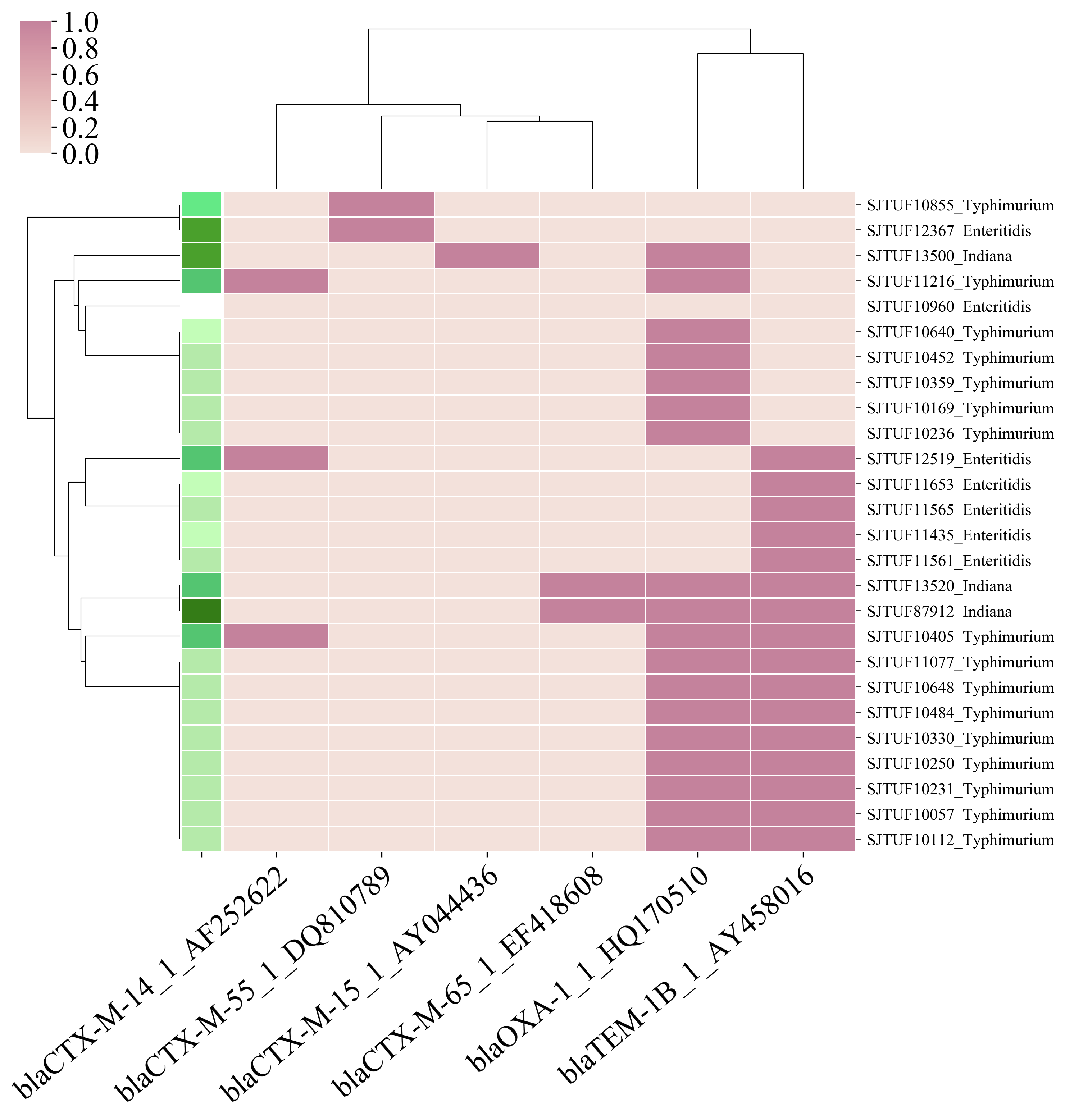

Supplement: Supplementary file 4 — Additional file 4. Archive containing results of analysis for nucleotide sequences of 26 S. enterica genomes annotated by the ResFinder database. [file 12859_2019_3335_MOESM4_ESM.zip › analysis/3_Beta-lactam_matrix.png]

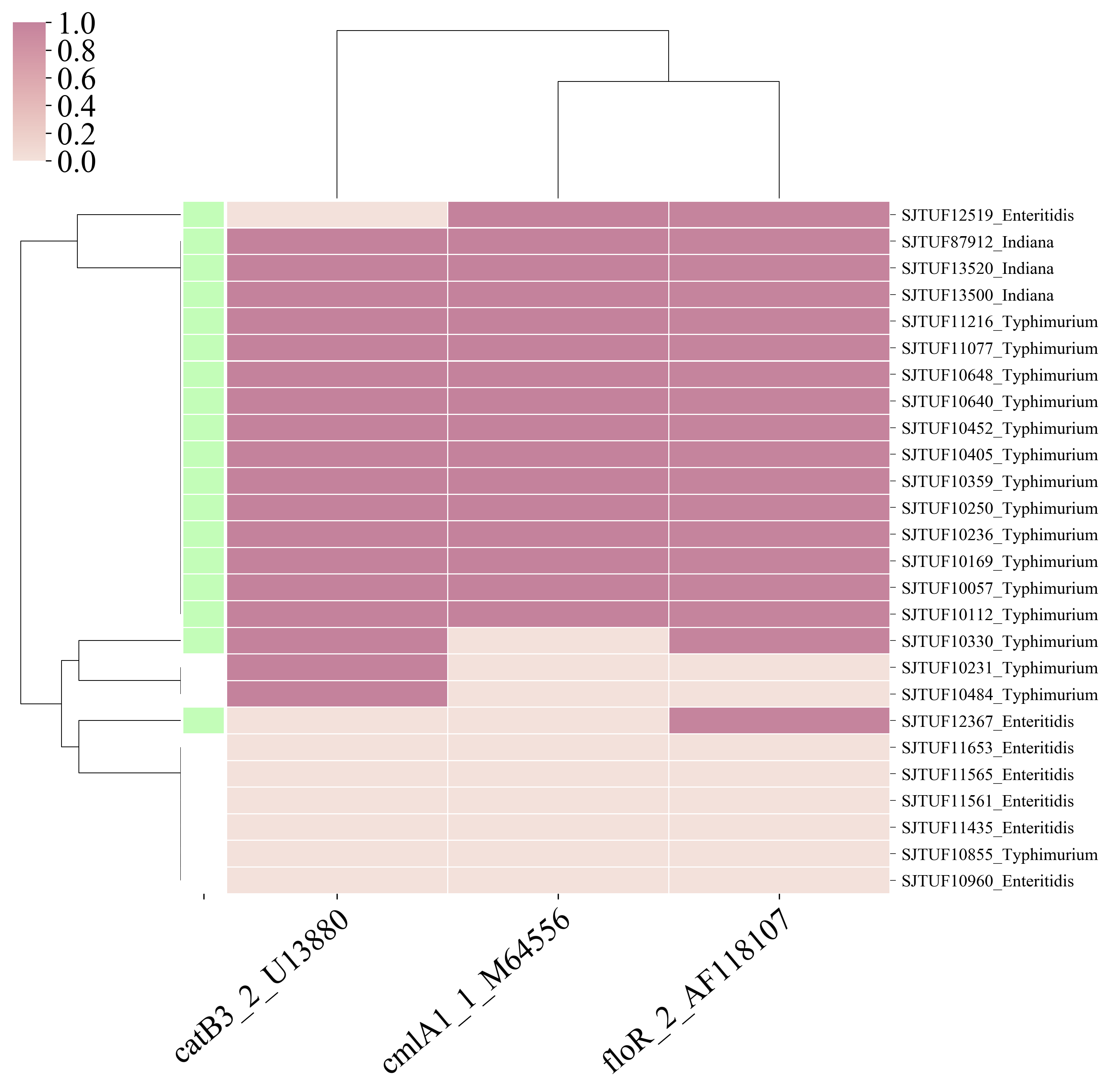

Supplement: Supplementary file 4 — Additional file 4. Archive containing results of analysis for nucleotide sequences of 26 S. enterica genomes annotated by the ResFinder database. [file 12859_2019_3335_MOESM4_ESM.zip › analysis/3_Phenicol_matrix.png]

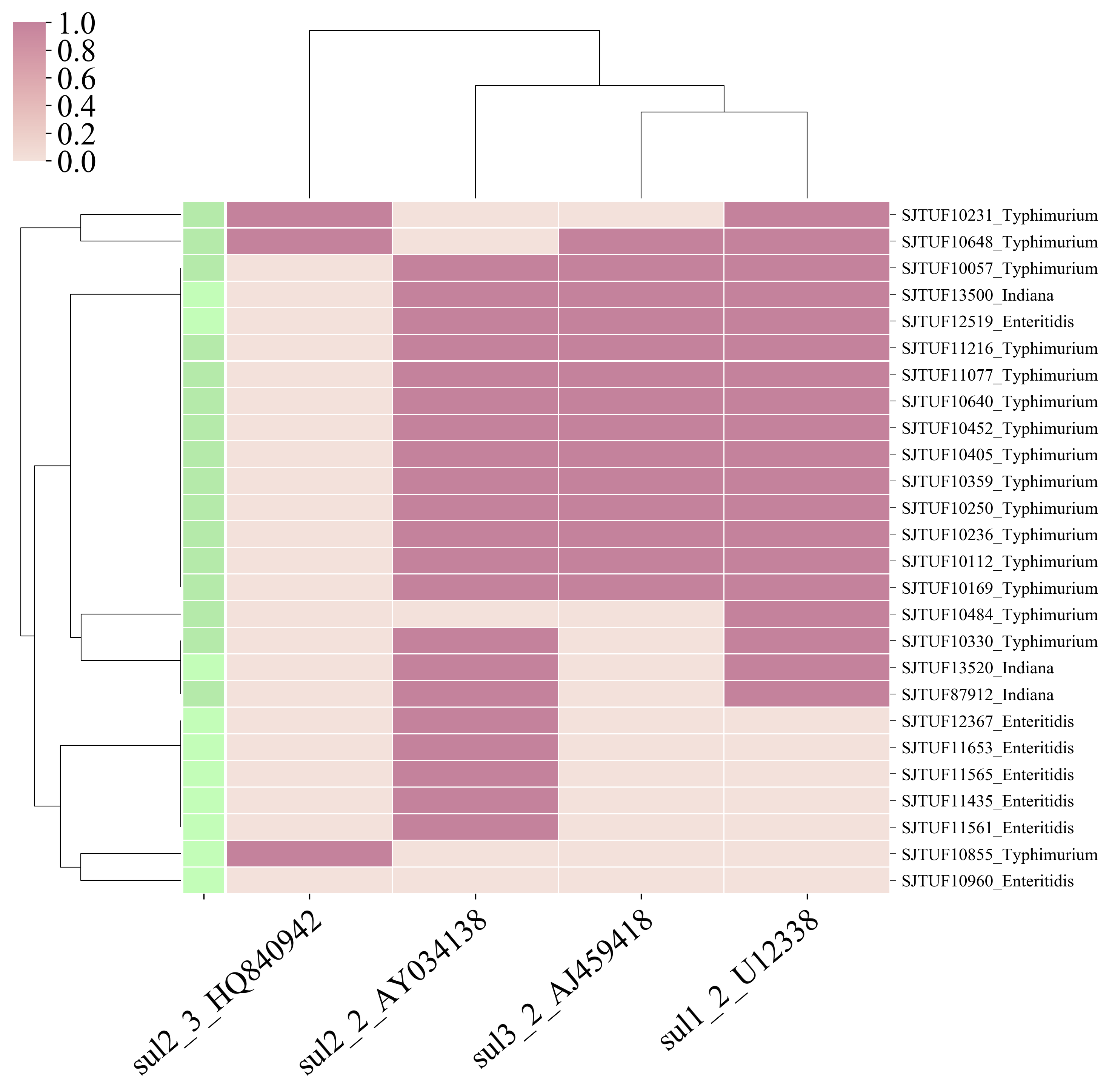

Supplement: Supplementary file 4 — Additional file 4. Archive containing results of analysis for nucleotide sequences of 26 S. enterica genomes annotated by the ResFinder database. [file 12859_2019_3335_MOESM4_ESM.zip › analysis/3_Sulphonamide_matrix.png]

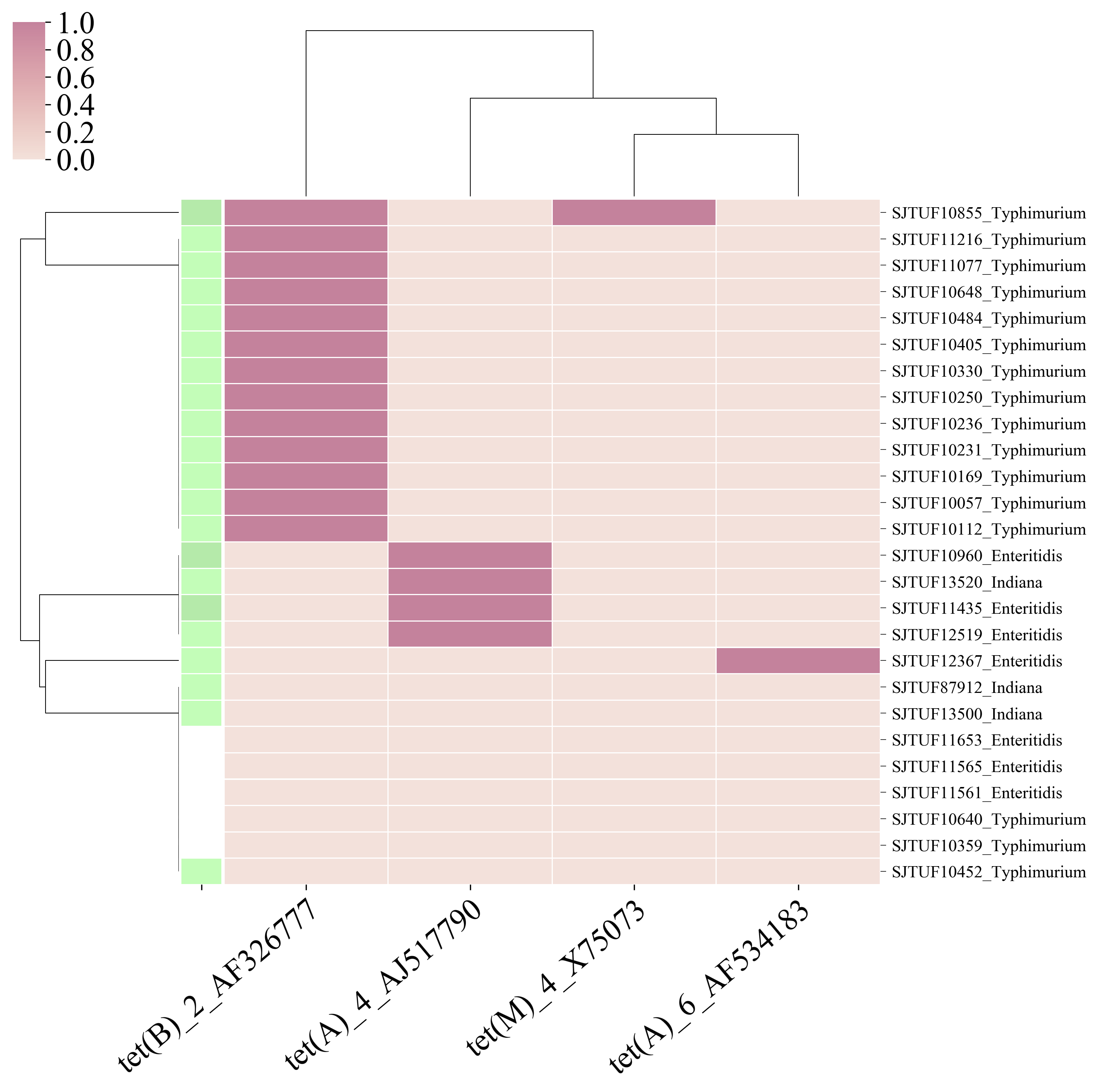

Supplement: Supplementary file 4 — Additional file 4. Archive containing results of analysis for nucleotide sequences of 26 S. enterica genomes annotated by the ResFinder database. [file 12859_2019_3335_MOESM4_ESM.zip › analysis/3_Tetracycline_matrix.png]
